# Training, practice, and career considerations in forensic psychology: results from a field survey of clinical and non-clinical professionals in the United States

**DOI:** 10.3389/fpsyg.2024.1439874

**Published:** 2024-11-12

**Authors:** Casey LaDuke, David DeMatteo, Eve M. Brank, Antoinette Kavanaugh

**Affiliations:** ^1^Department of Psychology, John Jay College of Criminal Justice and the Graduate Center, City University of New York, New York, NY, United States; ^2^Private Practice, New York, NY, United States; ^3^Department of Psychological and Brain Sciences and Thomas R. Kline School of Law, Drexel University, Philadelphia, PA, United States; ^4^Private Practice, Philadelphia, PA, United States; ^5^Department of Psychology, Center on Children, Families, and the Law, College of Law, University of Nebraska-Lincoln, Lincoln, NE, United States; ^6^Feinberg School of Medicine, Northwestern University, Chicago, IL, United States; ^7^Private Practice, Chicago, IL, United States

**Keywords:** forensic psychology, training, practice, career, income, debt, satisfaction

## Abstract

The current field survey describes the identities, training, practices, and careers of 351 U.S. forensic psychologists. Findings are presented for clinical forensic psychologists (*n* = 323) with additional consideration for those working in institutions (*n* = 119), private practice (*n* = 107), or both (*n* = 90), and separately for non-clinical forensic psychologists (*n* = 35). The sample was predominantly middle-aged, White, and female. Participants reported various training paths to the field, and professional settings and activities in the field. Student loan debt was common and significant in clinical Forensic psychologists. Income was generally high, with trends in higher incomes for private practice, board certification, urban areas, and certain geographic regions. Gendered income disparities were common, particularly among those later in their careers and in private practice, with relative parity only observed among early-career clinical forensic psychologists in institutions. Career satisfaction was generally high, with some important barriers noted. Overall, the limited representation of those with historically marginalized cultural identities restricted further consideration and understanding of these important factors in the field. Additional data and discussion are provided for these and other areas of demographics and lived experiences, training and related financial considerations, professional practices (including settings, activities, and clinical test use), and career considerations (including income, benefits, retirement planning, and satisfaction). Together, these data and related discussions offer important insights for prospective and current trainees entering the field, professionals seeking to navigate and advance their careers, and field leaders aiming to contribute to the ongoing development of forensic psychology.

## Introduction

The purpose of the current field survey is to betterchology (American Psychological Association, 2013), “*forensic psychology* refers to professional practice by any psychologist working within any sub-discipline of psychology (e.g., clinical, developmental, social, cognitive) when applying the scientific, technical, or specialized knowledge of psychology to the law to assist in addressing legal, contractual, and administrative matters” (p. 7). By definition, the *field* of forensic psychology refers to anyone engaging in this practice.

Forensic psychology is an established and still rapidly advancing subspecialty in psychology (DeMatteo and Scherr, 2023; Grisso and Brodsky, 2018), the development of which has been partly aided by surveys about professional practice and training (American Psychological Association, 2013; Heilbrun et al., 2008). Earlier surveys focused on more practical topics, such as patterns and opinions regarding psychological test use (Borum and Grisso, 1995). However, these surveys were not designed to inform about or track demographics and lived experiences, training, professional practice, or other career considerations such as debt, income, benefits, and satisfaction. More recently, surveys have addressed these and other topics among specific groups in the field including trainees (American Psychology-Law Society Student Committee, 2015), early career professionals (Cantone et al., 2019), and clinical forensic psychologists (Neal and Line, 2022). Despite these recent efforts, very little is known about mid- and later-career groups, trends across career stages, and non-clinical psychologists in general. Overall, there remains much to discover about those who engage in training, research, teaching, and practice in forensic psychology.

One significant knowledge gap involves financial, personal, and other career considerations among forensic psychologists, such as income and satisfaction. Previous surveys predominantly focused on detailing student loan debt related to professional training (American Psychology-Law Society Student Committee, 2015; Cantone et al., 2019) and only recently included any data on income resulting from professional practice (Neal and Line, 2022). However, there is a notable absence of research that moves beyond basic descriptions of the financial aspects of forensic psychologists, or explores additional considerations related to wellbeing and financial planning. Although data related to finances and related considerations are available for the broader field of psychology (APA, 2017, 2019; Doran et al., 2016) and allied subspecialties (e.g., neuropsychology; Sweet et al., 2021), the application of such data by forensic psychologists is less clear due to meaningful differences in the specialized training and practices that define their subspecialty (American Psychological Association, 2013).

The overarching aim of the current field survey was to provide data and related discussion about forensic psychologists practicing in the United States. This includes insights into demographics and lived experiences, past training and related financial considerations such as stipends and student loan debt, current professional practices, and career considerations such as income, benefits, retirement, and satisfaction. In doing so, the current survey aimed to replicate, extend, and address gaps in previous field surveys, and establish a robust foundation for future research. Most importantly, these insights are intended to support prospective and current trainees as they consider or enter the field, current professionals as they work to develop successful and satisfying careers, and leaders as they advance the field and practice of forensic psychology.

## Materials and methods

Forensic psychologists from the US were surveyed about demographics and lived experiences, training, professional practice, financial considerations, and wellbeing. The survey followed established best practices in survey research (American Association for Public Opinion Research, 2022; Teitcher et al., 2015) and drew on similar surveys in related fields (e.g., Sweet et al., 2015). Approval was granted by the City University of New York Human Research Protection Program (#2019-0268).

### Procedure

Data collection occurred between 4/29/19 and 6/24/19. An invitation and one reminder were sent to several electronic mailing lists selected to support broad representation from the field of forensic psychology, specifically: American Academy of Forensic Psychology (AAFP; *N* ~ 350 at the time of data collection), American Psychology-Law Society (AP-LS; *N* ~ 2,100), American Society of Trial Consulting (ASTC; *N* ~ 200), CONCEPT Professional Training (*N* ~ 30,000), PsyLawList (*N* ~ 800), Div42 listserv [hosted by Community for Psychologists in Independent Practice (APA Division 42); *N* ~ 1,000], and HSP-Community listserv [hosted by the National Register of Health Service Psychologists (NRHSP); *N* ~ 1,100]. Memberships on these mailing lists were not mutually exclusive and included a total reach of ~35,550 overlapping contacts.

Eligible individuals were at least age 18 and involved in forensic psychology training, research, teaching, and/or practice during 2018. Potential participants completed initial eligibility items, and eligible participants were asked to complete the full survey. Participants were compensated by deciding how to donate $1,000 among several pre-selected charitable funds relevant to forensic psychology. The charitable funds (and donation amounts) were the AP-LS general fund ($627), APA Division 42 Psychologists in Independent Practice Next Generation Fund ($143), NRHSP Doctoral Student Credentialing Scholarships fund ($137), and ASTC general fund ($93).

Various methods were incorporated into the study design and survey settings to maximize the number of valid responses (Teitcher et al., 2015). Recruitment consisted of field-specific distribution of the research solicitation via relevant professional organizations. Distribution was based on a survey link provided in the research solicitation itself to control how participants accessed the survey, which was summarily tracked throughout data collection using search engines to confirm the survey link had not been posted elsewhere. Informed consent used clear language regarding eligibility and specifically stated that participants could only complete the survey once. Compensation was clearly described in the informed consent and designed to be indirect and altruistic in nature. Survey settings were activated to prevent multiple submissions by the same respondent and indexing of the survey on search engines.

### Sample

Of the 561 initial survey responses, 210 were ultimately excluded from further analysis. Exclusions occurred due to non-consent (*n* = 2), failure to respond to age eligibility (*n* = 2), and non-response or ineligibility regarding forensic practice generally (*n* = 13) or during the target year of 2018 (*n* = 17). Additionally, methods from Teitcher et al. (2015) were applied to identify and exclude duplicates. Specifically, responses were flagged for further review based on non-unique IP addresses (*n* = 74), similar timestamps (*n* = 8), or completion times beyond two standard deviations from the trimmed mean (*n* = 34); the first author (CL) reviewed these flagged responses, resulting in the exclusion of a minority (*n* = 11 of flagged responses) as duplicates.

Additional exclusions were made for inadequate data beyond eligibility (*n* = 15), compensation (*n* = 2), or items required for forensic categorization (see below for more detail; *n* = 54). Those practicing outside the US were excluded for their limited sample sizes and distinct practical and financial considerations, including those who resided abroad in 2018 (*n* = 59), moved to the US in 2018 (*n* = 1), or did not provide sufficient information (*n* = 3). Participants categorized as legal professionals only (*n* = 2), current students (*n* = 14), or students transitioning into the workforce in 2018 (*n* = 15) were also excluded for their limited sample sizes and notable differences from professional groups included in further analyses. The final sample consisted of 351 forensic psychologists from the US who were consented, eligible, unique, and provided sufficient information.

Participants were next categorized into clinical and non-clinical groups. *Clinical* was operationalized to denote professionals engaged in assessment, intervention, or clinical research, encompassing related designations like applied and counseling psychology. *Non-clinical* was operationalized to denote professionals engaged in aspects of psychology generally described as social, developmental, experimental, cognitive, legal-psychology, or program evaluation. Both terms were chosen for their common use in the field. Categorization involved an iterative and collaborative process among several authors (CL, DD, EB), considering each participant's self-reported categorization, training, and professional activities (reverse-weighted in case of conflicts). The total sample of 351 forensic psychologists was ultimately categorized into *clinical participants* (*n* = 323) and *non-clinical participants* (*n* = 35). These groups were not mutually exclusive (i.e., *n* = 7 categorized as both clinical and non-clinical participants based on their training *and* current practices) and included participants with further legal training or professional activity (*n* = 8 overall; *n* = 6 clinical and legal, *n* = 2 clinical, non-clinical, and legal).

Lastly, clinical participants (*n* = 323) were further categorized based on their self-reported practice setting(s), namely institutional settings (*n* = 119), private practice (*n* = 107), or both (*n* = 90). *Institutional settings* refer to established organizations where professionals are hired and provide professional services (e.g., state hospitals, court clinics, universities). *Private practice* includes self-employed professionals or those in smaller, non-institutional group practice. Examples of clinical participants categorized as both are those who teach at a university or work in a psychiatric hospital, and also run or work in a private practice. Considering these settings aligns with trends in professional practice in the field of forensic psychology, and is further supported by meaningful differences among these settings in the data (see below and Supplementary material). Some clinical participants either chose not to respond to the work setting question (*n* = 3) or did not provide sufficient information (*n* = 4), and are therefore excluded from relevant subanalyses. Limited sample size prevented subanalyses of practice setting for non-clinical participants (*n* = 35).

### Survey

The full survey is provided in the Supplementary material. Items included demographics and lived experiences; past training and related financial considerations, specifically stipends and student debt; current professional practice; and current career considerations, specifically income, benefits, retirement planning, satisfaction, barriers to satisfaction, and perceived stress related to student loan debt and retirement. Exit items gathered opinions on survey representativeness and recommendations for improvement. Specific items were adapted from authoritative sources such as surveys from allied fields (e.g., neuropsychology; Sweet et al., 2015), practice guidelines endorsed by leading organizations (American Psychological Association, 2013), and other widely recognized instruments (Organisation for Economic Co-operation and Development, 2012; United States Census Bureau, 2018). Additional details about specific items are provided below, and throughout the Results section and Supplementary material.

Income was operationalized as total pre-tax income plus deferred money set aside in the same year. The current data include income reported from calendar year 2018 (i.e., 1/1/2018–12/31/2018) earned by the participant (i.e., not that of a spouse or any other joint income) in whole dollar amounts. Participants who did not know specific amounts were asked to provide their best estimates. Those working part-time were asked not to extrapolate to a full-time equivalent. Participants were first asked to report their *overall income*. Following this, they were asked to report income related to three separate sources: forensic psychology, other psychology, and other. *Forensic psychology income* was operationalized as any income related to studying, researching, teaching, and/or practicing in any sub-discipline of psychology where they applied their scientific, technical, or specialized knowledge to any aspect of the law. *Other psychology income* was operationalized as any income related to studying, researching, teaching, and/or practicing in any sub-discipline of psychology that did not include applying their scientific, technical, or specialized knowledge to any aspect of the law. *Other income* was operationalized as any income related to activities that did not include studying, researching, teaching, and/or practicing in any sub-discipline of psychology. For each of these sources, participants were asked to report their income either as a whole dollar amount or as a percentage of their total income. Any percentages were then converted into estimated whole dollar amounts.

Participants reported their satisfaction with *work activities, income*, and *work-life balance* in two ways. First, participants reported their satisfaction in each area on a sliding scale from “completely dissatisfied” (0) to “completely satisfied” (100). Second, participants reported their satisfaction in each area using a five-point Likert scale ranging from “completely dissatisfied” (1) to “completely satisfied” (5). Participants further indicated whether any aspects of their personal life, family life, work activities, other personnel at work, or physical work environment created barriers to attaining greater satisfaction in each area.

## Results

Distinctions are made between clinical participants (*n* = 323) and non-clinical participants (*n* = 35) where relevant. Trends among clinical participants working in institutional settings (*n* = 119), private practice (*n* = 107), or both (*n* = 90) are only included when meaningful differences were observed based on inferential analyses (i.e., η^2^ ≥ 0.06 and Cramér's V ≥ 0.30; Cohen, 1988, 1992; see Supplementary material); otherwise, results are presented for clinical participants overall. Most numbers have been rounded to the nearest integer to increase accessibility to the results. All numbers related to income are based on estimated whole dollar amounts, rounded to the nearest thousand. Refer to Supplementary material for complete data (including effect sizes for group comparisons) and additional notes, with more specific references provided throughout this section whenever relevant.

### Demographics and lived experiences

#### Age, years of experience, and career stage

Age, years of experience, and career stage for clinical and non-clinical participants are summarized in Table 1 (see additional data and notes in Supplementary Table S1.1). Both clinical and non-clinical participants were mostly middle-aged and had been practicing in the field for most of their professional careers. For clinical participants, average age appeared higher across those working in institutions only, both institution/private practice, and private practice only, respectively. A similar trend was observed for years in the field. Most clinical participants working only in institutional settings were in their early career followed by mid-career, with much fewer in their senior career or later senior career. Those working in both institution/private practice settings were more balanced between early and mid-career stages, though still fewer were in their senior career or later senior career. Those only in private practice were comparable across all career stages.

**Table 1:** Selected demographics and lived experiences of forensic psychologists.

	**Overall**	**Clinical**				**Non-Clinical**
		**Overall**	**Professional setting**			
			**Institution only**	**Institution/private practice**	**Private practice only**	
Age [*M* (*SD*)]	48.9 (13.3)	49.0 (13.3)	41.3 (9.3)	50.1 (12.7)	56.6 (13.1)	50.4 (14.4)
Years in the field [*M* (*SD*)]	15.7 (12.4)	15.7 (12.4)	9.2 (8.3)	16.4 (11.6)	22.2 (13.3)	18.4 (14.5)
**Career stage (%)**						
Early	41.4	41.5	64.7	32.6	23.6	34.3
Mid	29.6	29.6	27.7	40.4	23.6	25.7
Senior	13.9	13.2	4.2	11.2	23.6	22.9
Late Senior	15.1	15.7	3.4	15.7	29.2	14.3
**Gender identity (%)** ^*^						
Woman	63.5	62.7	78.4	61.4	47.6	72.7
Man	34.4	35	20.7	37.5	48.5	27.3
**Gender identity, by career stage (% female)**						
Early	52.4	53.4	71.4	41.5	33.3	37.5
Mid	31.1	31.1	25.3	43.4	29.2	29.2
Senior	8.5	7.8	1.1	9.4	16.7	20.8
Late senior	8.0	7.8	2.2	5.7	20.8	12.5
**Cultural identity (%)** ^*^						
White/European American	91.7	91.6	89.7	94.3	93.2	93.9
Latinx	5.3	4.2	3.4	4.5	3.9	15.2
Black/African American	1.8	1.9	4.3	1.1	0	0

Sample sizes generally include all eligible participants in the overall (*n* = 351), clinical (*n* = 323), and non-clinical samples (*n* = 35). Professional Setting further includes clinical participants working in institutional settings only (*n* = 119), both institutional settings and private practice (*n* = 90), or private practice only (*n* = 107). Actual sample sizes reduced due to item non-response. Responses with <5% response rate excluded. Years in the field = estimated based on reported year of highest relevant degree (first degree if multiple) and date of study completion. Early Career = first 10 years since highest degree (first such degree if multiple); Mid-Career = 11–20 years; Senior Career = 21–30 years; Late-Senior Career = 31–50 years. Latinx includes participants who identified as Latina, Latino, Latinx, Hispanic, or any heritage from a Latin American country. ^*^Multiple responses allowed.

#### Gender identity

Gender identity for clinical and non-clinical participants are also summarized in Table 1 (see additional data and notes in Supplementary Table S1.1). Nearly twice as many clinical and non-clinical participants identified as cisgender women compared to cisgender men, with very few identifying with any other gender identities (the shortened terms “women” and “men” will therefore be used moving forward). This imbalance appeared somewhat more prominent among non-clinical participants than clinical participants. Additional trends were observed for profession, career stage, and work setting. Specifically, women comprised the majority of early career clinical participants working only in institutional settings, while men predominated every other career stage and work setting for both clinical and non-clinical participants. This trend was particularly pronounced for clinical participants working in institutional settings, either solely or in conjunction with private practice. Similar but somewhat less dramatic trends were observed for clinical participants working only in private practice settings, and non-clinical participants in general.

#### Self-reported race/ethnicity, citizenship, and language

Self-reported race and ethnicity for clinical and non-clinical participants are also summarized in Table 1 (see additional data and notes in Supplementary Table S1.1). Nearly all clinical and non-clinical participants identified as White, with fewer identifying as Latinx or Black.

Additionally, nearly all forensic psychologists were native US citizens (95% overall; range = 94–97%). Nearly all spoke only English at home (92%; range = 88–93%) (Supplementary Table S1.1).

#### Family of origin

Nearly all clinical and non-clinical participants were raised by both their mother (75% primary caregiver; 24% secondary caregiver) and their father (24% primary; 75% secondary). Most of their caregivers completed college (17% primary; 20% secondary) or professional degrees (32% primary; 25% secondary). Nearly all reported no use of public assistance programs growing up (89% overall; range = 86–93%) (Supplementary Table S1.2).

#### Residency

Clinical and non-clinical participants showed considerable variability within and across groups in terms of the U.S. regions and areas where they grew up and currently reside (see Supplementary Tables S1.2–S1.5). A common trend was movement toward various Atlantic regions and larger urban areas, with notable exceptions. For clinical participants, the most significant movements seemed to be toward Pacific (+10%) and South Atlantic regions (+7%) and away from Middle Atlantic (−10%) and East North Central regions (−8%), as well as toward metropolitan areas (+7%) and away from rural areas (−14%). For non-clinical participants, the most significant movements appeared to be *away* from Pacific (−19%) and West South Central regions (−10%) and *toward* East North Central (+14%) and New England regions (10%), as well as toward larger metropolitan areas (+9%) and away from rural areas (−18%).

#### Relationships

Most clinical and non-clinical participants identified as heterosexual/straight (88% overall; range = 88–93%). Small numbers identifed with other sexual identities (e.g., 9% bisexual for non-clinical participants; 6% lesbian for clinical participants in both institution/private practice; otherwise range = 0–4%) (Supplementary Table S1.1).

Most clinical and non-clinical participants were currently married (75% overall; range = 68–82%), with some gender differences noted for clinical participants (72% women; 82% men) and non-clinical participants (71% women; 100% men). Smaller groups had never been married (14% overall; range 8–21%) with similar trends by gender (clinical 18% women, 8% men; non-clinical 17% women, 0% men), or were currently divorced (7% overall; range = 3–9%). About one-quarter of those married had been more than once (two marriages: 18% overall, range = 11–25%; three or more: 5% overall, range = 2–8%) (Supplementary Table S1.5).

The median household makeup for clinical and non-clinical participants was three people [*interquartile range (IQR)* 2–4]. This most commonly included the participant with their partner (81% overall) and dependent child(ren) (42% overall), with fewer residing with adult child(ren) (5% overall) and much fewer residing with extended family members or others. Among those with dependent children, clinical participants appeared more likely to report residing with two children (50%) compared to one (30%) or three or more (21%), whereas non-clinical participants were more likely to report residing with one (47%) compared to two (29%) or three or more (24%) (Supplementary Table S1.5).

#### Other considerations

Nearly all clinical and non-clinical participants reported no significant functional difficulties (94% overall) and never serving in the military (96% overall) (Supplementary Table S1.1).

#### Training and related financial considerations

More clinical participants reported their highest degree as a PhD (61%) with a sizable group earning a PsyD (35%). Most reported degrees in Clinical Psychology (71%) and degrees from APA/CPA accredited programs (85%). Almost all completed a predoctoral internship (94%), most of which were APA/CPA accredited (85%) (Supplementary Table S2.1).

Among various sources of funding for graduate training, student loans were the only funding source reported by a majority of clinical participants (70%). Additional sizable groups reported stipends (45%; *Mdn* = $12,000, *IQR* 7–17), external employment (38%), teaching assistantships (37%), full tuition remission (22%), research assistantships (21%), partial tuition remission (16%), personal loans (10%), and administrative positions (5%) (Supplementary Table S2.3).

Clinical participants reported a median unadjusted student loan debt upon graduation of $60,000 (IQR 4–130). Student loan debt appeared meaningfully higher for those earlier in their career (early *M* = 148, *SD* = 116; mid *M* = 83, *SD* = 70) and lower for those later in their career (senior *M* = 23, *SD* = 31; late senior *M* = 9, *SD* = 16). Student loan debt was notably lower for those with a PhD (*Mdn* = 30, *IQR* 0–83) and higher for those with a PsyD (*Mdn* = 122, *IQR* 70–205) (Supplementary Table S2.4).

Overall, clinical participants were relatively balanced in completing postdoctoral fellowship (54%). Postdoctoral training was somewhat more prevalent among those earlier in their career (62% early, 53% mid, 53% senior, 33% late senior) or working in private practice (75% early, 67% mid, 64% senior, 40% late senior), and particularly among those in both groups. Postdoctoral training appeared comparable for women and men across most career stages (early 56% women; mid 52%; senior 47%) except those in their late senior career (25% women) (Supplementary Table S2.1).

Less than half of clinical participants completing a postdoctoral fellowship reported that it was funded (45%). On average, those who completed a funded postdoctoral fellowship reported a median unadjusted stipend of $35,000 during this period (IQR 24–40) (Supplementary Table S2.3).

Most non-clinical participants reported their highest degree as a PhD (75%), most commonly from programs in Psychology and Law (23%) or Social Psychology (23%), among others. Among various sources of funding for graduate training, a majority of non-clinical participants reported earning graduate stipends (56%; *Mdn* = 14, *IQR* 3–18), research assistantships (53%), and full tuition remission (50%), with sizable groups also reporting teaching assistantships (41%), student loans (38%), and external employment (32%). On average, non-clinical participants reported no student loan debt upon graduation (*Mdn* = $0), though one-quarter did report owing more than $18,000 (*IQR* 0–18). Few non-clinical participants completed a postdoctoral fellowship (11%), of whom 66% reported their fellowship was funded (Supplementary Tables S2.2, S2.3).

### Professional practice

#### Licensure and board certification

Most clinical participants reported being licensed (93%), with a mean of 1.7 years (*SD* = 1.5) between their highest clinical degree and licensure. About one-third of clinical participants reported being board certified (30%), mostly through the American Board of Forensic Psychology (ABFP) (24% overall and 79% of those boarded) or more broadly through boards affiliated with the American Board of Professional Psychology (ABPP) (including ABFP; 29% overall and 94% of those boarded) (see abpp.org for more information about these boards). Those boarded through ABFP reported a mean of 10 years (*SD* = 4.9) between their highest clinical degree and board certification. Trends were noted for less time for ABFP certification among clinical participants who completed a postdoctoral fellowship (*M* = 9.0, *SD* = 5.0) compared to those who did not (*M* = 11.4, *SD* = 4.3), and among those working in institutions only (*M* = 8.3, *SD* = 4.9) or both institutions/private practice (*M* = 9.0, *SD* = 3.6) compared to private practice only (*M* = 12.1, *SD* = 5.4) (Supplementary Tables S3.1, S3.2).

#### Professional settings and practices

Clinical participants were relatively balanced among those working in institutions (37%), private practice (33%), and both (28%). On average, those working in both settings reported spending more time in institutions (*M* = 70%) vs. private practice (*M* = 30%) (both *SD* = 27.8). Most clinical participants reported working fulltime (79%) with fewer reporting part-time (11%) or fulltime with a secondary part-time position (9%). Part-time work increased with more time in the field (5% early, 10% mid, 12% senior, 30% late senior), engagement in private practice (4% institution, 11% institution/private, 19% private practice), and particularly both. Those working in institutional settings mostly reported 11–12 month contracts (85%) with fewer reporting 9–10 month contacts (11%). Most reported working about the same in 2018 as the prior year (85%), with fewer working significantly more (10%) or less (5%). Most reported only speaking English at work (94% overall; range = 92–95%). At the time of the survey (i.e., prior to the COVID-19 pandemic), clinical participants reported working most days per week in an office setting (*Mdn* = 4, *IQR* 2–5) and rarely working remotely (*Mdn* = 0, *IQR* 0–1). Those working in institutional settings reported spending more days per week in an office setting (e.g., *Mdn* = 5 only and 4 both) as compared to private practice only (*Mdn* = 3) (Supplementary Table S3.1).

Most non-clinical participants reported working full-time (82%) with fewer reporting part-time (9%) or full-time with a secondary part-time position (3%). Part-time work increased with more time in the field (8% early, 0% mid, 13% senior, 20% late senior). In institutional settings, 57% had 11–12 month contracts and 43% had 9–10 month contracts. Most reported working about the same in 2018 as the prior year (82%) with fewer working significantly more (11%) or less (6%). Nearly all reported only speaking English at work (97%). At the time of the survey (again prior to the COVID-19 pandemic), non-clinical participants reported working more days per week in an office setting (e.g., *Mdn* = 3.5) vs. remotely (*Mdn* = 1) (Supplementary Table S3.1).

#### Institutional settings

Institutional settings, departments, and titles of clinical participants are summarized in Table 2 (see additional data and notes in Supplementary Table S3.3). Most clinical participants working in institutions worked in applied settings followed by academic settings. Among applied settings, public psychiatric hospitals were the only institution where work by clinical participants appeared both common (in terms of prevalence) and regular (in terms of hours per week). Otherwise, different trends emerged based on prevalence, regularity, and work setting. For example, other relatively common institutions included court clinics, federal prisons, local jails, state prisons, forensic juvenile facilities, and primary university hospitals/academic medical centers. When considering hours worked per week, however, clinical participants appeared to work more regularly and consistently in federal prisons and public psychiatric hospitals, with similar but more variable hours per week reported in primary university hospitals/academic medical centers, forensic juvenile facilities, court clinics, state prisons, and local jails. Work with public defenders' offices was mostly reported by those also engaging in private practice. In academic settings, most reported working in 4-year universities or colleges, either with a doctoral psychology program or without.

**Table 2:** Institutional settings, departments, and titles of clinical forensic psychologists.

**Institutional setting**	**Overall**		**Professional setting**			
	**%**	**Hours per week [*****M*** **(*****SD*****)]**	**Institution only**		**Institution/private practice**	
			**%**	**Hours per week [*****M*** **(*****SD*****)]**	**%**	**Hours per week [*****M*** **(*****SD*****)]**
**Applied Institution**	77.5		80.7		73.3	
Public Psychiatric Hospital	36.4	36.0 (12.8)	39.5	37.6 (12.3)	32.2	33.4 (13.3)
Court Clinic	12.0	32.1 (23.0)	10.9	41.7 (22.8)	13.3	21.6 (18.9)
Public Defenders' Office	10.0	2.0 (0)	1.7	0.9 (0.2)	21.1	12.3 (12.7)
Federal Prison	7.2	41.6 (4.3)	9.2	42.1 (4.8)	4.4	40 (0)
Local Jail	6.7	28.2 (34.7)	5.0	7.0 (6.0)	8.9	43.4 (39.2)
State Prison	6.2	29.3 (15.5)	8.6	34.3 (15.5)	5.6	21.6 (13.9)
Forensic Juvenile Facility	4.8	34.9 (36.7)	5.9	21.1 (18.7)	3.3	67.0 (52.9)
Primary University Hospital/Academic Medical Center	1.9	40.56 (31.1)	5.0	40.56 (31.1)	0	–
**Academic Institution**	17.7		12.6		24.4	
Four-Year University/College (Non-Medical) with Doctoral Psychology Program	8.1	28.1 (15.8)	3.4	31.0 (18.3)	14.4	27.2 (15.6)
Four-Year University/College (Non-Medical) without Doctoral Psychology Program	5.3	29.7 (16.5)	5.9	33.0 (19.6)	4.4	25.5 (13.2)
	**Overall (%)**	**Professional setting (%)**	
		**Institution only**	**Institution/private practice**
**Department**			
Psychology	74.5	73.7	75.6
Criminal justice	13.0	8.5	18.9
Psychiatry	5.3	6.8	3.3
Other	9.6	8.5	2.2
**Title**			
Staff psychologist	52.9	56.8	47.8
Assistant professor	9.1	11.0	6.7
Professor	5.3	1.7	10.0
Other	25.5	24.6	26.7

Related specifically to forensic psychology activities only. Includes all eligible clinical forensic psychology participants reporting working in any institutional setting (*n* = 209). Professional Setting further includes participants working in institutional settings only (*n* = 119) or both institutional settings and private practice (*n* = 90). Actual sample sizes reduced due to item non-response. All items allowed for multiple responses. Responses with <5% response rate excluded.

Most clinical participants working in any institutional settings reported working in departments of psychology, criminal justice, or psychiatry, among others. Trends in professional setting appeared relatively balanced for psychology departments, while those working in both institutions/private practice appeared somewhat more likely to report working in criminal justice departments, and those working in institutions only appeared somewhat more likely to report working in psychiatry departments or others. The most common institutional titles were staff psychologist, assistant professor, and professor, in addition to a number of other clinical, administrative, or additional titles.

Institutional settings, departments, and titles of non-clinical participants are summarized in Table 3 (see additional data and notes in Supplementary Table S3.4). Most non-clinical participants working in institutions worked in academic settings, with fewer in applied settings. In academic settings, non-clinical participants reported working more frequently and regularly in 4-year universities or colleges, either with a doctoral psychology program or without. Less common but still regular work was reported in professional schools of psychology. In applied settings, non-clinical participants reported working in the offices of the Public Defender and District Attorney with equal frequency, but more regularity in the former. Most non-clinical participants reported working in departments of psychology followed by criminal justice or social science, along with various other departments. The most common institutional titles were associate professor or assistant professor followed by professor and lecturer/instructor, along with various other titles.

**Table 3:** Institutional settings, departments, and titles of non-clinical forensic psychologists.

**Setting**	**%**	**Hours per week [*M* (*SD*)]**
**Academic**	90.5	
Four-Year University/College (Non-Medical) with a Doctoral Psychology Program	47.6	37.7 (22.4)
Four-Year University/College (Non-Medical) without a Doctoral Psychology Program	33.3	36.8 (15.9)
Professional School of Psychology	9.5	42.5 (10.6)
**Applied**	19.0	
Public Defenders' Office	14.3	12.7 (12.0)
District Attorney's Office	14.3	2.7 (2.1)
**Department**		
Psychology	76.2	
Criminal Justice	23.8	
Social Science	9.5	
Other	9.5	
**Title**		
Associate Professor	38.1	
Assistant Professor	23.8	
Professor	14.3	
Lecturer/Instructor	9.5	
Other	23.8	

Related specifically to forensic psychology activities only. Includes all eligible non-clinical forensic psychology participants reporting working in any institutional setting (*n* = 21). Actual sample sizes reduced due to item non-response. All items allowed for multiple responses. Responses with <5% response rate excluded.

#### Private practice

Clinical participants working in private practice reported a variety of roles including outside contractor (38%), sole proprietor (28%), and partner (24%), and less frequently employee (10%). Factoring in professional setting, the most common title was outside contractor for private practice only (49%). This was followed by outside contractor, sole proprietor, or partner among those in both private practice and institutional settings (range = 27–30%), then sole proprietor or partner among those in private practice only (24 and 19%, respectively). The title of employee was less common in those working in both institutions/private practice (11%) and private practice only (8%) (Supplementary Table S3.5).

Non-clinical participants in private practice reported roles such as outside contractor (43%) followed by partner (24%) or sole proprietor (19%), and less frequently employee (5%) (Supplementary Table S3.5).

#### Professional activities

Professional activities for clinical participants are summarized in Table 4 (see additional data and notes in Supplementary Table S3.6). Forensic assessment was the only work activity that clinical participants reported was both common and regular, though even this was quite variable. Serving as an expert witness and case consultation were also common, but constituted considerably less time (albeit still quite variably). Among those engaging in expert witness activities, relatively more hours per work were reported by those working in private practice only followed by both institutions/private practice and institutions only. Otherwise, different trends emerging for other regularly reported work activities based on prevalence, time spent, and work setting.

**Table 4:** Work activities for clinical forensic psychologists.

	**Overall**		**Professional setting**					
	**%**	**Hours per week [*****M*** **(*****SD*****)]**	**Institution only**		**Institution/private practice**		**Private practice only**	
			**%**	**Hours per week [*****M*** **(*****SD*****)]**	**%**	**Hours per week [*****M*** **(*****SD*****)]**	**%**	**Hours per week [*****M*** **(*****SD*****)]**
Clinical practice—Forensic assessment	83	23.1 (20.9)	74	26.0 (20.6)	91	22.1 (23.9)	87	20.8 (18.1)
Expert Witness	71	3.2 (4.2)	57	1.7 (2.4)	71	3.3 (3.4)	89	4.3 (5.4)
Case Consultation	54	6.2 (10.6)	42	6.5 (11.7)	52	3.2 (2.4)	68	8.1 (12.9)
Supervision—Clinical Trainees	36	5.0 (4.7)	53	4.8 (4.9)	39	5.2 (4.9)	14	5.5 (3.5)
Teaching	29	7.6 (9.5)	30	8.9 (11.4)	38	8.2 (9.6)	22	4.5 (3.5)
Assessment of court functioning and administrative processes	24	14.4 (18.3)	20	12.7 (14.7)	24	17.3 (24.5)	26	13.29 (14.3)
Clinical practice—Forensic intervention	23	9.4 (11.3)	26	10.3 (12.8)	22	9.8 (12.0)	20	7.5 (7.4)
Clinical practice—General assessment	22	9.5 (10.7)	21	8.0 (6.4)	17	17.3 (24.5)	27	12.1 (14.6)
Government	16	9.7 (18.3)	15	14.6 (25.6)	20	6.6 (9.5)	14	4.0 (1.5)
Clinical practice—General intervention	16	8.6 (6.4)	19	10.7 (8.9)	11	6.9 (2.9)	16	7.2 (3.4)
Institutional Service	15	4.9 (6.5)	21	5.4 (5.9)	19	4.8 (7.9)	5	^†^
Research (clinical)	13	7.8 (6.7)	19	7.1 (5.8)	16	10.6 (8.1)	5	^†^
Training of law enforcement personnel, lawyers, judges	13	2.6 (2.6)	13	3.4 (2.9)	11	2.6 (3.1)	15	1.7 (1.3)
Supervision—Clinical and Support Personnel	12	3.9 (3.9)	17	3.7 (2.7)	14	4.8 (5.6)	6	2.4 (1.7)
Health and mental health policy	7	2.6 (2.2)	9	3.1 (2.4)	10	1.5 (1.6)	4	^†^
Research (non-clinical)	6	10.7 (19.2)	8	8.9 (6.2)	8	2.6 (2.3)	3	^†^
Fact Witness	5	2.4 (3.2)	3	^†^	3	^†^	9	2.4 (2.1)
Other	6	24.1 (55.6)	6	40.0 (78.5)	6	^†^	4	^†^
Professional Volunteering^a^		1.6 (3.6)		1.7 (4.8)		1.3 (1.8)		1.6 (2.2)

Arranged by overall prevalence (%). Includes all eligible participants in the clinical group (*n* = 323). Professional Setting further includes clinical participants working in institutional settings only (*n* = 119), both institutional settings and private practice (*n* = 90), or private practice only (*n* = 107). Actual sample sizes reduced due to item non-response. Responses with <5% response rate excluded.

^a^Prevalence not collected.

^†^Data not provided due to limited sample size.

Professional activities for non-clinical participants are summarized in Table 5 (see additional data and notes in Supplementary Table S3.7). Non-clinical participants most commonly reported engaging in non-clinical research and teaching, followed by case consultation and serving as an expert witness (the latter of which was mostly reported by those with dual non-clinical and clinical practice). Also relatively common was training of law enforcement personnel, lawyers, and judges, institutional service, and jury consultation. Notable differences were observed when considering the hours spent per week on these activities. For instance, while jury consultation was relatively less common, it constituted the highest and most variable proportion of hours per week for those professionals. A similar trend was observed for institutional service. On the other hand, some relatively more common activities constituted few hours per week, namely serving as an expert witness and training law enforcement personnel, lawyers, and judges. Otherwise, trends were more balanced for non-clinical research, teaching, and case consultation.

**Table 5:** Work activities for non-clinical forensic psychologists.

	**%**	**Hours per week [*M*(*SD*)]**
Research (non-clinical)	50.0	16.9 (21.8)
Teaching	44.1	17.9 (12.4)
Case consultation	35.3	10.1 (12.0)
Expert witness^a^	35.3	5.4 (7.1)
Training of law enforcement personnel, lawyers, judges	20.6	2.0 (1.6)
Institutional service	17.6	9.5 (11.8)
Clinical practice—Forensic assessment^a^	14.7	27.5 (8.7)
Jury consultation	14.7	27.8 (23.7)

Arranged by prevalence (%). Includes all eligible participants in the non-clinical group (*n* = 35). Actual sample sizes reduced due to item non-response. Responses with <5% response rate excluded.

^a^Generally among participants with clinical degrees (i.e., in addition to non-clinical degrees and practice).

#### Test use

Test use among clinical participants engaging in *forensic* assessment (*n* = 258) and *general* assessment (*n* = 69) is summarized in Table 6 (see additional data and notes in Supplementary Table S3.8). Among those engaging in forensic assessment, the use of *Forensic Assessment Instruments* (FAIs; i.e., measures *specifically designed* to assess psycholegal constructs or legal capacities, such as competence to stand trial) was relatively common, particularly among those in private practice. Similar rates and trends were observed for *Forensically Relevant Instruments* (FRIs) (i.e., measures that assess *forensically relevant constructs*, such as response style). The use of *objective personality tests* in forensic activities was also relatively common overall, which appeared particularly driven by their use in private practice vs. institutional settings. General use of objective personality tests appeared more common overall, particularly in private practice.

**Table 6:** Test use for clinical forensic psychologists engaging in assessment/evaluation.

	**Overall**		**Professional setting**					
			**Institution only**		**Institution/private practice**		**Private practice only**	
	**Forensic**	**General**	**Forensic**	**General**	**Forensic**	**General**	**Forensic**	**General**
**Forensic assessment instruments**								
Always	19	–	13	–	15	–	29	–
Most of the time	23	–	20	–	27	–	24	–
About half the time	17	–	21	–	19	–	10	–
Sometimes	35	–	41	–	35	–	31	–
Never	5	–	5	–	4	–	6	–
**Forensically relevant instruments**								
Always	20	–	13	–	18	–	28	–
Most of the time	21	–	21	–	23	–	19	–
About half the time	15	–	17	–	17	–	13	–
Sometimes	36	–	39	–	37	–	32	–
Never	8	–	10	–	6	–	8	–
**Intelligence tests**								
Always	9	16	5	8	6	0	16	32
Most of the time	15	32	11	32	13	33	21	29
About half the time	10	12	7	20	8	7	15	7
Sometimes	49	22	54	24	55	27	40	18
Never	17	19	24	16	19	33	9	14
**Neuropsychological/cognitive tests**								
Always	3	8	0	4	4	0	6	15
Most of the time	10	16	5	12	10	7	16	26
About half the time	10	19	7	24	8	7	13	19
Sometimes	55	37	66	44	61	50	42	26
Never	21	19	23	16	17	36	23	15
**Objective personality tests**								
Always	17	28	8	4	18	40	25	43
Most of the time	23	44	10	44	26	40	32	43
About half the time	7	6	8	8	5	7	8	4
Sometimes	42	16	52	36	44	0	30	7
Never	12	7	21	8	8	13	6	4
**Projective personality tests**								
Always	1	3	1	0	0	0	1	7
Most of the time	3	7	0	4	4	7	6	11
About half the time	3	4	1	4	3	13	5	0
Sometimes	14	24	9	33	17	7	15	21
Never	79	62	89	58	76	73	74	61

All values presented as rounded percentage (%) of responses. Sample sizes include all eligible clinical forensic psychologists reporting engagement in forensic assessment/evaluation (“Forensic”; *n* = 258) and general assessment/evaluation (“General”; *n* = 69). Professional Setting further includes participants working in institutional settings only (Forensic *n* = 87; General *n* = 25), both institutional settings and private practice (Forensic *n* = 80; General *n* = 15), or private practice only (Forensic *n* = 89; General *n* = 28). Actual sample sizes reduced due to item non-response.

Less common was use of intelligence tests, neuropsychological/cognitive tests, and projective personality tests by clinical participants in both forensic and general assessments. Forensic use of *intelligence tests* was less common (though again appearing more common in private practice settings), whereas general use of intelligence tests appeared somewhat more common overall. Similar trends were seen in *neuropsychological/cognitive tests*, though with relatively more general use only in private practice settings. Use of *projective personality tests* was much less common overall, though again relatively more common in forensic use in private practice and general use overall.

### Career considerations

#### Income

Overall income and sources of income for clinical and non-clinical participants are summarized in Table 7 (see additional data and notes in Supplementary Tables S4.1–S4.7). In terms of income source, *forensic psychology income* was reported by nearly all participants (93% clinical and 90% non-clinical), *other psychology income* was reported by a notable subsample (38% clinical and 31% non-clinical) and *other income* was generally less common (6% clinical and 3% non-clinical). A general trend among clinical participants was noted for both higher and more variable incomes for those engaging in private practice compared to those in institutional settings.

**Table 7:** Income of forensic psychologists, overall and by income source.

	**Overall**	**Clinical**				**Non-clinical**
		**Overall**	**Professional setting**			
			**Institution only**	**Institution/private practice**	**Private practice only**	
Overall	116 [90–160]	118 [94–161]	100 [80–115]	135 [100–184]	160 [111–250]	120 [79–150]
**Source**						
Forensic psychology	100 [75–140]	100 [75–140]	86 [71–106]	130 [96–165]	117 [69–200]	87 [37–115]
Other psychology	41 [16–79]	41 [15–79]	25 [10–72]	35 [12–62]	62 [25–100]	43 [31–88]
Other	19 [5–90]	15 [5–68]	20 [5–84]	^†^	7 [5–51]	^†^

All values presented as *Mdn [interquartile range]* in thousands of USD rounded from participants' pooled responses. Sample sizes include all eligible participants who reported working at least fulltime (i.e., 35 h per week or more) in the overall (*n* = 346), clinical (*n* = 280), and non-clinical samples (*n* = 29). Professional Setting further includes clinical participants working in institutional settings only (*n* = 113), both institutional settings and private practice (*n* = 80), or private practice only (*n* = 86). Actual sample sizes reduced due to item non-response.

^†^Data not provided due to limited sample size.

Overall income of clinical and non-clinical participants by career stage, degree type, postdoctoral fellowship, and board certification are summarized in Table 8 (see also Supplementary Tables S4.2–S4.5). A general trend was noted for higher and more variable incomes reported across career stages, with a particularly large increase between early career and mid-career stages. Clinical participants otherwise generally reported comparable incomes across of degree types and completion of postdoctoral fellowship. Clinical participants reported higher and more variable incomes with ABFP certification (similar trends were noted for clinical participants with any ABPP certification and any board certification in general, though again this appeared primarily driven by those with ABFP certification who composed the majority of both groups). Again, a general trend was noted across each of these considerations for higher and more variable incomes among clinical participants engaging in private practice compared to those in institutional settings.

**Table 8:** Overall income of forensic psychologists by career stage, clinical degree, postdoctoral fellowship, and board certification.

	**Overall**	**Clinical**				**Non-clinical**
		**Overall**	**Professional setting**			
			**Institution only**	**Institution/private practice**	**Private practice only**	
**Career stage**						
Early	94 [76–114]	95 [77–115]	65 [75–105]	110 [90–140]	110 [79–163]	70 [59–95]
Mid	113 [109–177]	135 [110–180]	114 [100–135]	135 [110–196]	190 [126–315]	125 [104–150]
Senior	160 [128–198]	155 [118–190]	107 [96–127]	160 [140–195]	160 [128–225]	163 [126–263]
Late senior	190 [130–350]	185 [130–350]	^†^	176 [120–225]	247 [145–360]	^†^
**Clinical degree**						
PhD		128 [95–171]	100 [85–130]	133 [100–178]	166 [118–255]	
PsyD		114 [94–150]	101 [77–112]	140 [114–202]	140 [103–238]	
**Postdoctoral fellowship**						
Yes	111 [90–172]	111 [90–173]	95 [79–110]	150 [130–200]	156 [107–256]	^†^
No	120 [90–150]	120 [95–150]	103 [81–130]	120 [99–164]	162 [125–250]	104 [71–150]
**Board certification**						
None		110 [85–140]	94 [77–114]	130 [100–160]	145 [101–180]	
Any		150 [113–220]	113 [102–130]	155 [118–196]	210 [135–340]	
ABPP (any)		149 [112–20]	113 [102–130]	155 [118–196]	200 [135–345]	
ABFP		144 [110–203]	110 [101–118]	148 [110–195]	210 [150–350]	

All values presented as *Mdn [interquartile range]* in thousands of USD rounded from participants' pooled responses. Sample sizes include all eligible participants who reported working at least fulltime (i.e., 35 h per week or more) in the overall (*n* = 346), clinical (*n* = 280), and non-clinical samples (*n* = 29). Professional Setting further includes clinical participants working in institutional settings only (n = 113), both institutional settings and private practice (*n* = 80), or private practice only (n = 86). Actual sample sizes reduced due to item non-response. Early Career = first 10 years since highest degree (first such degree if multiple); Mid-Career = 11–20 years; Senior Career = 21–30 years; Late-Senior Career = 31–50 years. ABFP, American Board of Forensic Psychology; ABPP, American Board of Professional Psychology.

^†^Data not provided due to limited sample size.

Overall income of clinical and non-clinical participants by current U.S. region and area of residence are summarized in Table 9 (see also Supplementary Table S4.6). In general, while considerable variability was observed in overall income across all regions, higher incomes were noted in certain regions (esp. Pacific and West South Central for clinical participants) with otherwise comparable incomes across the other regions. Another trend was noted in higher incomes reported by both clinical and non-clinical participants in large metropolitan and metropolitan areas, and otherwise comparable incomes across the other areas. Also observed was a continued trend for relatively higher and more variable incomes among clinical participants engaging in private practice across regions and areas.

**Table 9:** Overall income of forensic psychologists by current U.S. region and area of residence.

	**Overall**	**Clinical**				**Non-clinical**
		**Overall**	**Professional setting**			
			**Institution only**	**Institution/private practice**	**Private practice only**	
**Region**						
New England	120 [93–179]	120 [93–179]	^†^	135 [88–183]	135 [98–226]	^†^
Middle Atlantic	107 [91–150]	110 [92–150]	92 [84–102]	144 [105–169]	150 [79–198]	95 [70–199]
South Atlantic	115 [85–140]	113 [85–139]	85 [72–106]	133 [96–137]	135 [110–288]	^†^
East North Central	112 [81–191]	115 [85–191]	98 [78–117]	202 [93–223]	156 [100–450]	94 [50–327]
East South Central	128 [78–204]	128 [78–204]	^†^	^†^	^†^	^†^
West North Central	108 [80–140]	107 [80–140]	96 [72–108]	^†^	134 [120–214]	^†^
West South Central	130 [110–170]	130 [110–170]	121 [91–140]	135 [105–165]	170 [95–390]	^†^
Mountain	115 [83–150]	115 [84–157]	^†^	115 [96–162]	^†^	^†^
Pacific	130 [100–172]	130 [100–172]	108 [89–136]	130 [101–180]	166 [130–249]	^†^
**Area** ^a^						
Large Metropolitan	135 [105–180]	135 [103–180]	100 [79–114]	140 [114–190]	180 [136–324]	183 [139–321]
Metropolitan	130 [90–176]	133 [91–178]	102 [73–148]	135 [98–189]	160 [115–275]	125 [83–145]
Medium-Sized Urban	112 [91–155]	115 [94–159]	95 [81–112]	135 [101–180]	163 [113–323]	92 [60–136]
Small Urban	110 [85–138]	110 [88–134]	100 [82–115]	130 [110–195]	135 [108–195]	100 [59–150]
Rural	108 [85–140]	114 [90–146]	104 [85–128]	130 [91–161]	144 [93–211]	83 [62–108]

All values presented as *Mdn [interquartile range]* in thousands of USD rounded from participants' pooled responses. Sample sizes include all eligible participants who reported working at least fulltime (i.e., 35 h per week or more) in the overall (*n* = 346), clinical (*n* = 280), and non-clinical samples (*n* = 29). Professional Setting further includes clinical participants working in institutional settings only (*n* = 113), both institutional settings and private practice (*n* = 80), or private practice only (*n* = 86). Actual sample sizes reduced due to item non-response.

^a^Multiple responses allowed.

^†^Data not provided due to limited sample size.

Incomes and related disparities of clinical and non-clinical participants by gender are summarized in Table 10 (see also Supplementary Table S4.7). The disparity for both clinical and non-clinical participants in overall income was 0.66, meaning that on average women reported earning $0.66 for every $1.00 earned by men in the field of forensic psychology. Among clinical participants, this disparity was higher for those in private practice, compared to relative parity in institutions only. For sources of income, similar trends were observed for *forensic psychology income* across groups, and were particularly pronounced among non-clinical participants. Otherwise, differing trends were noted among clinical participants outside of forensic practice.

**Table 10:** Incomes and related disparities of forensic psychologists by gender.

	**Overall**	**Clinical**				**Non-clinical**
		**Overall**	**Professional setting**			
			**Institution only**	**Institution/private practice**	**Private practice only**	
**Overall**						
Women	106 [85–135]	106 [86–135]	100 [81–114]	130 [100–149]	130 [100–172]	108 [79–143]
Men	160 [115–241]	160 [116–244]	101 [84–140]	175 [125–218]	223 [150–350]	163 [69–310]
Disparity	0.66	0.66	0.99	0.74	0.58	0.66
**Forensic psychology**						
Women	94 [70–110]	95 [73–113]	86 [70–105]	103 [94–138]	90 [35–135]	70 [35–99]
Men	135 [90–200]	135 [91–201]	91 [75–136]	160 [110–211]	150 [95–280]	150 [59–190]
Disparity	0.70	0.70	0.95	0.64	0.60	0.47
**Other psychology**						
Women	50 [17–82]	50 [17–82]	30 [6–78]	34 [7–70]	62 [37–90]	46 [38–96]
Men	41 [13–75]	41 [13–75]	15 [10–43]	41 [16–59]	63 [22–120]	^†^
Disparity	1.22	1.22	2.00	0.83	0.98	^†^
**Other**						
Women	55 [9–106]	20 [8–108]	20 [7–121]	^†^	^†^	^†^
Men	6 [5–20]	6 [5–20]	^†^		6 [6–34]	
Disparity	9.17	3.33	^†^	^†^	^†^	^†^

All values presented as *Mdn [interquartile range]* in thousands of USD rounded from participants' pooled responses. Sample sizes include all eligible participants who reported working at least fulltime (i.e., 35 h per week or more) in the overall (*n* = 346), clinical (*n* = 280), and non-clinical samples (*n* = 29). Professional Setting further includes clinical participants working in institutional settings only (*n* = 113), both institutional settings and private practice (*n* = 80), or private practice only (*n* = 86). Actual sample sizes reduced due to item non-response. Gender grouping based on binary gender identity variable (i.e., female or male, with additional “I prefer not to respond to this item”); results did not differ when compared to individual response options (i.e., woman/female and man/male, among other options). Disparity was determined by dividing median values for women by median values for men, and therefore represent relative differences standardized on a 1.00 scale.

^†^Data not provided due to limited sample size.

Overall income and related disparities of clinical and non-clinical participants by gender and career stage are summarized in Table 11 (see also Supplementary Table S4.8). In general, disparities in overall income were lowest for those in their early careers, comparable for those in mid-careers and senior careers, and notably higher for those in their late senior careers. Unfortunately, subanalyses were limited by small samples among clinical participants later in their careers, and non-clinical participants in general.

**Table 11:** Overall income and related disparities of forensic psychologists by gender and career stage.

	**Overall**	**Clinical**				**Non-clinical**
		**Overall**	**Professional setting**			
			**Institution only**	**Institution/private practice**	**Private practice only**	
**Early career**						
Women	94 [77–111]	95 [80–113]	88 [76–106]	101 [86–130]	100 [80–160]	74 [62–95]
Men	101 [72–150]	110 [79–150]	84 [71–98]	140 [110–160]	150 [87–226]	^†^
Disparity	0.93	0.86	1.05	0.72	0.67	^†^
**Mid–career**						
Women	130 [102–150]	130 [101–150]	114 [100–130]	135 [103–169]	153 [109–219]	125 [108–150]
Men	155 [115–221]	164 [116–224]	129 [96–162]	191 [131–231]	290 [158–513]	^†^
Disparity	0.84	0.79	0.88	0.71	0.53	^†^
**Senior career**						
Women	145 [108–159]	138 [105–156]	^†^	^†^	130 [98–160]	^†^
Men	175 [135–250]	162 [135–235]	^†^	^†^	171 [135–329]	^†^
Disparity	0.83	0.85	^†^	^†^	0.76	^†^
**Late senior career**						
Women	130 [128–278]	130 [126–152]	^†^	^†^	130 [113–378]	^†^
Men	235 [145–350]	235 [145–350]	^†^	183 [115–238]	270 [203–370]	^†^
Disparity	0.55	0.55	^†^	^†^	0.48	^†^

Values are in thousands of USD rounded from participants' pooled responses. Sample sizes include all eligible participants who reported working at least fulltime (i.e., 35 h per week or more) in the overall (*n* = 346), clinical (*n* = 280), and non-clinical samples (*n* = 29). Professional Setting further includes clinical participants working in institutional settings only (*n* = 113), both institutional settings and private practice (*n* = 80), or private practice only (*n* = 86). Actual sample sizes reduced due to item non-response. Gender grouping based on binary gender identity variable (i.e., Female or Male, with additional “I prefer not to respond to this item”); results did not differ when compared to individual response options (i.e., woman/female and man/male, among other options). Early career = first 10 years since highest degree (first such degree if multiple); Mid-career = 11–20 years; Senior career = 21–30 years; Late-senior career = 31–50 years. IQR, interquartile range. Disparity was determined by dividing median values for women by median values for men, and therefore represent relative differences standardized on a 1.00 scale.

^†^Data not provided due to limited sample size.

#### Benefits

Similar trends in benefits were reported by fulltime clinical and non-clinical participants. The most common benefits were *health insurance* (70% clinical; 86% non-clinical), *retirement plans* (68% clinical; 79% non-clinical), *dental insurance* (65% clinical; 79% non-clinical), *vision insurance* (57% clinical; 76% non-clinical), and *life insurance* (55% clinical; 66% non-clinical) for both groups. Also common (range = 27–59%) were insurance for *short-term disability* and *long-term disability, flexible spending accounts* (FSAs), *health savings accounts* (HSAs), and paid leave related to *personal medical, family medical*, and *maternity*. Less common (range = 8–14%) were *paid paternity leave* and *childcare benefits*. Among clinical participants, a somewhat expected trend was observed for more benefits reported by those working in institutions compared to private practice (Supplementary Table S4.9).

Less tangible benefits were also somewhat common. *Flexible schedules* were reported by the majority of non-clinical participants (69%) and clinical participants (53%), the latter of which appeared particularly driven by those working in institutional settings (60% only and 63% both) compared to private practice only (36%). At the time of the survey (again prior to the COVID-19 pandemic), *telecommuting* was somewhat less common among non-clinical participants (35%) and clinical participants overall (24%), with this latter trend again appearing to be particularly driven by those working in private practice only (12%) compared to institutions only (26%) or both (36%) (Supplementary Table S4.9).

#### Retirement

Clinical and non-clinical participants reported a median expected age of retirement of 70 years (IQR 65–75). Among clinical participants, expected age of retirement was relatively lower for those working in institutions only (*Mdn* = 65, *IQR* 62–70) compared to those in private practice only (*Mdn* = 70, *IQR* 66–80) and both institutions/private practice (*Mdn* = 70, *IQR* 65–75). The factors reportedly affecting the timing of expected retirement were notably similar within and between all participants, with financial status ranking as most important followed by personal health, quality of work life, and family considerations. Continued availability of relevant government benefits was consistently ranked as least important by all groups (Supplementary Table S4.10).

#### Satisfaction

Given consistent and meaningful intercorrelations among satisfaction with work activities, income, and work-life balance (Supplementary Table S5.1), *overall satisfaction* was computed by taking the arithmetic mean of these three areas. Satisfaction for clinical and non-clinical participants is summarized in Table 12 (see additional data and notes in Supplementary Table S5.2). Overall, a relatively high level of overall satisfaction was reported by both clinical participants and non-clinical participants. A trend was observed among both groups for relatively higher satisfaction for work activities and then income, followed by work-life balance (with relatively higher *dis*satisfaction noted in this latter area). No meaningful differences were observed for clinical participants across professional settings.

**Table 12:** Satisfaction of forensic psychologists, overall and for work activities, income, and work-life balance.

	**Overall**	**Clinical**				**Non-clinical**
		**Overall**	**Professional setting**			
			**Institution only**	**Institution/private practice**	**Private practice only**	
**Overall**	71 (16)	71 (16)	68 (16)	70 (14)	74 (18)	76 (15)
**Work Activities**	78 (16)	78 (17)	75 (18)	77 (14)	83 (16)	81 (12)
Completely dissatisfied	*2*	*1*	*0*	*3*	*1*	*3*
Somewhat dissatisfied	*6*	*6*	*9*	*5*	*5*	*3*
Neither satisfied/dissatisfied	*2*	*2*	*3*	*2*	*2*	*0*
Somewhat satisfied	*50*	*51*	*58*	*54*	*39*	*52*
Completely satisfied	*40*	*40*	*31*	*36*	*53*	*42*
**Income**	72 (22)	71 (23)	66 (24)	72 (20)	77 (22)	76 (19)
Completely dissatisfied	*5*	*5*	*6*	*3*	*5*	*6*
Somewhat dissatisfied	*12*	*13*	*18*	*9*	*10*	*3*
Neither satisfied/dissatisfied	*6*	*6*	*7*	*5*	*6*	*3*
Somewhat satisfied	*45*	*44*	*45*	*55*	*34*	*55*
Completely satisfied	*32*	*32*	*24*	*28*	*45*	*33*
**Work-life balance**	64 (24)	64 (24)	65 (24)	62 (22)	65 (25)	70 (24)
Completely dissatisfied	*6*	*5*	*4*	*5*	*7*	*9*
Somewhat dissatisfied	*22*	*22*	*24*	*25*	*19*	*25*
Neither satisfied/dissatisfied	*10*	*10*	*10*	*14*	*7*	*9*
Somewhat satisfied	*40*	*42*	*39*	*40*	*46*	*22*
Completely satisfied	*22*	*21*	*24*	*17*	*22*	*34*

“Overall” based on combined satisfaction variable (i.e., mean of work activities, income, work-life). All continuous data (regular font) presented as *M* (*SD*) based on a sliding scale from 0 “Completely Dissatisfied” to 100 “Completely Satisfied.” All categorical data (italicized font) presented as rounded percentage (%) of responses. Sample sizes include all eligible participants who reported working at least fulltime (i.e., 35 h per week or more) in the overall (*n* = 346), clinical (*n* = 280), and non-clinical samples (*n* = 29). Professional Setting further includes clinical participants working in institutional settings only (*n* = 113), both institutional settings and private practice (*n* = 80), or private practice only (*n* = 86). Actual sample sizes reduced due to item non-response.

Overall satisfaction for clinical and non-clinical participants by gender, career stage, clinical degree, postdoctoral fellowship, and board certification is summarized in Table 13 (see additional data and notes in Supplementary Table S5.3). Among clinical participants, men reported relatively higher satisfaction than women in private practice settings, while women and men reported comparable satisfaction in institutional settings. Across professional settings, clinical participants who were board certified through ABFP reported higher (and more consistent) satisfaction in private practice only, and relatively lower (and more variable) satisfaction in institutions only, with those working in both settings falling in between (this trend was also seen in the groups certified through any ABPP board or any board generally). Within professional settings, clinical participants in private practice board certified through ABFP (and others) reported higher satisfaction compared to those who were not, whereas those in institutions board certified through any ABPP or any other board (but not ABFP specifically) reported *lower* satisfaction compared to those who were not. Otherwise, no other meaningful differences were noted among these considerations.

**Table 13:** Overall satisfaction of forensic psychologists, by gender, career stage, clinical degree, postdoctoral fellowship, and clinical board certification.

	**Overall**	**Clinical**				**Non-clinical**
		**Overall**	**Professional setting**			
			**Institution only**	**Institution/private practice**	**Private practice only**	
**Gender**						
Women	70 (16)	69 (16)	69 (14)	68 (14)	70 (19)	76 (15)
Men	74 (17)	74 (17)	63 (19)	75 (12)	79 (16)	77 (15)
**Career stage**						
Early	69 (14)	69 (14)	70 (14)	70 (14)	68 (16)	70 (18)
Mid	71 (13)	71 (13)	67 (14)	72 (12)	75 (14)	76 (12)
Senior	70 (23)	68 (23)	^†^	61 (20)	73 (21)	78 (12)
Late senior	77 (18)	77 (18)	^†^	76 (12)	81 (18)	87 (13)
**Clinical degree**						
PhD		70 (16)	67 (16)	70 (15)	73 (18)	
PsyD		72 (15)	72 (15)	71 (14)	76 (17)	
**Postdoctoral fellowship**						
Yes	72 (15)	71 (15)	70 (14)	70 (16)	74 (16)	^†^
No	71 (17)	70 (17)	66 (17)	72 (12)	75 (20)	74 (16)
**Clinical board certification**						
None		70 (16)	69 (14)	71 (14)	71 (20)	
Any						
Yes		73 (17)	61 (21)	71 (14)	80 (12)	
No		70 (16)	69 (14)	71 (14)	71 (20)	
ABPP (any)						
Yes		73 (17)	61 (21)	71 (14)	81 (12)	
No		70 (16)	69 (14)	71 (14)	71 (20)	
ABFP						
Yes		73 (16)	62 (22)	71 (12)	82 (10)	
No		70 (16)	69 (14)	70 (15)	72 (20)	

All values based on combined satisfaction variable (i.e., mean of work activities, income, work-life) and presented as *M* (*SD*) based on a sliding scale from 0 “Completely Dissatisfied” to 100 “Completely Satisfied”. Sample sizes include all eligible participants who reported working at least fulltime (i.e., 35 h per week or more) in the overall (*n* = 346), clinical (*n* = 280), and non-clinical samples (*n* = 29). Professional Setting further includes clinical participants working in institutional settings only (*n* = 113), both institutional settings and private practice (*n* = 80), or private practice only (*n* = 86). Actual sample sizes reduced due to item non-response. Gender grouping based on binary gender identity variable (i.e., Female or Male, with additional “I prefer not to respond to this item”); results did not differ when compared to non-binary response options (i.e., individual items for Woman/Female and Man/Male, among other options). Early Career stage = first 10 years since highest degree (first such degree if multiple); Mid-Career = 11–20 years; Senior Career = 21–30 years; Late-Senior Career = 31–50 years. ABPP, American Board of Professional Psychology; ABFP, American Board of Forensic Psychology.

^†^Data not provided due to limited sample size.

#### Barriers to satisfaction

Relatively few clinical and non-clinical participants reported barriers to their satisfaction overall, though some notable trends did emerge. For example, clinical participants appeared more likely to report *other personnel at work* as a barrier to their satisfaction with work activities (39% overall), driven primarily by those in institutional settings (53% only; 48% both) compared to private practice only (18%). Clinical participants were also relatively more likely to report *other personnel at work* as a barrier to their satisfaction with work-life balance (26%) and income (22%). Otherwise, *work activities* themselves were the most reported barrier to satisfaction with work-life balance (44% clinical; 42% non-clinical) and income (23% clinical; 24% non-clinical). *Family life* was a relatively common barrier to satisfaction with work activities and work-life balance for both groups (both 24 and 21%, respectively). *Personal life* was a relatively common barrier to satisfaction with work-life balance for both groups (both 21%) but not any other area. Lastly, *physical work environment* was a relatively common barrier for clinical participants in institutional settings to satisfaction with work activities (29% only; 22% both) and work-life balance (24% only), but was otherwise not a common barrier to satisfaction in other areas for either group (Supplementary Table S5.4).

#### Perceived stress

Participants reported their perceived stress on a sliding scale from “not at all stressed” (0) to “extremely stressed” (100) in relation to their student loan debt and retirement planning. Overall, both clinical participants and non-clinical participants reported relatively low perceived stress overall. For example, these groups reported comparable levels of perceived stress relating to retirement planning (clinical *M* = 39, *SD* = 28; non-clinical *M* = 35, *SD* = 26). The relative level of perceived stress related to student loan debt was over twice as high for clinical participants (*M* = 39, *SD* = 36) compared to non-clinical participants (*M* = 15, *SD* = 27). Within clinical participants, stress related to student loan debt was higher for those whose with a PsyD (*M* = 51, *SD* = 36) compared to a PhD (*M* = 32, *SD* = 35). These trends appear consistent with the relative difference in student debt reported by these groups presented and discussed above (Supplementary Table S5.5).

#### Satisfaction with survey instrument

Participants reported their satisfaction with the current survey instrument using a five-point Likert scale ranging from “completely dissatisfied” (1) to “completely satisfied” (5), with an additional “No Opinion” option (see Supplementary Table S6.1). Most reported overall satisfaction with the survey (i.e., 27% “completely satisfied” and 50% “somewhat satisfied”) with relatively consistent responses for clinical and non-clinical participants. A notable minority reported ambivalence (18% “neither satisfied nor dissatisfied” and 2% “no opinion”). Very few reported dissatisfaction (<1% “completely dissatisfied” and 3% “somewhat dissatisfied”). Additionally, open-ended feedback about recommended changes were reviewed for common themes and key points (see Supplementary Table S6.2).

## Discussion

To our knowledge, the current study is the most comprehensive field survey of forensic psychologists ever conducted. The sample of 323 clinical forensic psychologists makes this among the largest field surveys of this group to date, and enabled deeper analysis of those working in institutions, private practice, or both. Further, the sample of 35 non-clinical forensic psychologists makes this among the largest field surveys of this group to date. These professionals provided rich information about their demographics and lived experience, past training and related financial considerations, current professional practices, and various career considerations including income, benefits, retirement, and satisfaction. As such, this study offers valuable data and discussion that can benefit prospective trainees, current trainees, professionals, and field leaders alike by providing insights into the realities of practicing forensic psychology, informing decisions about training and career development, and offering guidance for advancing the field as a whole.

Key insights, relevant comparisons to prior field surveys, and related discussions are provided below. When reviewing this information, prospective and current trainees are encouraged to consider the described pathways into the field, common practices, and related trends in income and satisfaction when making decisions throughout their graduate and postdoctoral training. For example, knowledge of potential income ranges in one's early career and beyond can help inform decisions around admissions offers, student loans, and external employment during training, particularly as they relate to potential debt and resulting limitations on their career and life considerations afterward. Knowing where and how money and satisfaction are made in the field can further support students' decision-making throughout their training and early careers. Knowing how much money is made, by whom, and where can support those entering the field when considering job offers or during contract negotiations.

Data around common practices and related income, satisfaction, and other career considerations can also be critical for current professionals at any stage of their career to better assess their current standing, navigate career decisions, and pursue their professional and personal goals. For example, clinicians currently in institutional settings and considering a shift to (more) private practice work can use the presented data to better consider, plan for, and succeed in this transition. Professionals anticipating promotion and retention offers can use the data to weigh possible options elsewhere, and negotiate with their current employers to better meet their professional and personal needs. Those experiencing dissatisfaction at work can peek into the professional lives of others throughout the data and discussion shared here, to gain clarity about their current situation and develop possible solutions to improve their work life and wellbeing.

Field leaders are also encouraged to consider ways to leverage the insights presented here to strengthen forensic psychology and its workforce. For example, instructors and training directors can use the data to advise students and trainees about the field and their potential roles within it. Employers can use the data to inform hiring, retention, and other workplace strategies that best support their employees and the needs of the forensic psychology workforce. Researchers and professional organizations can use the methods and results when designing future field surveys that address important considerations among forensic psychologists. Leaders in professional organizations can use the data to inform policies and programs that meet the needs of the current field, and develop the field and practice of forensic psychology for the future.

### Demographics and lived experiences

Forensic psychologists in the current sample were generally middle-aged (e.g., *M* = 49.0 years clinical and 50.4 years non-clinical). This was generally consistent with the most relevant estimates of U.S. psychologists from 2016 (*M* = 50.0 years; APA, 2018, Figure 4). However, clinical forensic psychologists in the current sample averaged around 3 years younger than those surveyed in 2021 by Neal and Line (i.e., M = 52.8 years; 2022), and appeared to have ~5 years less professional experience (*M* = 20.26 “years of forensic evaluation experience”; versus *M* = 15.7 “years in the field” clinical and 18.4 years non-clinical for the current sample).

Among clinical forensic psychologists in the current sample, institutional practice appeared most common in their early careers (i.e., first 10 years in the field) and also mid-careers (11–20 years), and much less common in their senior careers (21–30 years) and late senior careers (31–50 years). Working in both institutions/private practice appeared comparably higher for those in their early and mid-career stages and lower for those later in their careers. Working in private practice appeared equally prevalent across all career stages. Together, these trends suggest earlier engagement in institutional practice among clinical forensic psychologists that may begin to shift to more blended institutional/private work after around 10 years (perhaps coinciding with board certification, other career advancements, or the effect of perceived barriers to satisfaction), and a generally stable clinical workforce engaging in private work. Future research may consider capturing practitioners' individual career trajectories in these settings to get a better sense of meaningful workforce changes over time at the individual and field levels.

Several trends for gender identity were noted between the current sample and prior field surveys. Overall, forensic psychologists in the current sample were predominantly female (64% cisgender women and 34% cisgender men), which again was generally consistent with the most relevant estimates of U.S. psychologists from 2016 (65% women and 35% men; APA, 2018, Table 5). The current sample's gender composition was more balanced than a recent survey of early-career forensic psychologists (83% women and 27% men; Cantone et al., 2019) and less balanced than a recent survey of clinical forensic psychologists (45.5% woman, 42.0% men, 12.5% no answer; Neal and Line, 2022).

Additionally, the current data show gender parity only among those entering the field in the last 10 years, with increasing disparity prior to this period. In contrast, Neal and Line (2022) found gender parity in their sample of clinical forensic psychologists entering the field in the late 1990s and early 2000s, with greater numbers of men entering before this time and increasing numbers of women entering afterward. This observed discrepancy cannot be solely attributed to sample differences in professional settings or years in the field, though another factor to consider may be sampling methods (e.g., professional organizations vs. licensing boards) and their impact on sample composition. Neal and Line (2022) also found differing odds of professional setting by gender, with the current data suggesting relative gender parity in private practice and more women than men working in institutional settings and combined institutions/private practice. Overall, these findings underscore the need for further research to better understand trends in gender and related disparities across time and settings, which can in turn inform efforts to promote gender equity and social justice in the field.

Regarding self-reported race/ethnicity, nearly all forensic psychologists in the current sample identified as White (~92% overall; 5% Latinx, 2% Black, and 1% Asian). These findings were consistent and somewhat more pronounced than other field surveys (i.e., 78% White, 3% Hispanic/Latino, 2% Black, and 2% Asian in Neal and Line, 2022; 81% White, 4% Black, 3% Latinx, and 2% Asian in Cantone et al., 2019) and estimates of U.S. psychologists overall (e.g., 84% White, 5% Hispanic, 4% Black/African American, and 4% Asian; APA, 2018, Table 7). Several considerations merit further discussion here. First, the underrepresentation of diverse cultural identities among forensic psychologists, particularly in studies like the current one, raises important considerations and limitations in our understanding of the field. One key issue is the lack of clarity regarding whether the reported rates truly reflect self-reported race/ethnicity of clinical and non-clinical forensic psychologists practicing in the US or affiliated with relevant professional organizations. To address this issue with clinical forensic psychologists, future field surveys may need to reconsider their sampling procedures and prioritize licensing boards rather than (or in addition to) professional organizations, which may provide a more representative sample of the clinical forensic psychology workforce. Another consideration here is the longstanding and systematic lack of data available on race, ethnicity, and other cultural identities in the field. Professional organizations like the American Psychology-Law Society and the American Academy of Forensic Psychology should systematically track data on these and related considerations among their members to better understand and address issues of representation. The problems with representation cannot be addressed if we do not know who is or is not at the table.

Beyond representation, the limited participation of Black, Latinx, Asian, Indigenous, and other forensic psychologists of color in field studies hinders our understanding of these groups. For example, the limited participation of these groups in the current study prohibited any analysis of trends in training, practice, and career considerations by race, ethnicity, or other cultural identities. As a result, current and future professionals identifying with these groups are less able to see themselves in the data in ways that may matter to them, compounding disadvantages as they seek to enter, navigate, and lead the field. Though not always necessary, the limited participation of these groups also prohibits comparisons within and between professionals based on relevant cultural identities, hindering our ability to understand and address related systematic disparities and to support equity in the field.

Lastly, it is also important to consider the demographics and lived experiences of the forensic psychology workforce in relation to those with whom we work. For example, most people in the U.S. criminal legal and juvenile justice systems are not White, come from families and communities unlike those of most clinical and non-clinical forensic psychologists, and have experienced disruptions, barriers to resources, and other personal and structural disadvantages not common among forensic psychologists. These and other relevant discrepancies influence multicultural competencies across forensic psychology practice in general. They are also potentially relevant in other psycholegal contexts, for example when working with people with functional difficulties (i.e., no functional difficulties reported by 94% of the current sample and 95% of U.S. psychologists in 2016; APA, 2018, Table 9) or those currently or previously in the military (no military service reported by 96% of the current sample). Overall, improving diversity and representation in forensic psychology research and practice is essential for advancing equity and inclusivity in the field and ensuring that the needs of all individuals, regardless of race, ethnicity, or other cultural identity, are effectively addressed.

Where one practices is another significant decision for those entering professional practice. One common trend in the current sample was movement toward various coastal regions, plus the East North Central region for non-clinical forensic psychologists. Another common trend was movement away from rural areas and toward more urban areas, which aligns with other notable national trends such as population shifts in the U.S. workforce (e.g., Pew Research Center, 2018) and noted barriers to accessing psychological services in rural areas (e.g., National Alliance on Mental Illness, 2022).

### Training and related financial considerations

Most clinical forensic psychologists had earned PhDs with a sizable group having earned PsyDs, mostly from APA/CPA accredited programs in Clinical Psychology. Many reported taking on student loans to fund their training, with fewer reporting receiving stipends ($7,000–17,000 on average), external employment, and assistantships for teaching and research. Relatively few reported receiving tuition remission, whether full (22%) or partial (16%). Most non-clinical forensic psychologists had earned PhDs in a variety of fields such as Psychology and Law or Social Psychology, among others. Among their more common funding sources were stipends ($3,000–18,000 on average) and assistantships for research or teaching, followed by student loans and external employment. Half received full tuition remission.

Understanding debt accrued from professional training is of critical importance as it can drive individual decisions and fieldwide trends in continued training and practice. In the current sample, student loan debt appeared unequally distributed among forensic psychologists. Greater debt upon graduation was reported by clinical forensic psychologists overall, particularly among those completing their training more recently and earning a PsyD. Conversely, student loan debt was uncommon for non-clinical forensic psychologists. These amounts and trends appear largely consistent with prior surveys for early career forensic psychologists (Cantone et al., 2019) and U.S. psychologists broadly (Doran et al., 2016).

Postdoctoral training is a significant decision for forensic psychology trainees. Clinical forensic psychologists in the sample were relatively split overall, with postdoctoral training somewhat more prevalent among those earlier in their careers (consistent with Cantone et al., 2019) and in private practice. Men and women appeared relatively balanced, in contrast with prior research (e.g., 43% of women vs. 16% of men in Neal and Line, 2022). Postdoctoral training was not common for non-clinical forensic psychologists.

### Professional practice

Licensure appears to be the norm among clinical forensic psychologists in the current sample and tended to happen ~2 years after entry into the field. Less common is board certification. In the current sample, ~30% of clinical forensic psychologists were board certified overall, mostly through ABPP-affiliated boards in general (29% overall) and ABFP in particular (24% overall). This rate of ABFP certification is consistent with prior research (e.g., 26.7% in Neal and Line, 2022), supporting comparisons of clinical forensic psychologists across these two samples. However, these rates of board certification are much higher than seen in the field at large, limiting relevant comparisons between the current sample and field-level data. For example, an estimated 4% of psychologists were certified through any ABPP-affiliated board in 2023, of whom 7% were certified through ABFP (representing <1% psychologists nationally; APA, 2024). In the current sample, ABFP certification tended to happen ~10 years after entry into the field, with shorter periods seen for clinical forensic psychologists who completed postdoctoral fellowships or worked in institutional settings.

Clinical forensic psychologists in the current sample were relatively balanced among those working in institutions, private practice, or both. Those working in both reported spending more than twice the amount of time in institutions than private practice. This is somewhat different from prior research (Neal and Line, 2022) where relatively more clinical forensic psychologists were in private practice (47.6 vs. 37%), a comparable number were in institutions (30.6 vs. 33%), and fewer were in both (<8 vs. 28%). Taken together, it appears common for clinical forensic psychologists to work in private practice or institutional settings, with those working in both tending to be mostly in institutional settings with private work on the side. These data also suggest potential sample bias depending on whether recruitment occurred through professional organizations vs. state licensing boards, which should be considered in interpreting the available data and also when designing future field surveys.

Clinical forensic psychologists in institutions mostly worked in applied settings, especially public psychiatric hospitals and federal prisons among numerous others. Less common were clinical forensic psychologists working in academic settings, most of whom were in 4-year universities/colleges. Non-clinical forensic psychologists were predominantly in academic institutions, especially 4-year universities/colleges, with far fewer in applied settings. Participants most commonly reported working in departments of psychology, followed by criminal justice (especially non-clinical forensic psychologists). Outside contractor was the most common role for those in private practice.

In terms of professional activities, clinical forensic psychologists reported forensic assessment as both common and constituting a significant amount of their professional time. Serving as an expert witness and case consultation were also common but less frequent. Otherwise, clinical forensic psychologists appeared meaningfully engaged in various other professional activities with differing prevalence and frequency. Non-clinical forensic psychologists reported research, teaching, and case consultation as both common and frequent, and serving as an expert witness as common but less frequent. Fewer reported engaging in jury consultation, but those that did reported that it constituted most of their professional time.

Clinical forensic psychologists engaging in forensic assessment reported using specialized forensic instruments (including both FAIs and FRIs) with some regularity, particularly in private practice settings but also in institutions. Objective personality tests were similarly used in forensic assessment, particularly in private practice though much less in institutions, and were notable more common in general assessment across settings. Intelligence tests and neuropsychological/cognitive tests were somewhat less common, particularly in forensic assessment though with more regularity noted in general practice. Lastly, projective personality tests were much less common overall. Though not fully comparable, these trends appear relatively aligned with prior research showing more regular use of objective personality tests, less regular use of intelligence tests and neuropsychological tests, and irregular use of projective personality tests among clinical forensic psychologists (Borum and Grisso, 1995).

### Career considerations

Overall incomes were relatively similar for clinical forensic psychologists (e.g., *Mdn* = $118,000) and non-clinical forensic psychologists ($120,000) in the current sample. Income from forensic psychology activities was common, whereas only about one-third of both clinical and non-clinical forensic psychologists reported income from other psychology activities. Income from any other source outside of psychology was rare.

Overall incomes for both clinical and non-clinical forensic psychologists were considerably higher than the most recent estimates for U.S. psychologists in 2015 (APA, 2017), including psychologists in general ($85,000; p. 3) and those in clinical psychology specifically ($80,000; Figure 3). Additional income comparisons can be made for clinical forensic psychologists. For example, the overall income reported by clinical forensic psychologists in the current sample appeared somewhat lower compared to prior research (*Mdn* = $125,000–149,999; Neal and Line, 2022). While both studies had similar sampling periods (i.e., 2018 and 2019, respectively), the latter sample reported having more years in the field and a greater proportion in private practice. Similar trends were observed for income by professional setting, although with the median annual salaries appearing both higher and also less variable across settings in Neal and Line (2022; i.e., private practice $175,000–199,999, applied institutions $125,000–149,000, academic institutions $100,000–124,999, “more than one setting” $150,000–174,999). The current trends also appear lower compared to a survey of the allied field of clinical neuropsychology in 2020 (Sweet et al., 2021), with higher incomes observed across professional settings (e.g., *Mdn* = $118,000 institution only; $170,000 private practice only; $170,000 both; Table 22) and for those engaging in forensic work (*M* ~ $210,000; Figure 7), but not necessarily for those who did not engage in forensic work (*M* ~ $130,000). These differences are likely attributable to meaningful differences in training and practice for these specializations, rather than methodological differences between the surveys.

In general, both clinical and non-clinical forensic psychologists reported higher and more variable overall income across each career stage, with a particularly notable increase between early career and mid-career stages. These data include only those working at least full-time, and therefore do not simply reflect trends among those who increasingly work part-time across career stages as discussed earlier. For clinical forensic psychologists, different trends were noted within career stages by professional setting, with relatively higher incomes reported by *early career* clinical forensic psychologists in private practice or both institutions/private practice as compared to those working only in institutions, though again with considerably higher variability related to increased time in private practice. This overall trend for private practice was also observed for *mid-career* clinical FP, though this time with relatively higher and more variable income seen across those in institutions only, both institution/private practice, and private practice only. Similar trends were seen among those in their senior and late-senior careers.

For clinical forensic psychologists, a general trend was seen for higher overall income with increased engagement in private practice, including in the areas noted above but also across most other considerations as well. Interestingly, overall income for clinical forensic psychologists appeared comparable regardless of degree type and postdoctoral fellowship. However, since postdoctoral fellowship can shorten the timeline for board certification, it is possible that those completing postdoctoral fellowships may benefit from increased income following board certification earlier than those who do not. Additionally, though those with a PhD and PsyD reported comparable incomes, these groups also reported drastically different levels of student loan debt (see above). Those with a PhD therefore appear to have higher *discretionary* incomes compared to those with a PsyD, likely affecting their professional and personal lives in their early careers and beyond.

Additionally, clinical forensic psychologists in the current sample reported higher but also more variable income with board certification, particularly among those working in private practice settings. For example, the median annual income for clinical forensic psychologists without any board certification was $110,000. By comparison, the median annual income for clinical forensic psychologists certified through ABFP was $144,000. When considering practice setting, the median annual income for ABFP-certified clinical forensic psychologists was $110,000 for those working in institutions only, $148,000 for those in both institution/private practice, and $210,000 in private practice only. Importantly, increased variability in overall income was also seen across these and other considerations. Again, these trends appear consistent but generally lower compared to clinical neuropsychologists (e.g., Sweet et al., 2021) when considering those with comparable board certification [e.g., American Board of Clinical Neuropsychology (ABCN); e.g., *M* = $183,800 ABCN boarded vs. $159,400 not overall; *M* = $135,300 institution only; *M* = $221,800 both; *M* = $252,400 private practice only; Tables 49, 51]. This again is likely attributable to numerous differences in these fields.

Considerable variability was observed in overall income for forensic psychologists across all U.S. regions. Overall, incomes in the current sample were considerably higher across every geographic region nationwide compared to the most recent estimates for U.S. psychologists in 2015 (APA, 2017, p. 3). Even so, relative trends in income by geographic region differed somewhat between these groups. For example, clinical forensic psychologists in the current sample reported the highest median incomes in the Pacific and West South Central regions (both $130,000) and relatively comparable incomes elsewhere ($107,000–120,000) (regional analyses for non-clinical forensic psychologists were precluded by smaller sample sizes). By comparison, the 2015 estimates of U.S. psychologists reported the highest incomes in the Middle Atlantic ($108,000), West North Central ($92,000), and West South Central ($91,000) regions, and lowest in the East South Central ($59,000) and Mountain regions ($60,000).

Higher and more variable overall incomes were reported by forensic psychologists in larger urban areas, particularly large metropolitan areas but also metropolitan areas. Lower and relatively comparable incomes were reported in medium-sized urban, small urban, and rural areas. There are of course many considerations associated with living (i.e., cost of living) and working (e.g., cost of running a private practice) in these areas, which likely have a significant effect on the discretionary income of forensic psychologists living and working in more urban vs. more rural areas.

Notable income disparity was observed between men and women in the current sample. Among both clinical and non-clinical forensic psychologists, women reported earning $0.66 for every $1.00 earned by men overall. Trends were further noted in income disparity among clinical forensic psychologists by professional setting, with greater disparity noted among those working in private practice settings alone ($0.58) or in combination with institutions ($0.74), compared to relative parity among those working only in institutional settings ($0.99). Additional trends were noted based on career stage, with less disparity noted for those in their early careers ($0.93) and comparable disparity for those in their mid-careers ($0.84) and senior careers ($0.83), particularly compared to stark disparities for those in their late senior careers ($0.55). Overall, these data suggest that income disparity between women and men in forensic psychology is greater than national estimates from the same year ($0.81; Bureau of Labor Statistics, 2019) and comparable estimates of U.S. psychologists ($0.88; calculated from APA, 2017, Figure 5). This disparity is also greater than prior research with clinical forensic psychologists ($0.83; Neal and Line, 2022), despite notable differences between women and men in their sample likely to increase income disparity such as age, years of experience, and private practice. Together, these results provide additional support for the need for systematic efforts to support equitable pay for women in forensic psychology, both at the individual level (e.g., specific training in negotiation skills for women at all professional levels; Gruber et al., 2021) and fieldwide (e.g., advocacy by professional organizations; American Psychological Association Committee on Women in Psychology, 2017), as well as the data necessary to inform and sharpen their implementation.

Similar trends were noted by both clinical and non-clinical forensic psychologists for benefits and retirement. Among clinical forensic psychologists, more benefits were generally reported by those in institutions than private practice. Both groups generally expected to retire around age 70 (or age 65 for clinical forensic psychologists in institutions), influenced mostly by financial status then personal health, work life, and family.

Clinical and non-clinical forensic psychologists reported being quite satisfied overall, particularly with their work activities and income, and somewhat less with work-life balance. Satisfaction appeared consistent across different work settings overall. However, some distinctions emerged among clinical forensic psychologists, such as men in private practice reporting somewhat higher satisfaction than women, and those with any board certification reporting higher satisfaction in private practice and lower but more variable satisfaction in institutional settings. Of note, no meaningful differences in satisfaction were observed between clinical and non-clinical forensic psychologists, or based on career stage, degree type, or postdoctoral fellowship. However, there were some potential trends that may warrant further study with larger samples, including relatively higher overall satisfaction reported by non-clinical forensic psychologists generally and later-career clinical forensic psychologists working in private practice specifically.

Further comparisons can be made between clinical forensic psychologists (i.e., *M* = 78 for work activities, 72 for income, and 64 for work-life balance) and a survey of clinical neuropsychologists in 2020 (Sweet et al., 2021). Using a similar 0–100 scale, neuropsychologists reported a similar trend and comparable levels of satisfaction with work activities (“job satisfaction”; *M* = 80.6), income (*M* = 75.3), and work-life balance (*M* = 70.2) (p. 24). The comparable income satisfaction is particularly notable given the aforementioned differences in income between these groups.

While satisfaction levels were generally high, there were still areas identified for improvement. Clinical forensic psychologists in institutional settings reported other personnel at work as a barrier to satisfaction, particularly for their work activities. Work activities themselves were also commonly reported as a barrier to work-life balance and also income. Barriers in family life, personal life, and physical work environment were less common. While clinical and non-clinical forensic psychologists generally reported relatively little perceived stress related to student loan debt and retirement planning, stress related to student loan debt was notably higher among clinical forensic psychologists generally and those with a PsyD in particular.

### Limitations

There are several limitations to the current study that should be considered alongside the presented results and related discussion. The first relates to the timing of data collection, specifically that all data were collected in 2019 with income-related data collected about 2018. Major changes in professional practice and career considerations may have occurred during this time that decrease the generalizability of the results and discussion for clinical and non-clinical forensic psychologists. One clear example here is the COVID-19 pandemic beginning in early 2020 and the meaningful effects it had on our society, field, and professional practices. It is expected that the presented data and related discussion would not reflect nor apply as well to professional practice, work activities, and career considerations over the past several years; indeed, this consideration weighted into the authors' decision about the timing of disseminating these findings. Given the field's gradual return to a new sense of normal, the presented data and related discussion may now prove more useful to those entering, navigating, and leading the field of forensic psychology. Further, the current data provide an important baseline from which future research can extend.

Another potential limitation related to the timing of data collection is the use of unadjusted dollar amounts in reporting financial data such as training-related stipends, student loan debts, and 2018 incomes. The primary aim of the presented data and related discussion was to describe and understand trends within the field, specifically relative trends between and within clinical and non-clinical forensic psychologists, which can be accomplished without adjusting for effects such as inflation. Research questions that may require such adjustments such as trends in stipends, debt, and income for forensic psychologists over time are certainly worthy areas of investigation, but were considered outside of the scope of this study.

A final potential limitation related to the timing of data collection is that the presented data and related discussion may not entirely reflect the current field of forensic psychology for reasons other than those discussed above. This limitation was addressed by comparing the current findings to prior and concurrent research, to provide context and a sense of trends over time. This process suggested relative consistency over time regarding some of the findings presented here. Still, it is expected that there are some findings presented here that do not fully represent the contemporary field or practice of forensic psychology for the reasons addressed above (e.g., changes in flexible schedule and telecommuting following COVID-19) or other developments not previously addressed. Indeed, this underscores the need for more regular field surveys targeting trends in forensic psychology training, practice, and career considerations. Still, that the current study has comparably recent data and large samples to other field surveys plus considerably more comprehensive content and analysis clearly supports its use.

A separate limitation relates to the present inability to determine how well the current study sample represents the field of forensic psychology. This determination typically relies on certain statistics, such as response rates, which require reasonable estimates of a population. However, the imprecision of the current survey mirrors the imprecision of the field of forensic psychology because it is not a single entity and is not clearly represented by any single professional organization. Rather, it is composed of a diverse array of professionals affiliated with various professional organizations, and self-organized into other less formal communities. To address this diversity and aim for a large and representative sample, the current study was designed to recruit participants from multiple populations, including major professional organizations, unaffiliated listservs, and listservs from organizations providing services to the field. The reach of the recruitment messages was large (~35,550 total contacts), and does not represent a true population given the overlapping nature of the composite groups and their heterogenous makeup of forensic psychologists and professionals outside of the field. While recognizing this limitation, it is important to note that this study is comparably large or larger than other contemporary field surveys, and employed more diverse recruitment strategies than the other surveys, further supporting the use of the data and related discussion.

Despite these diverse recruitment efforts, non-clinical forensic psychologists were meaningfully underrepresented in the current study. This may be because non-clinical forensic psychologists were less interested and invested in the results of the current survey. As our limited data demonstrate, most non-clinical forensic psychologists are working in academic settings where there is more transparency and little flexibility with salaries. Additionally, non-clinical forensic psychologists may not have identified with the use of “forensic psychology” in the current study and its recruitment materials, despite this group being captured in the official definition of the field (American Psychological Association, 2013) and the inclusion criteria for the study. Nonetheless, it is less clear whether the data and discussion are representative of non-clinical forensic psychologists overall. The limited sample sizes also restricted the data that could be presented and discussed concerning this important group. Although the current study is the largest and most comprehensive field survey of non-clinical forensic psychologists, there remains much to learn about this group. Careful consideration of the design and implementation of future field surveys is required to ensure robust representation of non-clinical forensic psychologists to capture their unique perspectives and experiences. Potential strategies include partnering more closely with organizations representing non-clinical forensic psychologists throughout the development of research aims, design, recruitment strategy, and recruitment materials to increase the relevance and importance of this research to potential participants, including the use of more recognized and meaningful terms used by members of this subgroup to support their access and participation.

Lastly, the primarily descriptive nature of the current study is recognized as a limitation. For example, while the study provides valuable insights into the income and satisfaction of forensic psychologists, the absence of multivariate modeling or other advanced analyses limits the study's ability to identify specific aspects of lived experience, training, or practice that may influence income and satisfaction in the field—or to estimate one's income or satisfaction based on these determinants. It also limits our ability to more deeply understand and address disparities observed in the current data, such as underlying mechanisms driving income disparity among men and women in the field. Research endeavors are currently underway that aim to address these limitations by employing more advanced analyses and longitudinal designs. Together, these current and future efforts will enhance our understanding of the determinants of income and satisfaction in forensic psychology, providing more nuanced and actionable insights for trainees, professionals, and field leaders.

## Conclusion

This field survey offers a comprehensive snapshot of the demographics and lived experiences, professional practices, and career considerations of a large sample of clinical and non-clinical forensic psychologists in the US. The results and related discussion are valuable to prospective and current students aspiring to enter the field, current professionals seeking to build and sustain successful careers in the field, and field leaders aiming to contribute to the advancement of forensic psychology as a field and practice. Future studies can build upon this foundational work by providing regular updates to our knowledge, examining trends over time, and investigating the impact of major systemic and societal changes on the field's composition, practices, finances, and wellbeing. The overarching goal of this work is to strengthen forensic psychology by fostering a deeper understanding of those who engage in training, research, teaching, and practice in this vibrant and evolving field.

## Data Availability

The raw data supporting the conclusions of this article will be made available by the authors, without undue reservation.
