# Supplementary material for: Training, practice, and career considerations in forensic psychology: results from a field survey of clinical and non-clinical professionals in the United States
Source: Front Psychol. 2024 Nov 12;15:1439874. doi: 10.3389/fpsyg.2024.1439874 (PMC11638730; doi:10.3389/fpsyg.2024.1439874)
Supplement: Supplementary file 1 [file Table_1.DOCX]

Supplementary Material

# Supplemental Tables

**Supplemental Table 1.1**

***Demographics—Current Identities and Lived Experiences***

|  | Overall | Clinical | | | | | Non-Clinical |
| --- | --- | --- | --- | --- | --- | --- | --- |
|  |  | Overall | Professional Setting | | | |  |
|  |  |  | Institution Only | Institution/ Private Practice | Private Practice Only | Difference |  |
| Age |  |  |  |  |  |  |  |
| *Mdn* [IQR] | 46 [38-60] | 46 [38-60] | 38 [35-45.5] | 47 [40-60] | 59 [45-67] |  | 51 [38-59] |
| *M* (*SD*) | 48.9 (13.3) | 49.0 (13.3) | 41.3 (9.3) | 50.1 (12.7) | 56.6 (13.1) | F(2,293) = 45.79***, η^2^ = **0.24** | 50.4 (14.4) |
| Years in the field^a^ |  |  |  |  |  |  |  |
| *Mdn* [*IQR*] | 13 [5-23] | 12.5 [5-23] | 6 [3-13.5] | 14 [7-21] | 21.5 [11-33] |  | 17 [5-25] |
| *M* (*SD*) | 15.7 (12.4) | 15.7 (12.4) | 9.2 (8.3) | 16.4 (11.6) | 22.2 (13.3) | F(2,311) = 38.16***, η^2^ = **0.20** | 18.4 (14.5) |
| Career Stage (%)^b^ |  |  |  |  |  |  |  |
| Early Career | 41.4 | 41.5 | 64.7 | 32.6 | 23.6 | *χ^2^* (6, *N* = 314) = 70.94***, Cramer’s V = **.34** | 34.3 |
| Mid-Career | 29.6 | 29.6 | 27.7 | 40.4 | 23.6 |  | 25.7 |
| Senior Career | 13.9 | 13.2 | 4.2 | 11.2 | 23.6 |  | 22.9 |
| Late Senior Career | 15.1 | 15.7 | 3.4 | 15.7 | 29.2 |  | 14.3 |
| Gender Identity (%)^c,d^ |  |  |  |  |  |  |  |
| Woman/Female | 63.5 | 62.7 | 78.4 | 61.4 | 47.6 | *χ^2^* (2, *N* = 307) = 22.54***, Cramer’s V = .27 | 72.7 |
| Man/Male | 34.4 | 35 | 20.7 | 37.5 | 48.5 | *χ^2^* (2, *N* = 307) = 19.02***, Cramer’s V = .25 | 27.3 |
| Career Stage by Gender (Female %)^b^ |  |  |  |  |  |  |  |
| Early Career | 52.4 | 53.4 | 71.4 | 41.5 | 33.3 | *χ^2^* (3, *N* = 307) = 51.55***, Cramer’s V = **.41** | 37.5 |
| Mid-Career | 31.1 | 31.1 | 25.3 | 43.4 | 29.2 |  | 29.2 |
| Senior Career | 8.5 | 7.8 | 1.1 | 9.4 | 16.7 |  | 20.8 |
| Late Senior Career | 8.0 | 7.8 | 2.2 | 5.7 | 20.8 |  | 12.5 |
| Cultural Identity (%)^c,e^ |  |  |  |  |  |  |  |
| White/European American | 91.7 | 91.6 | 89.7 | 94.3 | 93.2 | *χ^2^* (2, *N* = 307) = 1.73, Cramer’s V = .08 | 93.9 |
| Latinx | 5.3 | 4.2 | 3.4 | 4.5 | 3.9 | † | 15.2 |
| Black  /African American | 1.8 | 1.9 | 4.3 | 1.1 | 0 | † | 0 |
| Sexual Orientation (%)^c,f^ |  |  |  |  |  |  |  |
| Heterosexual/ Straight | 87.8 | 88.4 | 93.1 | 86.4 | 85.4 | *χ^2^* (2, *N* = 307) = 3.79, Cramer’s V = .11 | 84.8 |
| Bisexual | 3.6 | 2.9 | 2.6 | 3.4 | 2.9 | † | 9.1 |
| Lesbian | 2.7 | 2.9 | 1.7 | 5.7 | 1.9 | † | 0 |
| Gay | 1.8 | 1.9 | 0 | 2.3 | 3.9 | † | 0 |
| Pansexual | 1.5 | 1.3 | 0.9 | 1.1 | 1.9 | † | 2.9 |
| Functional Difficulties (%)^c,g^ |  |  |  |  |  |  |  |
| None | 94.1 | 94.2 | 95.7 | 95.5 | 93.2 | *χ^2^* (2, *N* = 307) = 0.79, Cramer’s V = .05 | 90.9 |
| Serious Difficulty Walking or Climbing Stairs | 1.5 | 1.6 | 0.9 | 2.3 | 1.9 | † | 3.0 |
| Military Service (%)^h^ |  |  |  |  |  |  |  |
| Never Served | 95.8 | 95.8 | 96.5 | 96.6 | 95.1 | *χ^2^* (6, *N* = 306) = 3.39, Cramer’s V = .07 | 96.9 |
| Past Active Duty | 3.0 | 2.9 | 2.6 | 2.3 | 3.9 |  | 3.1 |

*Note.* Sample sizes generally include all eligible participants in the overall (*n* = 351), clinical (n = 323), and non-clinical samples (*n* = 35). Professional Setting further includes clinical participants working in institutional settings only (n = 119), both institutional settings and private practice (n = 90), or private practice only (n = 107). Actual sample sizes reduced due to item non-response. IQR = Interquartile range. **Bold** indicates significant difference based on measure of association (i.e., at least a medium effect per *η^2^* > .06 and Cramer’s V > .30; Cohen, 1988, 1992). †Data not provided due to limited sample size.

^a^ Estimated based on reported year of highest relevant degree (first degree if multiple) and date of study completion.

^b^ Based on number of years since participants’ highest degree (first used if multiple) for early career (1-10 years), mid-career (11-20 years), senior (21-30 years), and late senior career (31-40 years). Additional participants did not provide sufficient information (*n* = 6).

^c^ Multiple responses allowed.

^d^ Additional gender identities with limited representation in the sample include trans women/male-to-female (MTF) (*n* = 0), trans men/female-to-male (FTM) (*n* = 0), genderqueer/gender non-conforming/non-binary (*n* = 2), and other (*n* = 1; i.e., “Unique”). Additional participants preferred to not respond (*n* = 3) or did not provide sufficient information (*n* = 14). When asked about gender identity in a binary manner, participants identified as Female (64.2%, *n* = 213) or Male (35.8%, *n* = 119); additional participants preferred not to respond (*n* = 2) or did not provide sufficient information (*n* = 14).

^e^ Latinx includes participants who identified as Latina, Latino, Latinx, Hispanic, or any heritage from a Latin American country. Additional cultural identities with limited representation in the sample include Biracial/Multiracial (*n* = 5), Asian/Asian American/Pacific Islander (*n* = 4), Middle Eastern/Arab/Turkish/Iranian (*n* = 2), Native American/American Indian/Indigenous (*n* = 1), and Other (*n* = 3; i.e., “American,” “East Indian,” and “Portuguese”). Additional participants preferred to not respond (*n* = 5) or did not provide sufficient information (*n* = 14).

^f^ Additional sexual orientations with limited representation include Queer (*n* = 3), Asexual (*n* = 1), and Other (*n* = 1; i.e., “Heteroflexible”). Additional participants preferred to not respond (*n* = 6) or did not provide sufficient information (*n* = 14).

^g^ Additional functional difficulties with limited representation include deafness or serious difficulty hearing (*n* = 3); blindness or having serious difficulty seeing even when wearing glasses (*n* = 2); serious difficulty concentrating, remembering, or making decisions (*n* = 1); or serious difficulty dressing or bathing (*n* = 0). Additional participants preferred to not respond (*n* = 4) or did not provide sufficient information (*n* = 14).

^h^ Additional participants were currently on active duty (*n* = 1), only on active duty for training in the Reserves or National Guard (*n* = 2), preferred not to respond to this item (*n* = 1), or did not provide sufficient information (*n* = 16).

**p* < .05. ***p* < .01. ****p* < .001.

**Supplemental Table 1.2**

***Demographics—Development***

|  | Overall | Clinical | | | | | Non-Clinical |
| --- | --- | --- | --- | --- | --- | --- | --- |
|  |  |  | Professional Setting | | | |  |
|  |  | Overall | Institution Only | Institution/ Private Practice | Private Practice Only | Difference |  |
| Country of Origin (%)^a^ |  |  |  |  |  |  |  |
| United States of America | 95.2 | 95.4 | 97.3 | 94.3 | 95.1 | *χ^2^* (14, *N* = 302) = 13.05, Cramer’s V = .15 | 93.9 |
| Canada | 1.8 | 1.6 | 0.9 | 2.3 | 1.9 |  | 3.0 |
| Region of Early Development (%)^b^ |  |  |  |  |  |  |  |
| New England | 5.4 | 5.4 | 3.4 | 4.9 | 6.8 | *χ^2^* (16, *N* =222) = 9.50, Cramer’s V = .21 | 4.8 |
| Middle Atlantic | 19.5 | 20.1 | 25.0 | 11.5 | 21.9 |  | 14.3 |
| South Atlantic | 13.7 | 13.8 | 13.6 | 16.4 | 11.0 |  | 14.3 |
| East North Central | 17.8 | 18.8 | 18.2 | 23.0 | 16.4 |  | 4.8 |
| East South Central | 3.3 | 3.1 | 2.3 | 3.3 | 4.1 |  | 4.8 |
| West North Central | 11.6 | 11.6 | 11.4 | 11.5 | 12.3 |  | 9.5 |
| West South Central | 7.1 | 6.7 | 8.0 | 8.2 | 4.1 |  | 14.3 |
| Mountain | 5.8 | 5.4 | 3.4 | 8.2 | 5.5 |  | 9.5 |
| Pacific | 15.8 | 15.2 | 14.8 | 13.1 | 17.8 |  | 23.8 |
| Area of Early Development (%)^c^ |  |  |  |  |  |  |  |
| Large Metropolitan | 22.3 | 22.8 | 19.0 | 18.2 | 30.1 | *χ^2^* (2, *N* = 307) = 5.19, Cramer’s V = .13 | 21.2 |
| Metropolitan | 13.1 | 12.5 | 12.1 | 13.6 | 12.6 | *χ^2^* (2, *N* = 307) = 0.11, Cramer’s V = .02 | 14.3 |
| Medium-Sized Urban | 16 | 16.1 | 16.4 | 21.6 | 11.7 | *χ^2^* (2, *N* = 307) = 3.44, Cramer’s V = .11 | 12.1 |
| Small Urban | 22.6 | 22.8 | 23.3 | 26.1 | 19.4 | *χ^2^* (2, *N* = 307) = 1.24, Cramer’s V = .06 | 18.2 |
| Rural | 28.8 | 27.7 | 32.8 | 22.7 | 27.2 | *χ^2^* (2, *N* = 307) = 2.55, Cramer’s V = .09 | 45.5 |
| Primary Caregiver (%)^d^ |  |  |  |  |  |  |  |
| Mother | 75.2 | 76.4 | 81.4 | 77.3 | 71.3 | *χ^2^* (6, *N* = 302) = 7.42, Cramer’s V = .11 | 65.6 |
| Father | 24.2 | 23.0 | 16.8 | 22.7 | 28.7 |  | 34.4 |
| Secondary Caregiver (%)^e^ |  |  |  |  |  |  |  |
| Father | 75.0 | 75.9 | 82.1 | 74.7 | 71.7 | *χ^2^* (6, *N* = 288) = 6.29, Cramer’s V = .10 | 67.7 |
| Mother | 24.1 | 23.0 | 17.0 | 24.1 | 27.3 |  | 32.2 |
| Education—Primary Caregiver (%)^f^ |  |  |  |  |  |  |  |
| Primary/Elementary School | 1.5 | 1.6 | 2.7 | 1.1 | 1.0 | *χ^2^* (18, *N* = 302) = 21.70, Cramer’s V = .19 | 0 |
| Some High School | 3.0 | 3.0 | 1.8 | 4.5 | 3.0 |  | 3.1 |
| High School Diploma/GED | 23.6 | 23.6 | 19.5 | 26.1 | 25.7 |  | 21.9 |
| Technical/Trade School | 6.1 | 5.2 | 0.9 | 5.7 | 9.9 |  | 12.5 |
| Associate’s Degree | 5.8 | 6.2 | 8.8 | 5.7 | 3.0 |  | 3.1 |
| Some College | 7.9 | 8.2 | 9.7 | 8.0 | 6.9 |  | 6.3 |
| Bachelor’s Degree | 16.7 | 17.0 | 21.2 | 11.4 | 17.8 |  | 18.8 |
| Some Graduate/Professional School | 3.9 | 4.3 | 2.7 | 6.8 | 4.0 |  | 21.9 |
| Master’s Degree | 21.8 | 21.6 | 23.0 | 19.3 | 21.8 |  | 21.9 |
| Doctorate or Professional Degree | 9.7 | 9.2 | 9.7 | 11.4 | 6.9 |  | 12.5 |
| Education—Secondary Caregiver (%)^g^ |  |  |  |  |  |  |  |
| Primary/Elementary School | 1.6 | 1.7 | 2.8 | 0 | 1.0 | *χ^2^* (20, *N* = 288) = 22.43, Cramer’s V = .20 | 0 |
| Some High School | 5.1 | 5.2 | 5.7 | 4.8 | 5.1 |  | 3.2 |
| High School Diploma/GED | 22.5 | 22.7 | 19.8 | 22.9 | 26.3 |  | 22.6 |
| Technical/Trade School | 6.3 | 6.9 | 9.4 | 6.0 | 5.1 |  | 0 |
| Associate’s Degree | 5.1 | 4.5 | 4.7 | 7.2 | 1.0 |  | 9.7 |
| Some College | 11.1 | 11.7 | 9.4 | 14.5 | 12.1 |  | 6.5 |
| Bachelor’s Degree | 19.6 | 19.6 | 21.7 | 12.0 | 24.2 |  | 16.1 |
| Some Graduate/Professional School | 3.8 | 3.8 | 1.9 | 6.0 | 4.0 |  | 3.2 |
| Master’s Degree | 13 | 11.3 | 8.5 | 15.7 | 10.1 |  | 29.0 |
| Doctorate or Professional Degree | 11.7 | 12.4 | 16.0 | 10.8 | 10.1 |  | 9.7 |
| Familial Use of Public Assistance (%)^h^ |  |  |  |  |  |  |  |
| No | 88.6 | 88.6 | 86.0 | 87.5 | 93.1 | *χ^2^* (6, *N* = 304) = 12.46, Cramer’s V = .14 | 90.9 |
| Yes | 9.0 | 9.1 | 13.2 | 6.8 | 6.9 |  | 6.1 |

*Note.* Sample sizes generally include all eligible participants in the overall (*n* = 351), clinical (n = 323), and non-clinical samples (*n* = 35). Professional Setting further includes clinical participants working in institutional settings only (n = 119), both institutional settings and private practice (n = 90), or private practice only (n = 107), unless otherwise noted below. Actual sample sizes reduced due to item non-response. **Bold** indicates significant difference based on measure of association (i.e., at least a medium effect per *η^2^* > .06 and Cramer’s V > .30; Cohen, 1988, 1992).

^a^ Additional countries of origin with limited representation include the United Kingdom (*n* = 3), India (*n* = 2), Chile, Japan, South Africa, Spain, and Sweden (all *n* = 1). Additional participants did not provide sufficient information (*n* = 20).

^b^ Restricted to participants residing in the United States during early development and reporting a ZIP code. New England = Connecticut, Maine, Massachusetts, New Hampshire, Rhode Island and Vermont; Middle Atlantic = New Jersey, New York and Pennsylvania; South Atlantic = Delaware, District of Columbia, Florida, Georgia, Maryland, North Carolina, South Carolina, Virginia and West Virginia; East North Central = Illinois, Indiana, Michigan, Ohio and Wisconsin; East South Central = Alabama, Kentucky, Mississippi and Tennessee; West North Central = Iowa, Kansas, Minnesota, Missouri, Nebraska, North Dakota and South Dakota; West South Central = Arkansas, Louisiana, Oklahoma and Texas; Mountain = Arizona, Colorado, Idaho, Montana, Nevada, New Mexico, Utah and Wyoming; Pacific = Alaska, California, Hawaii, Oregon and Washington. Grouping is based on the primary area of early development reported by participants (viz. ZIP codes), and may not fully capture the experience of participants who lived in more than one of these and other geographical divisions. Additional participants did not provide sufficient information (*n* = 110).

^c^ Multiple responses allowed. Large Metropolitan = Population 1.5 million or more; Metropolitan = Population between 500,000 and 1.5 million; Medium-Sized Urban = Population between 200,000 and 500,000; Small Urban = Population between 50,000 and 200,000; Rural = Population 50,000 or less (Organisation for Economic Co-operation and Development, 2012). Additional participants responded Other (*n* = 17; mostly “suburban,” “town,” “remote,” or similar), preferred to not respond (*n* = 5), or did not provide sufficient information (*n* = 14).

^d^ Additional primary caregivers included Grandmother (*n* = 1) and other (*n* = 1). Additional participants did not provide sufficient information (*n* = 21).

^e^ Additional secondary caregivers included Grandmother (*n* = 1) and other (*n* = 2). Additional participants did not provide sufficient information (*n* = 35). Few participants reported tertiary (*n* = 16), quaternary (*n* = 6), or quinary caregivers (*n* = 2).

^f^ Additional participants did not provide sufficient information (*n* = 21).

^g^ Additional participants did not know (*n* = 1) or did not provide sufficient information (*n* = 35).

^h^ Additional participants did not know (*n* = 6), preferred to not respond (*n* = 2), or did not provide sufficient information (*n* = 17).

**p* < .05. ***p* < .01. ****p* < .001.

**Supplemental Table 1.3**

***Demographics—Current Residence and Related Changes***

|  | Overall | Clinical | | | | | Non-Clinical |
| --- | --- | --- | --- | --- | --- | --- | --- |
|  |  | Overall | Professional Setting | | | |  |
|  |  |  | Institution Only | Institution/ Private Practice | Private Practice Only | Difference |  |
| Current Region (%)^a^ |  |  |  |  |  |  |  |
| New England | 5.4 | 5.8 | 2.3 | 6.0 | 9.3 | *χ^2^* (16, *N* = 239) = 16.68, Cramer’s V = .19 | 14.3 |
| Middle Atlantic | 10.5 | 9.9 | 10.5 | 11.9 | 7.0 |  | 19.0 |
| South Atlantic | 21.0 | 20.7 | 20.9 | 19.4 | 20.9 |  | 19.0 |
| East North Central | 12.1 | 11.2 | 11.6 | 10.4 | 11.6 |  | 19.0 |
| East South Central | 2.3 | 2.5 | 2.3 | 0 | 4.7 |  | 0 |
| West North Central | 9.7 | 9.9 | 15.1 | 4.5 | 9.3 |  | 9.5 |
| West South Central | 5.8 | 6.2 | 5.8 | 7.5 | 5.8 |  | 4.8 |
| Mountain | 7.8 | 7.4 | 3.5 | 11.9 | 8.1 |  | 9.5 |
| Pacific | 25.3 | 26.4 | 27.9 | 28.4 | 23.3 |  | 4.8 |
| Change in Region (%)^b^ |  |  |  |  |  |  |  |
| New England | 0 | +0.4 | -1.1 | +1.1 | +2.5 |  | +9.5 |
| Middle Atlantic | -9 | -10.2 | -14.5 | +0.4 | -14.9 |  | +4.7 |
| South Atlantic | +7.3 | +6.9 | +7.3 | +3.0 | +9.9 |  | +4.7 |
| East North Central | -5.7 | -7.6 | -6.6 | -12.6 | -4.8 |  | +14.2 |
| East South Central | -1.0 | -0.6 | 0 | -3.3 | +0.6 |  | -4.8 |
| West North Central | -1.9 | -1.7 | +3.7 | -7.0 | -3.0 |  | 0 |
| West South Central | -1.3 | -0.5 | -2.2 | -0.7 | 1.7 |  | -9.5 |
| Mountain | +2.0 | +2.0 | +0.1 | +3.7 | +2.6 |  | 0 |
| Pacific | +9.5 | +11.2 | +13.1 | +15.3 | +5.5 |  | -19.0 |
| Current Area (%)^c^ |  |  |  |  |  |  |  |
| Large Metropolitan | 23.4 | 22.8 | 19.8 | 23.9 | 25.2 | *χ^2^* (2, *N* = 307) = 0.99, Cramer’s V = .06 | 30.3 |
| Metropolitan | 19.9 | 20.6 | 17.2 | 25.0 | 21.4 | *χ^2^* (2, *N* = 307) = 1.85, Cramer’s V = .08 | 12.1 |
| Medium-Sized Urban | 16.9 | 17.0 | 16.4 | 19.3 | 15.5 | *χ^2^* (2, *N* = 307) = 0.54, Cramer’s V = .04 | 12.1 |
| Small Urban | 23.7 | 23.8 | 29.3 | 15.9 | 24.3 | *χ^2^* (2, *N* = 307) = 4.98, Cramer’s V = .13 | 21.2 |
| Rural | 15.1 | 14.5 | 16.4 | 12.5 | 14.6 | *χ^2^* (2, *N* = 307) = 0.60, Cramer’s V = .04 | 27.3 |
| Change in Area (%)^d^ |  |  |  |  |  |  |  |
| Large Metropolitan | +1.1 | 0 | +0.8 | +5.7 | -4.9 |  | +9.1 |
| Metropolitan | +6.8 | +8.1 | +5.1 | +11.4 | +8.8 |  | -2.2 |
| Medium-Sized Urban | +0.9 | +0.9 | +0.0 | -2.3 | +3.8 |  | 0 |
| Small Urban | +1.1 | +1.0 | +6.0 | -10.2 | +4.9 |  | +3.0 |
| Rural | -13.7 | -13.2 | -16.4 | -10.2 | -12.6 |  | -18.2 |

*Note.* Sample sizes generally include all eligible participants in the overall (*n* = 351), clinical (n = 323), and non-clinical samples (*n* = 35). Professional Setting further includes clinical participants working in institutional settings only (n = 119), both institutional settings and private practice (n = 90), or private practice only (n = 107). Actual sample sizes reduced due to item non-response. **Bold** indicates significant difference based on measure of association (i.e., at least a medium effect per Cramer’s V > .30; Cohen, 1988, 1992).

^a^ New England = Connecticut, Maine, Massachusetts, New Hampshire, Rhode Island and Vermont; Middle Atlantic = New Jersey, New York and Pennsylvania; South Atlantic = Delaware, District of Columbia, Florida, Georgia, Maryland, North Carolina, South Carolina, Virginia and West Virginia; East North Central = Illinois, Indiana, Michigan, Ohio and Wisconsin; East South Central = Alabama, Kentucky, Mississippi and Tennessee; West North Central = Iowa, Kansas, Minnesota, Missouri, Nebraska, North Dakota and South Dakota; West South Central = Arkansas, Louisiana, Oklahoma and Texas; Mountain = Arizona, Colorado, Idaho, Montana, Nevada, New Mexico, Utah and Wyoming; Pacific = Alaska, California, Hawaii, Oregon and Washington. Grouping is based on the primary area of current residence reported by participants (viz. ZIP codes) and appears to capture the experience of most participants given the limited number who lived across multiple geographical divisions. Additional participants did not provide sufficient information (*n* = 94).

^b^ Calculated by subtracting percent of Region of Early Development (see Supplemental Table 1.2) from Current Region (current).

^c^ Multiple responses allowed. Large Metropolitan = Population 1.5 million or more; Metropolitan = Population between 500,000 and 1.5 million; Medium-Sized Urban = Population between 200,000 and 500,000; Small Urban = Population between 50,000 and 200,000; Rural = Population 50,000 or less (Organisation for Economic Co-operation and Development, 2012). Additional participants responded Other (*n* = 4), preferred to not respond (*n* = 2), or did not provide sufficient information (*n* = 14).

^d^ Calculated by subtracting percent of Area of Early Development (see Supplemental Table 1.2) from Current Area (current).

**p* < .05. ***p* < .01. ****p* < .001.

**Supplemental Table 1.4**

***Demographics—Current Residence and Related Changes, By Region and Area, for Overall Sample***

|  | Area of Early Development^b^ (%) | | | | | |
| --- | --- | --- | --- | --- | --- | --- |
|  | Large Metropolitan | Metropolitan | Medium-Sized Urban | Small  Urban | Rural | Total |
| Region of Early Development^a^ |  |  |  |  |  |  |
| New England | 0.4 | 0.4 | 0.4 | 1.2 | 2.9 | 5.4 |
| Middle Atlantic | 7.5 | 1.2 | 1.7 | 2.9 | 5.4 | 18.7 |
| South Atlantic | 3.7 | 2.1 | 2.5 | 4.6 | 2.9 | 15.8 |
| East North Central | 4.6 | 0.8 | 2.5 | 6.2 | 4.6 | 18.7 |
| East South Central | 0.0 | 0.4 | 0.8 | 0.4 | 1.7 | 3.3 |
| West North Central | 0.0 | 1.7 | 2.1 | 2.9 | 6.2 | 12.9 |
| West South Central | 1.2 | 1.2 | 1.7 | 2.1 | 1.7 | 7.9 |
| Mountain | 0.8 | 1.2 | 0.8 | 0.4 | 2.5 | 5.8 |
| Pacific | 5.0 | 1.2 | 3.7 | 2.9 | 3.3 | 16.2 |
| Total | 23.2 | 10.4 | 16.2 | 23.7 | 31.1 | - |
|  | Current Area^b^ (%) | | | | | |
|  | Large Metropolitan | Metropolitan | Medium-Sized Urban | Small  Urban | Rural | Total |
| Current Region^a^ |  |  |  |  |  |  |
| New England | 1.9 | 0.0 | 0.8 | 2.3 | 1.2 | 6.2 |
| Middle Atlantic | 4.3 | 0.4 | 1.2 | 1.6 | 3.1 | 10.5 |
| South Atlantic | 4.3 | 2.7 | 3.5 | 5.8 | 6.6 | 23.0 |
| East North Central | 3.9 | 1.6 | 1.6 | 3.1 | 2.7 | 12.8 |
| East South Central | 0.4 | 0.4 | 0.8 | 0.0 | 0.8 | 2.3 |
| West North Central | 1.6 | 2.3 | 1.9 | 1.9 | 3.1 | 10.9 |
| West South Central | 1.2 | 0.0 | 0.8 | 1.6 | 3.1 | 6.6 |
| Mountain | 1.2 | 1.6 | 2.3 | 1.6 | 1.9 | 8.6 |
| Pacific | 7.0 | 2.3 | 3.5 | 4.7 | 6.6 | 24.1 |
| Total | 25.7 | 11.3 | 16.3 | 22.6 | 29.2 | - |
|  | Change in Area^b,d^ (%) | | | | | |
|  | Large Metropolitan | Metropolitan | Medium-Sized Urban | Small  Urban | Rural | Total |
| Change in Region^a,c^ |  |  |  |  |  |  |
| New England | +1.5 | -0.4 | +0.4 | +1.1 | -1.7 | +0.8 |
| Middle Atlantic | -3.2 | -0.9 | -0.5 | -1.3 | -2.3 | -8.2 |
| South Atlantic | +0.5 | +0.6 | +1.0 | +1.3 | +3.7 | +7.2 |
| East North Central | -0.7 | +0.7 | -0.9 | -3.1 | -1.8 | -5.8 |
| East South Central | +0.4 | 0 | -0.1 | -0.4 | -0.9 | -1.0 |
| West North Central | +1.6 | +0.7 | -0.1 | -1.0 | -3.1 | -2.0 |
| West South Central | -0.1 | -1.2 | -0.9 | -0.5 | +1.5 | -1.3 |
| Mountain | +0.3 | +0.3 | +1.5 | +1.1 | -0.5 | +2.8 |
| Pacific | +2.0 | +1.1 | -0.2 | +1.8 | +3.3 | +7.9 |
| Total | +2.4 | +0.9 | +0.2 | -1.1 | -1.9 | - |

*Note.* Valid percentages based on all eligible participants in the overall sample who provided responses for region and area of early development (*n* = 241) and current region and area (*n* = 257); subgroup analyses are not provided due to relevance and sample size limitations. Values for Total differ from Supplemental Table 1.3 due to differences in sample sizes for this multivariate analysis.

^a^ Grouping based on the primary region of residence reported by participants (viz. ZIP codes). New England = Connecticut, Maine, Massachusetts, New Hampshire, Rhode Island and Vermont; Middle Atlantic = New Jersey, New York and Pennsylvania; South Atlantic = Delaware, District of Columbia, Florida, Georgia, Maryland, North Carolina, South Carolina, Virginia and West Virginia; East North Central = Illinois, Indiana, Michigan, Ohio and Wisconsin; East South Central = Alabama, Kentucky, Mississippi and Tennessee; West North Central = Iowa, Kansas, Minnesota, Missouri, Nebraska, North Dakota and South Dakota; West South Central = Arkansas, Louisiana, Oklahoma and Texas; Mountain = Arizona, Colorado, Idaho, Montana, Nevada, New Mexico, Utah and Wyoming; Pacific = Alaska, California, Hawaii, Oregon and Washington.

^b^ Values do not equal 100 because multiple responses allowed. Large Metropolitan = Population 1.5 million or more; Metropolitan = Population between 500,000 and 1.5 million; Medium-Sized Urban = Population between 200,000 and 500,000; Small Urban = Population between 50,000 and 200,000; Rural = Population 50,000 or less (Organisation for Economic Co-operation and Development, 2012).

^c^ Calculated by subtracting Early Development values from Current values.

**Supplemental Table 1.5**

***Demographics—Family and Household***

|  | Overall | Clinical | | | | | Non-Clinical |
| --- | --- | --- | --- | --- | --- | --- | --- |
|  |  | Overall | Professional Setting | | | |  |
|  |  |  | Institution Only | Institution/ Private Practice | Private Practice Only | Difference |  |
| Marital Status (%)^a,b^ |  |  |  |  |  |  |  |
| Currently Married | 75.4 | 75.2 | 68.1 | 81.8 | 79.6 | *χ^2^* (2, *N* = 307) = 6.32*, Cramer’s V = .14 | 78.8 |
| *Woman/Female* | 71.8 | 72.2 | 65.9 | 73.1 | 82.0 | *χ^2^* (2, *N* = 230) = 12.33**, Cramer’s V = .23 | 70.8 |
| *Man/Male* | 82.4 | 81.3 | 79.2 | 94.1 | 76.9 |  | 100 |
| Never Married | 13.9 | 13.8 | 20.7 | 11.4 | 7.8 | *χ^2^* (2, *N* = 307) = 8.28*, Cramer’s V = .16 | 12.1 |
| *Woman/Female* | 17.8 | 17.5 | 23.1 | 17.3 | 8.0 | † | 16.7 |
| *Man/Male* | 7.6 | 8.0 | 12.5 | 2.9 | 7.7 |  | 0 |
| Divorced | 6.5 | 6.8 | 6.0 | 4.5 | 8.7 | *χ^2^* (2, *N* = 307) = 1.44, Cramer’s V = .07 | 3.0 |
| *Woman/Female* | 5.6 | 5.7 | 6.6 | 3.8 | 6.0 | † | 4.1 |
| *Man/Male* | 8.4 | 8.9 | 4.2 | 5.9 | 11.5 |  | 0 |
| Number of Marriages (%)^c^ |  |  |  |  |  |  |  |
| One | 75.4 | 75.4 | 85.9 | 72.7 | 67.4 | *χ^2^* (6, *N* = 254) = 9.86, Cramer’s V = .14 | 70.4 |
| Two | 17.8 | 18.0 | 10.6 | 18.2 | 25.0 |  | 22.2 |
| Three or more | 5.4 | 5.1 | 2.4 | 7.8 | 5.4 |  | 7.4 |
| People Living in Household^f^ |  |  |  |  |  |  |  |
| *Mdn* [*IQR*] | 3 [2-4] | 3 [2-4] | 3 [2-4] | 3 [2-4] | 2 [2-4] |  | 3 [2-4] |
| *M* (*SD*) | 3.2 (1.3) | 3.2 (1.3) | 3.3 (1.4) | 3.2 (1.1) | 3.0 (1.3) | *F*(2,193) = 1.07, *η^2^* = .01 | 3.5 (1.7) |
| Household Makeup^a,d^ |  |  |  |  |  |  |  |
| Spouse/Partner(s) | 81.0 | 81.0 | 75.9 | 88.6 | 81.6 | *χ^2^* (2, *N* = 307) = 5.40, Cramer’s V = .13 | 81.8 |
| Dependent Child(ren) | 42.1 | 41.5 | 44.8 | 48.9 | 32.0 | *χ^2^* (2, *N* = 307) = 6.28*, Cramer’s V = .14 | 54.5 |
| Adult Child(ren) | 5.3 | 4.8 | 2.6 | 8.0 | 4.9 | *χ^2^* (2, *N* = 307) = 3.10, Cramer’s V = .10 | 9.1 |
| Dependent Children at Home (%)^e^ |  |  |  |  |  |  |  |
| One | 32.8 | 29.5 | 27.7 | 35.1 | 25.9 | *χ^2^* (4, *N* = 111) = 1.25, Cramer’s V = .08 | 47.1 |
| Two | 47.2 | 50.0 | 53.2 | 43.2 | 55.6 |  | 29.4 |
| Three or More | 20.0 | 20.5 | 19.1 | 21.6 | 18.5 |  | 23.5 |
| Languages Spoken at Home (%)^g^ |  |  |  |  |  |  |  |
| English Only | 91.9 | 92.5 | 93.0 | 93.2 | 93.1 | *χ^2^* (4, *N* = 304) = 2.61, Cramer’s V = .07 | 87.5 |
| English plus Another Language | 7.5 | 6.8 | 7.0 | 5.7 | 6.9 |  | 12.5 |

*Note.* Sample sizes generally include all eligible participants in the overall (*n* = 351), clinical (n = 323), and non-clinical samples (*n* = 35). Professional Setting further includes clinical participants working in institutional settings only (n = 119), both institutional settings and private practice (n = 90), or private practice only (n = 107). Actual sample sizes reduced due to item non-response. IQR = Interquartile range. **Bold** indicates significant difference based on measure of association (i.e., at least a medium effect per *η^2^* > .06 and Cramer’s V > .30; Cohen, 1988, 1992). †Data not provided due to limited sample size.

^a^ Multiple responses allowed.

^b^ Additional participants were widowed (*n* = 7), separated (*n* = 3), preferred to not respond (*n* = 5), or did not provide sufficient information (*n* = 14).

^c^ Among participants who had ever been married (*n* = 276). Additional participants preferred to not respond (*n* = 4).

^d^ Additional participants resided with parent(s) (*n* = 7), grandparent(s) (*n* = 3), other extended family member(s) (*n* = 9), other non-family individuals (*n* = 12), preferred to not respond (*n* = 7), or did not provide sufficient information (*n* = 14).

^e^ Among those with dependent children who responded to this item (*n* = 125).

^f^ Calculated total of reported spouses/partners, dependent children, adult children, parents, grandparents, other extended family members, and other non-family individuals, including the participant.

^g^ Additional participants preferred to not respond (*n* = 2) or did not provide sufficient information (*n* = 17). Other languages include Spanish (*n* = 11), French (*n* = 3), Japanese (*n* = 2), Mandarin (*n* = 3), Arabic, German, Hindi, Italian, Russian, Polish, Portuguese, Swedish, and Yiddish (all *n* = 1).

**p* < .05. ***p* < .01. ****p* < .001.

**Supplemental Table 2.1**

***Training—Clinical Forensic Psychology Participants***

|  | Overall | Professional Setting | | | |
| --- | --- | --- | --- | --- | --- |
|  |  | Institution Only | Institution/ Private Practice | Private Practice Only | Difference |
| Degree (%)^a^ |  |  |  |  |  |
| PhD | 61.1 | 56.3 | 59.3 | 67.9 | *χ^2^* (8, *N* = 311) = 15.42, Cramer’s V = .16 |
| PsyD | 34.5 | 38.7 | 39.5 | 26.4 |  |
| Program (%)^b^ |  |  |  |  |  |
| Clinical Psychology | 70.6 | 70.6 | 67.8 | 72.6 | *χ^2^* (8, *N* = 312) = 11.93, Cramer’s V = .14 |
| Counseling Psychology | 11.4 | 7.6 | 13.8 | 14.2 |  |
| Clinical Forensic Psychology | 8.2 | 13.4 | 8.0 | 2.8 |  |
| Forensic Psychology | 5.7 | 5.9 | 5.7 | 4.7 |  |
| Other | 4.1 | 2.5 | 4.6 | 5.7 |  |
| Program—APA/CPAAccredited^c^ |  |  |  |  |  |
| Yes | 84.5 | 87.4 | 85.1 | 80.2 | *χ^2^* (6, *N* = 312) = 4.29, Cramer’s V = .08 |
| No | 12.3 | 10.1 | 12.6 | 15.1 |  |
| Predoctoral Internship (%) |  |  |  |  |  |
| Yes | 94.3 | 95.8 | 97.7 | 89.6 | *χ^2^* (2, *N* = 312) = 6.61*, Cramer’s V = .15 |
| No | 5.7 | 4.2 | 2.3 | 10.4 |  |
| Predoctoral Internship— APA/CPA Accredited (%)^d^ |  |  |  |  |  |
| Yes | 84.5 | 86.8 | 84.7 | 81.1 | *χ^2^* (4, *N* = 294) = 3.41, Cramer’s V = .08 |
| No | 14.2 | 13.2 | 12.9 | 16.8 |  |
| Postdoctoral Fellowship (%) |  |  |  |  |  |
| Yes | 53.5 | 53.8 | 47.1 | 59.8 | *χ^2^* (2, *N* = 308) = 3.04, Cramer’s V = .10 |
| No | 46.5 | 46.2 | 52.9 | 40.2 |  |
| Postdoctoral Fellowship, by Career Stage (Yes %)^e^ |  |  |  |  |  |
| Early Career | 62.0 | 66.2 | 39.3 | 75.0 | † |
| Mid-Career | 53.3 | 33.3 | 62.9 | 66.7 |  |
| Senior Career | 52.5 | 20.0 | 50.0 | 63.5 |  |
| Late Senior Career | 33.3 | † | 23.1 | 40.0 |  |
| Postdoctoral Fellowship, by Career Stage and Gender (Female Yes %)^e^ |  |  |  |  |  |
| Early Career | 55.6 | 60.6 | 28.6 | 73.3 | † |
| Mid-Career | 51.6 | 30.8 | 60.9 | 76.9 |  |
| Senior Career | 47.1 | † | † | † |  |
| Late Senior Career | 25.0 | † | † | 30.0 |  |

*Note.* Sample sizes generally include all eligible participants in the overall (*n* = 351) and clinical samples (n = 323). Professional Setting further includes clinical participants working in institutional settings only (n = 119), both institutional settings and private practice (n = 90), or private practice only (n = 107). Actual sample sizes reduced due to item non-response. **Bold** indicates significant difference based on measure of association (i.e., at least a medium effect per *η^2^* > .06 and Cramer’s V > .30; Cohen, 1988, 1992). †Data not provided due to limited sample size.

^a^ Additional degrees include terminal MA (*n* = 6), EdD (*n* = 3), and Other [*n* = 5, including: other MA (*n* = 2), MSW *n* = 2, and M.Ed. (*n* = 1), among others]. Additional participants did not provide sufficient information (*n* = 7).

^b^ Additional programs include School Psychology (*n* = 4) and other applied areas including Neuropsychology (*n* = 2), Experimental (*n* = 2), Clinical Health Psychology, Developmental Psychology, Industrial/Organizational Psychology, and Social Work (all *n* = 1). Additional participants did not provide sufficient information (*n* = 7).

^c^ Additional participants responded “I don’t know” (*n* = 4) or “Not applicable” (*n* = 6). Additional participants did not provide sufficient information (*n* = 7).

^d^ Additional participants responded “I don’t know” (*n* = 4).

^e^ Based on number of years since participants’ highest degree (first used if multiple) for early career (1-10 years), mid-career (11-20 years), senior (21-30 years), and late senior career (31-40 years). Additional participants did not provide sufficient information (*n* = 6).

**p* < .05. ***p* < .01. ****p* < .001.

**Supplemental Table 2.2**

***Training—Non-Clinical Forensic Psychology Participants***

|  | Overall |
| --- | --- |
| Degree (%)^a^ |  |
| PhD | 75.0 |
| MA (terminal) | 15.6 |
| Program (%)^b^ |  |
| Psychology & Law | 22.6 |
| Social Psychology | 22.6 |
| Experimental Psychology | 6.5 |
| Psychology & Social Behavior | 6.5 |
| Postdoctoral Fellowship (%)^c^ |  |
| Yes | 10.7 |
| No | 89.3 |

*Note.* Sample sizes generally include all eligible participants in the non-clinical sample (n = 35).

^a^ Additional participants reported Other (*n* = 5; e.g., n = 1 PsyD, BS, and “N/A”) or did not provide sufficient information (*n* = 3).

^b^ Additional programs include Developmental Psychology (*n* = 1), Legal Psychology (*n* = 1), and Other [*n* = 11, including: Forensic Psychology (*n* = 2), Clinical Psychology, Cognitive Psychology, Community Psychology, I/O Psychology, Philosophy, and Social science – interdisciplinary degree (all *n* = 1)]. Additional participants did not provide sufficient information (*n* = 4).

^c^ Additional participants did not provide sufficient information (*n* = 7).

**Supplemental Table 2.3**

***Training—Funding***

|  | Overall | Clinical | | | | | Non-Clinical |
| --- | --- | --- | --- | --- | --- | --- | --- |
|  |  | Overall | Professional Setting | | | |  |
|  |  |  | Institution Only | Institution/ Private Practice | Private Practice Only | Difference |  |
| Graduate Funding (%)^a^ |  |  |  |  |  |  |  |
| Student Loans | 67.6 | 69.9 | 79.0 | 70.0 | 59.8 | *χ^2^* (2, *N* = 316) = 9.86**, Cramer’s V = .18 | 38.2 |
| Graduate Stipends | 46.2 | 45.1 | 45.4 | 45.6 | 44.9 | *χ^2^* (2, *N* = 316) = 0.01, Cramer’s V = .01 | 55.9 |
| External Employment | 37.0 | 37.9 | 41.2 | 34.4 | 38.3 | *χ^2^* (2, *N* = 316) = 0.98, Cramer’s V = .06 | 32.4 |
| Teaching Assistantships | 37.0 | 36.7 | 37.0 | 37.8 | 35.5 | *χ^2^* (2, *N* = 316) = 0.11, Cramer’s V = .02 | 41.2 |
| Tuition Remission—Full | 24.9 | 22.3 | 24.4 | 21.1 | 20.6 | *χ^2^* (2, *N* = 316) = 0.55, Cramer’s V = .04 | 50.0 |
| Research Assistantships | 23.4 | 21.0 | 20.2 | 22.2 | 19.6 | *χ^2^* (2, *N* = 316) = 0.22, Cramer’s V = .03 | 52.9 |
| Tuition Remission—Partial | 15.3 | 15.7 | 17.6 | 13.3 | 15.0 | *χ^2^* (2, *N* = 316) = 0.76, Cramer’s V = .05 | 11.8 |
| Personal Loans | 9.8 | 10.0 | 9.2 | 11.1 | 10.3 | *χ^2^* (2, *N* = 316) = 0.20, Cramer’s V = .03 | 5.9 |
| Administrative Positions | 4.9 | 5.3 | 5.9 | 4.4 | 5.6 | *χ^2^* (2, *N* = 316) = 0.23, Cramer’s V = .03 | 0 |
| Other | 17.1 | 17.2 | 13.4 | 20.0 | 19.6 | *χ^2^* (2, *N* = 316) = 2.09, Cramer’s V = .08 | 14.7 |
| Graduate Stipend^b^ (*Mdn* [*IQR*]) | 12 [7-18] | 12 (7-17) | 12 (10-19) | 10 (4-15) | 11 (7-15) |  | 14  [3-18] |
| *M* (*SD*) | 14 (12) | 14 (12) | 16 (12) | 13 (16) | 11 (7) | *F*(2,104) = 0.27, *η_p_^2^* = 0.005 | 13 (8) |
| Postdoctoral Fellowship |  |  |  |  |  |  |  |
| Funded (%)^c^ | 44.9 | 44.0 | 51.6 | 43.9 | 36.1 | *χ^2^* (2, *N* = 166) = 3.05, Cramer’s V = .14 | 66.0 |
| Stipend^d^ (*Mdn* [*IQR*]) | 35  [21-40] | 35  [24-40] | 37  [33-46] | 25  [18-32] | 29  [19-35] |  | - |
| *M* (*SD*) | 32 (15) | 33 (15) | 40 (15) | 24 (11) | 28 (12) | *F*(2,64) = 3.07, *η_p_^2^* = **0.09** | - |

*Note.* Sample sizes generally include all eligible participants in the overall (*n* = 351), clinical (n = 323), and non-clinical samples (*n* = 35). Professional Setting further includes clinical participants working in institutional settings only (n = 119), both institutional settings and private practice (n = 90), or private practice only (n = 107). Actual sample sizes reduced due to item non-response. IQR = Interquartile range. **Bold** indicates significant difference based on measure of association (i.e., at least a medium effect per *η_p_^2^* > .06 and Cramer’s V > .30; Cohen, 1988, 1992).

^a^ Information regarding predoctoral internship funding was not collected. Other sources (*k* = 7) include personal funds, support from family or spouse/partner, paid clinical practica/externship placements, military scholarships and related programs, and external research fellowships, among others. Additional participants did not provide sufficient information (*n* = 5).

^b^ Values are in thousands of USD rounded from participants’ pooled responses. Based on number of participants who reported graduate stipend funding (*n* = 117 overall, *n* = 104 clinical, *n* = 19 non-clinical). Comparison included participants’ years in the field as a covariate, based on the significant differences across professional settings for this variable (Supplemental Table 1.1).

^c^ Based on number of participants who completed postdoctoral fellowship (*n* = 167 overall, *n* = 166 clinical, *n* = 3 non-clinical).

^d^ Values are in thousands of USD rounded from participants’ pooled responses. Based on number of participants who completed postdoctoral fellowship and reported a stipend amount (*n* = 64 overall and *n* = 104 clinical). Clinical comparison included participants’ years in the field as a covariate, based on the significant differences across professional settings for this variable (see Supplemental Table 1.1). Non-clinical participants excluded due to limited responses (*n* = 1).

**p* < .05. ***p* < .01. ****p* < .001.

**Supplemental Table 2.4**

***Training—Student Loan Debt***

|  | Overall | Clinical | | | | | | Non-Clinical |
| --- | --- | --- | --- | --- | --- | --- | --- | --- |
|  |  | Overall | | Professional Setting | | | |  |
|  |  | |  | Institution Only | Institution/ Private Practice | Private Practice Only | Difference |  |
| Upon Graduation^a^ |  | |  |  |  |  |  |  |
| Overall |  | |  |  |  |  |  |  |
| *Mdn* [IQR] | 50 [0-125] | | 60 [4-130] | 90 [35-200] | 60 [10-150] | 20 [0-80] |  | 0 [0-19] |
| *M* (*SD*) | 85 (100) | | 90 (101) | 122 (112) | 88 (91) | 57 (86) | *F*(2,311) = 0.23, *η_p_^2^* = 0.001 | 22 (48) |
| Overall, by Career Stage [*M*(*SD*)] |  | |  |  |  |  |  |  |
| *Early Career* | 139 (117) | | 148 (116) | 152 (116) | 162 (102) | 118 (131) | *F*(2,128) = 1.04, *η_p_^2^* = 0.016 | 40 (70) |
| *Mid-Career* | 78 (70) | | 83 (70) | 85 (83) | 73 (59) | 93 (69) | *F*(2,93) = 0.62, *η_p_^2^* = 0.013 | 22 (42) |
| *Senior Career* | 21 (30) | | 23 (31) | 11 (14) | 21 (33) | 27 (34) | *F*(2,38) = 0.52, *η_p_^2^* = 0.029 | 4 (11) |
| *Late Senior Career* | 9 (16) | | 9 (16) | 4 (4) | 11 (14) | 8 (15) | *F*(2,48) = 0.45, *η_p_^2^* = 0.019 | 10 (20) |
| *Difference* |  | | *F*(3,312) = 43.30***, *η_p_^2^* = **0.30** | *F*(3,117) = 7.11***, *η_p_^2^* = **0.16** | *F*(3,88) = 19.61***, *η_p_^2^* = **0.41** | *F*(3,103) = 13.54***, *η_p_^2^* = **0.29** |  | † |
| Clinical—by degree |  | |  |  |  |  |  |  |
| *PhD* |  | |  |  |  |  |  |  |
| *Mdn* [IQR] |  | | 30 [0-83] | 62 [14-116] | 20 [0-68] | 9 [0-50] |  | - |
| *M* (*SD*) |  | | 59 (79) | 87 (87) | 52 (76) | 38 (66) | *F*(2,188) = 0.77, *η_p_^2^* = 0.008 | - |
| *PsyD* |  | |  |  |  |  |  |  |
| *Mdn* [IQR] |  | | 122  [70-205] | 130  [90-280] | 140  [80-200] | 93  [30-163] |  | - |
| *M* (*SD*) |  | | 148 (113) | 176 (129) | 142 (87) | 113 (109) | *F*(2,107) = 0.07, *η_p_^2^* = 0.001 | - |
| Current^b^ |  | |  |  |  |  |  |  |
| Overall |  | |  |  |  |  |  |  |
| *Mdn* [IQR] | 0 [0-90] | | 0 [0-100] | 40 [1-170] | 0 [0-114] | 0 [0-26] |  | 0 [0-0] |
| *M* (*SD*) | 64 (11) | | 68 (110) | 98 (132) | 69 (102) | 33 (75) | *F*(2,312) = 0.43, *η_p_^2^* = 0.003 | 16 (46) |
| Clinical—by degree |  | |  |  |  |  |  |  |
| *PhD* |  | |  |  |  |  |  |  |
| *Mdn* [IQR] |  | | 0 [0-40] | 20 [0-55] | 0 [0-41] | 0 [0-0] |  |  |
| *M* (*SD*) |  | | 38 (77) | 56 (90) | 39 (80) | 19 (56) | *F*(2,189) = 0.77, *η_p_^2^* = 0.003 |  |
| *PsyD* |  | |  |  |  |  |  |  |
| *Mdn* [IQR] |  | | 82 [0-233] | 110 [8-265] | 87 [0-199] | 0 [0-100] |  |  |
| *M* (*SD*) |  | | 123 (140) | 163 (163) | 117 (117) | 70 (105) | *F*(2,107) = 0.21, *η_p_^2^* = 0.004 |  |

*Note.* Values are in thousands of USD rounded from participants’ pooled responses. Sample sizes generally include all eligible participants in the overall (*n* = 351), clinical (n = 323), and non-clinical samples (*n* = 35). Professional Setting further includes clinical participants working in institutional settings only (n = 119), both institutional settings and private practice (n = 90), or private practice only (n = 107). Actual sample sizes reduced due to item non-response. IQR = Interquartile range. **Bold** indicates significant difference based on measure of association (i.e., at least a medium effect per *η_p_^2^* > .06; Cohen, 1988, 1992). †Data not provided due to limited sample size.

^a^ Additional participants did not provide sufficient information (*n* = 11). Comparison included participants’ years in the field as a covariate, based on the significant differences across professional settings for this variable (Supplemental Table 1.1).

^b^ Additional participants did not provide sufficient information (*n* = 9). Comparison included participants’ years in the field as a covariate, based on the significant differences across professional settings for this variable (Supplemental Table 1.1).

**p* < .05. ***p* < .01. ****p* < .001.

**Supplemental Table 3.1**

***Professional Practice–General***

|  | Overall | Clinical | | | | | Non-Clinical |
| --- | --- | --- | --- | --- | --- | --- | --- |
|  |  | Overall | Professional Setting | | | |  |
|  |  |  | Institution Only | Institution/ Private Practice | Private Practice Only | Difference |  |
| Licensed (%) |  |  |  |  |  |  |  |
| Yes | - | 93.3 | 90.8 | 96.6 | 95.3 | *χ^2^* (2, *N* = 312) = 3.48, Cramer’s V = .11 | - |
| No | - | 6.7 | 9.2 | 3.4 | 4.7 |  | - |
| Time to Licensure^a^ |  |  |  |  |  |  |  |
| *Mdn_years_* [*IQR*] | - | 1 [1-2] | 1 [1-2] | 1 [1-2] | 1 [1.5-2] |  | - |
| *M_years_* (*SD*) | - | 1.7 (1.5) | 1.5 (1.1) | 1.6 (1.3) | 1.9 (1.9) | *F*(2,283) = 1.70,  *η^2^* = 0.01 | - |
| Position type (%)^b^ |  |  |  |  |  |  |  |
| Full-time | 79.7 | 79.2 | 93.2 | 66.7 | 75.7 | *χ^2^* (6, *N* = 315) = 42.82***, Cramer’s V = .26 | 82.4 |
| Full-time with a Secondary  Part-Time Position | 8.1 | 8.8 | 2.5 | 22.2 | 4.7 |  | 8.8 |
| Part-time | 11.0 | 11.3 | 4.2 | 11.1 | 18.7 |  | 2.9 |
| *Early Career* | 5.7 | 5.4 | 2.6 | 6.9 | 12.0 |  | 8.3 |
| *Mid-Career* | 8.8 | 9.6 | 6.1 | 11.1 | 12.0 |  | 0 |
| *Senior Career* | 12.8 | 12.2 | 0 | 10.0 | 16.0 |  | 12.5 |
| *Late Senior Career* | 28.8 | 30.0 | 25.0 | 21.4 | 32.3 |  | 20 |
| Contract Period (%)^c^ |  |  |  |  |  |  |  |
| 9-10 months | 15.2 | 11.1 | 9.3 | 13.5 | - |  | 57.1 |
| 11-12 months | 80.8 | 84.5 | 88.1 | 79.8 | - |  | 42.9 |
| Amount Worked Compared to  Prior Year (%)^d^ |  |  |  |  |  |  |  |
| About the Same | 84.7 | 85.0 | 91.6 | 85.6 | 77.6 | *χ^2^* (4, *N* = 316) = 9.43, Cramer’s V = .12 | 82.4 |
| Significantly More | 10.1 | 10.0 | 6.7 | 8.9 | 15 |  | 11.4 |
| Significantly Less | 5.2 | 5.0 | 1.7 | 5.6 | 7.5 |  | 5.9 |
| Languages Spoken at Work (%)^e^ |  |  |  |  |  |  |  |
| English Only | 94.3 | 93.9 | 94.8 | 95.5 | 92.2 | *χ^2^* (2, *N* = 307) = 1.05, Cramer’s V = .06 | 97.0 |
| English and Other Language | 5.7 | 6.1 | 5.2 | 4.5 | 7.8 |  | 2.9 |
| Work Locations (Days per week) |  |  |  |  |  |  |  |
| Office Setting |  |  |  |  |  |  |  |
| *Mdn* [*IQR*] | 4 [2-5] | 4 [2-5] | 5 [4-5] | 4 [2-5] | 3 [1-4] |  | 3.5 [2-5] |
| *M* (*SD*) | 3.5 (1.8) | 3.5 (1.8) | 4.1 (1.6) | 3.7 (1.7) | 2.8 (1.9) | *F*(2,290) = 10.05***, *η^2^* = **0.07** | 3.3 (1.8) |
| Remote Setting |  |  |  |  |  |  |  |
| *Mdn* [*IQR*] | 0 [0-2] | 0 [0-1] | 0 [0-1] | 1 [0-2] | 0 [0-1.3] |  | 1 [0-3] |
| *M* (*SD*) | 1.0 (1.3) | 0.9 (1.3) | 0.7 (1.3) | 1.1 (1.3) | 0.9 (1.2) | *F*(2,288) = 1.95, *η^2^* = 0.01 | 1.5 (1.4) |
| Other Setting^f^ |  |  |  |  |  |  |  |
| *Mdn* [*IQR*] | 0 [0-0] | 0 [0-0] | 0 [0-0] | 0 [0-0] | 0 [0-1] |  | 0 [0-0] |
| *M* (*SD*) | 0.4 (0.9) | 0.4 (1.0) | 0.01 (0.3) | 0.3 (0.7) | 0.8 (1.4) | *F*(2,292) = 9.26***, *η^2^* = **0.06** | 0.2 (0.6) |

*Note.* Sample sizes generally include all eligible participants in the overall (*n* = 351), clinical (n = 323), and non-clinical samples (*n* = 35). Professional Setting further includes clinical participants working in institutional settings only (n = 119), both institutional settings and private practice (n = 90), or private practice only (n = 107). Actual sample sizes reduced due to item non-response. IQR = Interquartile range. **Bold** indicates significant difference based on measure of association (i.e., at least a medium effect per *η^2^* > .06 and Cramer’s V > .30; Cohen, 1988, 1992).

^a^ Calculated based on reported year of highest clinical degree and year of licensure.

^b^ Additional participants reported being retired (*n* = 4) or did not provide sufficient information (*n* = 6).

^c^ Based on all eligible participants who reported working in institutional settings (*n* = 224; i.e., excluding Private Practice Only). Additional participants reported other (*n* = 9) or did not provide sufficient information (*n* = 2).

^d^ Self-defined by participants as compared to 2017. Additional participants did not provide sufficient information (*n* = 5).

^e^ Other languages include Spanish (*n* = 13), French (*n* = 3), German, Arabic, Polish, Portuguese, and Mandarin (all *n* = 1). Additional participants did not provide sufficient information (*n* = 16).

^f^ Other settings (*n* = 51) including other field settings not considered office setting by participants (e.g., jails, prisons, courts, hospitals, “on site,” and similar), home settings not considered remote setting by participants, or in transit related to work, among others.

**p* < .05. ***p* < .01. ****p* < .001.

**Supplemental Table 3.2**

***Professional Practice—Board Certification***

|  | Overall | Professional Setting | | | |
| --- | --- | --- | --- | --- | --- |
|  |  | Institution Only | Institution/ Private Practice | Private Practice Only | Difference |
| Boarded—Any (%)^a^ | 29.8 | 16.8 | 36.8 | 39.6 | *χ^2^* (4, *N* = 312) = 19.35***, Cramer’s V = .18 |
| Boarded—Any ABPP^b^ |  |  |  |  |  |
| Overall Sample (%) | 28.8 | 17.4 | 37.2 | 34.6 | *χ^2^* (2, *N* = 305) = 11.97**, Cramer’s V = .20 |
| Boarded Sample (%) | 93.6 | 100 | 100 | 90.0 | *χ^2^* (2, *N* = 92) = 5.44, Cramer’s V = .24 |
| Boarded—ABFP |  |  |  |  |  |
| Overall Sample (%) | 24.1 | 14.8 | 32.6 | 27.4 | *χ^2^* (2, *N* = 307) = 9.44**, Cramer’s V = .18 |
| Boarded Sample (%) | 78.7 | 85.0 | 87.5 | 69.0 | *χ^2^* (2, *N* = 94) = 4.29, Cramer’s V = .21 |
| Years to Certification [M(SD)]^c^ |  |  |  |  |  |
| Overall | 10.0 (4.9) | 8.3 (4.9) | 9.0 (3.6) | 12.1 (5.4) | *F*(2,60) = 3.92*, *η^2^* = **0.12** |
| Postdoctoral Fellowship |  |  |  |  |  |
| *Yes* | 9.0 (5.0) | 7.4 (5.8) | 8.0 (3.0) | 11.2 (5.7) |  |
| *No* | 11.4 (4.3) | 10 (1.9) | 10.3 (4.1) | 13.4 (5.0) |  |

*Note.* ABFP = American Board of Forensic Psychology. ABPP = American Board of Professional Psychology. Additional participants reported certification through the American Board of Professional Neuropsychology, American Board of Assessment Psychology, American College of Forensic Psychology, and National Board of Forensic Evaluators (all n = 1); these participants are not included in additional subgroup analyses due to limited sample size. Overall includes all eligible clinical participants (n = 323). Professional Setting further includes participants working in institutional settings only (n = 119), both institutional settings and private practice (n = 90), or private practice only (n = 107). Postdoctoral Fellowship further includes participants boarded through ABFP who completed postdoctoral fellowships (*n* = 37) or not (*n* = 24). Actual sample sizes reduced due to item non-response. IQR = Interquartile range. **Bold** indicates significant difference based on measure of association (i.e., at least a medium effect per *η^2^* > .06 and Cramer’s V > .30; Cohen, 1988, 1992).

^a^ Additional participants preferred to not respond (*n* = 7).

^b^ Specific ABPP-affiliated boards include the American Board of Clinical Psychology (*n* = 10), American Board of Clinical Neuropsychology (*n* = 4), American Board of Clinical Child & Adolescent Psychology (*n* = 3), American Board of Counseling Psychology, American Board of Couple & Family Psychology, American Board of Geropsychology, American Board of School Psychology, and American Board of Professional Psychology (unspecified) (all *n* = 1).

^c^ Calculated based on reported year of highest relevant degree and ABFP board certification. Comparison included participants’ years in the field as a covariate, based on the significant differences across professional settings for this variable (Supplemental Table 1.1).

**p* < .05. ***p* < .01. ****p* < .001.

**Supplemental Table 3.3**

***Professional Practice–Institutional Settings, Departments, and Titles for Clinical Forensic Psychology Participants***

|  | Overall | | Professional Setting | | | | | |
| --- | --- | --- | --- | --- | --- | --- | --- | --- |
|  | % | *Hours per week* [*M* (*SD*)] | Institution Only | | | Institution/ Private Practice | | |
|  |  |  | % | *Hours per week* [*M* (*SD*)] | | % | *Hours per week* [*M* (*SD*)] | |
| Setting |  | |  | | |  | | |
| Applied^a^ | 77.5 |  | 80.7 | |  | 73.3 | |  |
| *Public Psychiatric Hospital* | 36.4 | 36.0 (12.8) | 39.5 | | 37.6 (12.3) | 32.2 | | 33.4 (13.3) |
| *Court Clinic* | 12.0 | 32.1 (23.0) | 10.9 | | 41.7 (22.8) | 13.3 | | 21.6 (18.9) |
| *Public Defenders’ Office* | 10.0 | 2.0 (0) | 1.7 | | 0.9 (0.2) | 21.1 | | 12.3 (12.7) |
| *Federal Prison* | 7.2 | 41.6 (4.3) | 9.2 | | 42.1 (4.8) | 4.4 | | 40 (0) |
| *Local Jail* | 6.7 | 28.2 (34.7) | 5.0 | | 7.0 (6.0) | 8.9 | | 43.4 (39.2) |
| *State Prison* | 6.2 | 29.3 (15.5) | 8.6 | | 34.3 (15.5) | 5.6 | | 21.6 (13.9) |
| *Forensic Juvenile Facility* | 4.8 | 34.9 (36.7) | 5.9 | | 21.1 (18.7) | 3.3 | | 67.0 (52.9) |
| *Primary University Hospital/Academic Medical Center* | 1.9 | 40.56 (31.1) | 5.0 | | 40.56 (31.1) | 0 | | - |
| Academic^b^ | 17.7 |  | 12.6 | |  | 24.4 | |  |
| *Four-Year University/College (Non-Medical) with Doctoral Psychology Program* | 8.1 | 28.1 (15.8) | 3.4 | | 31.0 (18.3) | 14.4 | | 27.2 (15.6) |
| *Four-Year University/College (Non-Medical) without Doctoral Psychology Program* | 5.3 | 29.7 (16.5) | 5.9 | | 33.0 (19.6) | 4.4 | | 25.5 (13.2) |
|  | Overall (%) | | Professional Setting (%) | | | | | |
|  |  |  | Institution Only | | | Institution/ Private Practice | | |
| Department^c^ |  | |  | | |  | | |
| Psychology | 74.5 | | 73.7 | | | 75.6 | | |
| Criminal Justice | 13.0 | | 8.5 | | | 18.9 | | |
| Psychiatry | 5.3 | | 6.8 | | | 3.3 | | |
| Other | 9.6 | | 8.5 | | | 2.2 | | |
| Title^d^ |  | |  | | |  | | |
| Staff Psychologist | 52.9 | | 56.8 | | | 47.8 | | |
| Assistant Professor | 9.1 | | 11.0 | | | 6.7 | | |
| Professor | 5.3 | | 1.7 | | | 10.0 | | |
| Other | 25.5 | | 24.6 | | | 26.7 | | |

*Note.* Related specifically to forensic psychology activities only. All items allowed for multiple responses. Includes all eligible clinical forensic psychology participants reporting working in any institutional setting (*n* = 209). Professional Setting further includes participants working in institutional settings only (*n* = 119) or both institutional settings and private practice (*n* = 90). Actual sample sizes reduced due to item non-response.

^a^ Among a variety of other applied settings in criminal justice, medical, clinical, military, and other (all < 5% overall).

^b^ Among a variety of other academic settings in higher education, medical, research, and other (all < 5% overall).

^c^ “Other” responses included various forensic-focused departments (*n* = 8) and administrative departments (*n* = 3), among others. Additional participants responded Public Health (*n* = 8), Social Science (*n* = 4), and Criminology (*n* = 3), or preferred to not respond to this item (*n* = 5).

^d^ “Other” responses included various clinical titles (e.g., those including “psychologist,” “evaluator,” or “clinical consultant” in the title) (*n* = 19) and administrative titles (*n* = 11), among others. Additional participants responded Clinical Program Director, Director of Clinical Training, and Division Head (*n* = 8); Associate Professor (*n* = 7); Department Chair (*n* = 6); Lecturer/Instructor (*n* = 5); and Dean, Distinguished Professor, and Research Program Director (*n* = 1); or preferred to not respond to this item (*n* = 2).

**Supplemental Table 3.4**

***Professional Practice – Institutional Settings, Departments, and Titles for Non-Clinical Forensic Psychology Participants***

|  | % | Hours Per Week *[M(SD)]* |
| --- | --- | --- |
| Setting^a^ |  |  |
| Academic | 90.5 |  |
| *Four-Year University/College (Non-Medical; with a Doctoral Psychology Program)* | 47.6 | 37.7 (22.4) |
| *Four-Year University/College (Non-Medical; without a Doctoral Psychology Program)* | 33.3 | 36.8 (15.9) |
| *Professional School of Psychology* | 9.5 | 42.5 (10.6) |
| Applied | 19.0 |  |
| *Public Defenders’ Office* | 14.3 | 12.7 (12.0) |
| *District Attorney’s Office* | 14.3 | 2.7 (2.1) |
| Department^b^ |  |  |
| Psychology | 76.2 |  |
| Criminal Justice | 23.8 |  |
| Social Science | 9.5 |  |
| Other | 9.5 |  |
| Title^c^ |  |  |
| Associate Professor | 38.1 |  |
| Assistant Professor | 23.8 |  |
| Professor | 14.3 |  |
| Lecturer/Instructor | 9.5 |  |
| Other | 23.8 |  |

*Note.* Related specifically to forensic psychology activities only. All items allowed for multiple responses. Includes all eligible non-clinical forensic psychology participants reporting working in any institutional setting (*n* = 21). Actual sample sizes reduced due to item non-response.

^a^ “Which of the following best describes the primary institution(s) from which you received income for forensic psychology work activities in 2018?” and “Approximately how many hours per week (on average) did you spend in the following institutions completing forensic psychology work activities in 2018?” Additional responses for Academic included doctoral psychology program in medical/educational setting (no undergraduate program) (*n* = 1). Additional responses for Applied included court clinic, juvenile forensic facility, and state office of mental health (all *n* = 1).

^b^ “Which of the following best describes the primary institutional department(s) from which you received income for forensic psychology work activities in 2018?” Other responses included Forensic Studies and Gender & Women’s Studies (both *n* = 1).

^c^ “Which of the following best describes the primary institutional title(s) from which you received income for forensic psychology work activities in 2018?” Other responses included various administrative titles (*n* = 3), among others. Additional participants responded Distinguished Professor, Division Head, and Staff Psychologist (all *n* = 1).

**Supplemental Table 3.5**

***Professional Practice – Forensic Psychology Private Practice Roles***

|  | Overall | Clinical | | | Non-Clinical |
| --- | --- | --- | --- | --- | --- |
|  |  | Overall | Professional Setting | |  |
|  |  |  | Private Practice Only | Institution/ Private Practice |  |
| Outside Contractor | 39.3 | 38.3 | 48.9 | 29.3 | 42.9 |
| Sole Proprietor | 25.6 | 27.6 | 24.4 | 30.2 | 19.0 |
| Partner | 24.2 | 23.5 | 18.9 | 27.4 | 23.8 |
| Employee | 9.5 | 9.7 | 7.8 | 11.3 | 4.8 |

*Note.* “What was your role in private practice in 2018? (select all that apply)” Related specifically to forensic psychology activities only. Outside Contractor = received a 1099 IRS tax form at year end. Sole Proprietor = self-identified as sole proprietor, self-employed, owner, independent, or similar. Employee = Received a W-2 IRS tax form at year end. Allowed for multiple responses. Based on participants working in any private practice setting in the overall (*n* = 211), clinical (*n* = 196), and non-clinical group (*n* = 21). Professional Setting further includes clinical forensic psychology participants working in any private practice setting in private practice settings only (*n* = 119) or both institutional and private practice settings (*n* = 90). Actual sample sizes reduced due to item non-response. Additional participants responded Other (*n* = 7) or preferred to not respond to this item (*n* = 5).

**Supplemental Table 3.6**

***Professional Practice–Work Activities for Clinical Forensic Psychology Participants***

|  | Overall | Professional Setting | | | |
| --- | --- | --- | --- | --- | --- |
|  |  | Institution Only | Institution/ Private Practice | Private Practice Only | Difference |
| Clinical practice—Forensic assessment/evaluation (%) | 83.0 | 73.7 | 91.1 | 86.8 | *χ^2^* (2, *N* = 314) = 12.53**, Cramer’s V = .20 |
| *Hours Per Week [M(SD)]* | 23.1 (20.9) | 26.0 (20.6) | 22.1 (23.9) | 20.8 (18.1) | *F*(2,221) = 1.28,  *η_p_^2^* = 0.01 |
| Expert Witness (%) | 71.3 | 56.8 | 71.1 | 88.7 | *χ^2^* (2, *N* = 314) = 28.0***, Cramer’s V = **.30** |
| *Hours Per Week [M(SD)]* | 3.2 (4.2) | 1.7  (2.4) | 3.3  (3.4) | 4.3 (5.4) | *F*(2,180) = 6.26**,  *η_p_^2^* = **0.07** |
| Case Consultation (%) | 53.6 | 41.5 | 52.2 | 67.9 | *χ^2^* (2, *N* = 314) = 15.73***, Cramer’s V = .22 |
| *Hours Per Week [M(SD)]* | 6.2 (10.6) | 6.5 (11.7) | 3.2 (2.4) | 8.1 (12.9) | *F*(2,126) = 2.25,  *η_p_^2^* = 0.04 |
| Supervision—Clinical Trainees (%) | 35.6 | 53.4 | 38.9 | 14.2 | *χ^2^* (2, *N* = 314) = 37.78***, Cramer’s V = .**35** |
| *Hours Per Week [M(SD)]* | 5.0 (4.7) | 4.8  (4.9) | 5.2  (4.9) | 5.5 (3.5) | *F*(2,99) = 0.12,  *η_p_^2^* = 0.003 |
| Teaching (%) | 29.0 | 29.7 | 37.8 | 21.7 | *χ^2^* (2, *N* = 314) = 6.09*, Cramer’s V = .14 |
| *Hours Per Week [M(SD)]* | 7.6 (9.5) | 8.9  (11.4) | 8.2  (9.6) | 4.5 (3.5) | *F*(2,79) = 1.36,  *η_p_^2^* = 0.04 |
| Assessment of court functioning and administrative processes (%) | 23.7 | 20.3 | 24.4 | 26.4 | *χ^2^* (2, *N* = 314) = 1.98, Cramer’s V = .06 |
| *Hours Per Week [M(SD)]* | 14.4 (18.3) | 12.7 (14.7) | 17.3 (24.5) | 13.29 (14.3) | *F*(2,57) = 0.36,  *η_p_^2^* = 0.01 |
| Clinical practice—Forensic intervention (%) | 22.7 | 26.3 | 22.2 | 19.8 | *χ^2^* (2, *N* = 314) = 1.35, Cramer’s V = .06 |
| *Hours Per Week [M(SD)]* | 9.4 (11.3) | 10.3 (12.8) | 9.8  (12.0) | 7.5 (7.4) | *F*(2,60) = 0.31,  *η_p_^2^* = 0.01 |
| Clinical practice—General assessment/evaluation (non-forensic) (%) | 22.1 | 21.2 | 16.7 | 27.4 | *χ^2^* (2, *N* = 314) = 3.31, Cramer’s V = .10 |
| *Hours Per Week [M(SD)]* | 9.5 (10.7) | 8.0  (6.4) | 17.3 (24.5) | 12.1 (14.6) | *F*(2,54) = 0.96,  *η_p_^2^* = 0.04 |
| Government (%) | 16.1 | 15.3 | 20.0 | 14.2 | *χ^2^* (2, *N* = 314) = 1.36, Cramer’s V = .06 |
| *Hours Per Week [M(SD)]* | 9.7 (18.3) | 14.6 (25.6) | 6.6  (9.5) | 4.0 (1.5) | *F*(2,36) = 1.11,  *η_p_^2^* = **0.06** |
| Clinical practice—General intervention (non-forensic) (%) | 15.5 | 18.6 | 11.1 | 16.0 | *χ^2^* (2, *N* = 314) = 2.22, Cramer’s V = .08 |
| *Hours Per Week [M(SD)]* | 8.6 (6.4) | 10.7  (8.9) | 6.9  (2.9) | 7.2 (3.4) | *F*(2,38) = 1.53,  *η_p_^2^* = **0.08** |
| Institutional Service (%) | 14.8 | 21.2 | 18.9 | 4.7 | *χ^2^* (2, *N* = 314) = 13.42**, Cramer’s V = .21 |
| *Hours Per Week [M(SD)]* | 4.9 (6.5) | 5.4  (5.9) | 4.8  (7.9) | † | † |
| Research (Clinical) (%) | 12.9 | 18.6 | 15.6 | 4.7 | *χ^2^* (2, *N* = 314) = 10.23**, Cramer’s V = .18 |
| *Hours Per Week [M(SD)]* | 7.8 (6.7) | 7.1  (5.8) | 10.6  (8.1) | † | † |
| Training of law enforcement personnel, lawyers, judges (%) | 12.9 | 12.7 | 11.1 | 15.1 | *χ^2^* (2, *N* = 314) = 0.70, Cramer’s V = .05 |
| *Hours Per Week [M(SD)]* | 2.6 (2.6) | 3.4  (2.9) | 2.6  (3.1) | 1.7 (1.3) | *F*(2,28) = 1.15,  *η_p_^2^* = **0.08** |
| Supervision—Clinical and Support Personnel (%) | 12.3 | 16.9 | 14.4 | 5.7 | *χ^2^* (2, *N* = 314) = 7.02*, Cramer’s V = .15 |
| *Hours Per Week [M(SD)]* | 3.9 (3.9) | 3.7  (2.7) | 4.8  (5.6) | 2.4 (1.7) | *F*(2,34) = 0.68,  *η_p_^2^* = 0.04 |
| Health and mental health policy (%) | 7.3 | 8.5 | 10.0 | 3.8 | *χ^2^* (2, *N* = 314) = 3.14, Cramer’s V = .10 |
| *Hours Per Week [M(SD)]* | 2.6 (2.2) | 3.1  (2.4) | 1.5  (1.6) | † | † |
| Research (Non-Clinical) (%) | 6.0 | 7.6 | 7.8 | 2.8 | *χ^2^* (2, *N* = 314) = 2.92, Cramer’s V = .10 |
| *Hours Per Week [M(SD)]* | 10.7 (19.2) | 8.9  (6.2) | 2.6  (2.3) | † | † |
| Fact Witness (%) | 5.0 | 3.4 | 3.3 | 8.5 | *χ^2^* (2, *N* = 314) = 3.81, Cramer’s V = .11 |
| *Hours Per Week [M(SD)]* | 2.4 (3.2) | † | † | 2.4 (2.1) | † |
| Other (%)^a^ | 5.7 | 5.9 | 5.6 | 3.8 | † |
| *Hours Per Week [M(SD)]* | 24.1 (55.6) | 40.0 (78.5) | † | † | † |
| Professional Volunteering^b^ |  |  |  |  |  |
| *Hours Per Week [M(SD)]* | 1.6 (3.6) | 1.7  (4.8) | 1.3  (1.8) | 1.6 (2.2) | *F*(2,253) = 0.17,  *η_p_^2^* = 0.001 |

*Note.* “For what type of forensic psychology work activities did you receive income in 2018? (select all that apply)” and “Approximately how many hours per week (on average) did you spend on each of the following forensic psychology work activities in 2018?” and “Additionally, approximately how many hours per week (on average) did you spend on professional volunteering (e.g., non-service related committees, elected positions, peer review) related to forensic psychology in 2018?” Includes all eligible participants in the clinical group (n = 323). Professional Setting further includes clinical participants working in institutional settings only (n = 119), both institutional settings and private practice (n = 90), or private practice only (n = 107). Actual sample sizes reduced due to item non-response. Additional participants responded Mediation and Dispute Resolution (n = 9), Jury Consultation (*n* = 6), Judiciary (*n* = 5), Working within legislative committee staffs (*n* = 5), Industry (*n* = 4), and Legal Practice (*n* = 3). Additional participants did not provide sufficient information (*n* = 6). **Bold** indicates significant difference based on measure of association (i.e., at least a medium effect per *η^2^* > .06 and Cramer’s V > .30; Cohen, 1988, 1992). †Data not provided due to limited sample size.

^a^ “Other” responses included various activities related to training and supervision (*n* = 6) and other assessment (*n* = 4), among others.

^b^ Prevalence of professional volunteering was not collected.

**p* < .05. ***p* < .01. ****p* < .001.

**Supplemental Table 3.7**

***Professional Practice–Work Activities for Non-Clinical Forensic Psychology Participants***

|  | % | Hours Per Week *[M(SD)]* |
| --- | --- | --- |
| Research (non-clinical) | 50.0 | 16.9 (21.8) |
| Teaching | 44.1 | 17.9 (12.4) |
| Case Consultation | 35.3 | 10.1 (12.0) |
| Expert Witness^a^ | 35.3 | 5.4 (7.1) |
| Training of law enforcement personnel, lawyers, judges | 20.6 | 2.0 (1.6) |
| Institutional Service | 17.6 | 9.5 (11.8) |
| Clinical practice—Forensic assessment/evaluation^a^ | 14.7 | 27.5 (8.7) |
| Jury Consultation | 14.7 | 27.8 (23.7) |

*Note.* “For what type of forensic psychology work activities did you receive income in 2018? (select all that apply)” and “Approximately how many hours per week (on average) did you spend on each of the following forensic psychology work activities in 2018?” and “Additionally, approximately how many hours per week (on average) did you spend on professional volunteering (e.g., non-service related committees, elected positions, peer review) related to forensic psychology in 2018?” Includes all eligible participants in the non-clinical group (n = 35). Actual sample sizes reduced due to item non-response. Additional participants responded assessment of court functioning and administrative processes (*n* = 4); government (*n* = 3) and judiciary (*n* = 3); clinical practice—general assessment/evaluation (non-forensic), health and mental health policy, mediation and dispute resolution, and supervision—clinical trainees (all *n* = 2); clinical practice—forensic intervention, fact witness, industry, legal practice, research (clinical), and supervision—clinical and support personnel (all *n* = 1); and other (*n* = 4). Additional participants did not provide sufficient information (*n* = 1).

^a^ Generally among those with clinical degrees (i.e., in addition to non-clinical degrees and practice).

**Supplemental Table 3.8**

***Professional Practice–Test Use for Clinical Forensic Psychology Participants***

|  | Overall | Professional Setting | | | |
| --- | --- | --- | --- | --- | --- |
|  |  | Institution Only | Institution/ Private Practice | Private Practice Only | Difference |
| Forensic Assessment Instruments (Forensic Use) |  |  |  |  |  |
| Always | 19.4 | 13.1 | 15.2 | 28.7 | † |
| Most of the Time | 23.4 | 20.2 | 26.6 | 24.1 |  |
| About Half the Time | 16.7 | 21.4 | 19 | 10.3 |  |
| Sometimes | 35.3 | 40.5 | 35.4 | 31 |  |
| Never | 5.2 | 4.8 | 3.8 | 5.7 |  |
| Forensically Relevant Instruments (Forensic Use) |  |  |  |  |  |
| Always | 20.2 | 13.1 | 17.7 | 28.4 | *χ^2^* (8, *N* = 251) = 7.42, Cramer’s V = .12 |
| Most of the Time | 20.9 | 21.4 | 22.8 | 19.3 |  |
| About Half the Time | 15 | 16.7 | 16.5 | 12.5 |  |
| Sometimes | 35.6 | 39.3 | 36.7 | 31.8 |  |
| Never | 8.3 | 9.5 | 6.3 | 8 |  |
| Intelligence Tests |  |  |  |  |  |
| Forensic Use |  |  |  |  |  |
| *Always* | 9.0 | 4.7 | 6.3 | 15.9 | *χ^2^* (8, *N* = 253) = 21.58**, Cramer’s V = .21 |
| *Most of the Time* | 14.9 | 10.6 | 12.5 | 20.5 |  |
| *About Half the Time* | 9.8 | 7.1 | 7.5 | 14.8 |  |
| *Sometimes* | 49.0 | 54.1 | 55 | 39.8 |  |
| *Never* | 17.3 | 23.5 | 18.8 | 9.1 |  |
| General Use |  |  |  |  |  |
| *Always* | 15.9 | 8.0 | 0 | 32.1 | † |
| *Most of the Time* | 31.9 | 32.0 | 33.3 | 28.6 |  |
| *About Half the Time* | 11.6 | 20.0 | 6.7 | 7.1 |  |
| *Sometimes* | 21.7 | 24.0 | 26.7 | 17.9 |  |
| *Never* | 18.8 | 16.0 | 33.3 | 14.3 |  |
| Difference (Forensic vs. General) |  |  |  |  |  |
| *Always* | -6.9 | -3.3 | +6.3 | -16.2 |  |
| *Most of the Time* | -17 | -21.4 | -20.8 | -8.1 |  |
| *About Half the Time* | -1.8 | -12.9 | +0.8 | +7.7 |  |
| *Sometimes* | +27.3 | +30.1 | +28.3 | +21.9 |  |
| *Never* | -1.5 | +7.5 | -14.5 | -5.2 |  |
| Neuropsychological/Cognitive Tests |  |  |  |  |  |
| Forensic Use |  |  |  |  |  |
| *Always* | 3.2 | 0 | 3.9 | 5.8 | † |
| *Most of the Time* | 10.4 | 4.8 | 10.4 | 16.3 |  |
| *About Half the Time* | 9.6 | 7.1 | 7.8 | 12.8 |  |
| *Sometimes* | 55.4 | 65.5 | 61 | 41.9 |  |
| *Never* | 21.3 | 22.6 | 16.9 | 23.3 |  |
| General Use |  |  |  |  |  |
| *Always* | 7.5 | 4.0 | 0 | 14.8 | † |
| *Most of the Time* | 16.4 | 12.0 | 7.1 | 25.9 |  |
| *About Half the Time* | 19.4 | 24.0 | 7.1 | 18.5 |  |
| *Sometimes* | 37.3 | 44.0 | 50 | 25.9 |  |
| *Never* | 19.4 | 16.0 | 35.7 | 14.8 |  |
| Difference (Forensic vs. General) |  |  |  |  |  |
| *Always* | -4.3 | -4 | +3.9 | -9.0 |  |
| *Most of the Time* | -6 | -7.2 | +3.3 | -9.6 |  |
| *About Half the Time* | -9.8 | -16.9 | +0.7 | -5.7 |  |
| *Sometimes* | +18.1 | +21.5 | +11.0 | +16.0 |  |
| *Never* | +1.9 | +6.6 | -18.8 | +8.5 |  |
| Objective Personality Tests |  |  |  |  |  |
| Forensic Use |  |  |  |  |  |
| *Always* | 17.1 | 8.3 | 17.9 | 25 | *χ^2^* (8, *N* = 250) = 33.90***, Cramer’s V = .26 |
| *Most of the Time* | 22.6 | 9.5 | 25.6 | 31.8 |  |
| *About Half the Time* | 7.1 | 8.3 | 5.1 | 8 |  |
| *Sometimes* | 41.7 | 52.4 | 43.6 | 29.5 |  |
| *Never* | 11.5 | 21.4 | 7.7 | 5.7 |  |
| General Use |  |  |  |  |  |
| *Always* | 27.5 | 4.0 | 40 | 42.9 | † |
| *Most of the Time* | 43.5 | 44.0 | 40 | 42.9 |  |
| *About Half the Time* | 5.8 | 8.0 | 6.7 | 3.6 |  |
| *Sometimes* | 15.9 | 36.0 | 0 | 7.1 |  |
| *Never* | 7.2 | 8.0 | 13.3 | 3.6 |  |
| Difference (Forensic vs. General) |  |  |  |  |  |
| *Always* | -10.4 | +4.3 | -22.1 | -17.9 |  |
| *Most of the Time* | -20.9 | -34.5 | -14.4 | -11.1 |  |
| *About Half the Time* | +1.3 | +0.3 | -1.6 | +4.4 |  |
| *Sometimes* | +25.8 | +16.4 | +43.6 | +22.4 |  |
| *Never* | +4.3 | +13.4 | -5.6 | +2.1 |  |
| Projective Personality Tests |  |  |  |  |  |
| Forensic Use |  |  |  |  |  |
| *Always* | 0.8 | 1.3 | 0 | 1.1 | † |
| *Most of the Time* | 3.3 | 0 | 3.9 | 5.7 |  |
| *About Half the Time* | 2.9 | 1.3 | 2.6 | 4.6 |  |
| *Sometimes* | 14.3 | 8.8 | 17.1 | 14.9 |  |
| *Never* | 78.8 | 88.8 | 76.3 | 73.6 |  |
| General Use |  |  |  |  |  |
| *Always* | 2.9 | 0 | 0 | 7.1 | † |
| *Most of the Time* | 7.4 | 4.2 | 6.7 | 10.7 |  |
| *About Half the Time* | 4.4 | 4.2 | 13.3 | 0 |  |
| *Sometimes* | 23.5 | 33.3 | 6.7 | 21.4 |  |
| *Never* | 61.8 | 58.3 | 73.3 | 60.7 |  |
| Difference (Forensic vs. General) |  |  |  |  |  |
| *Always* | -2.1 | +1.3 | 0 | -6.0 |  |
| *Most of the Time* | -4.1 | -4.2 | -2.8 | -5.0 |  |
| *About Half the Time* | -1.5 | -2.9 | -10.7 | +4.6 |  |
| *Sometimes* | -9.2 | -24.5 | +10.4 | -6.5 |  |
| *Never* | +17 | +30.5 | +3.0 | +12.9 |  |

*Note.* “With what frequency did you use the following types of tests in your general assessments/evaluations (non-forensic) in 2018?” and “With what frequency did you use the following types of tests in your forensic assessments/evaluations in 2018?” Overall includes all eligible clinical forensic psychology participants reporting engagement in forensic assessment/evaluation (“Forensic Use”; *n* = 258) and general assessment/evaluation (non-forensic) (“General Use”; *n* = 69) (see Supplemental Table 3.6). Professional Setting further includes participants working in institutional settings only (Forensic *n* = 87; General *n* = 25), both institutional settings and private practice (Forensic *n* = 80; General *n* = 15), or private practice only (Forensic *n* = 89; General *n* = 28). Actual sample sizes reduced due to item non-response. Difference (Forensic vs. General) calculated by subtracting values for General Use from Forensic Use (i.e., positive value indicates increased use in forensic setting). **Bold** indicates significant difference based on measure of association (i.e., at least a medium effect per Cramer’s V > .30; Cohen, 1988, 1992). †Data not provided due to limited sample size.

**p* < .05. ***p* < .01. ****p* < .001.

**Supplemental Table 4.1**

***Financial Considerations—Overall Income and Income Sources***

|  | Overall | Clinical | | | | | Non-Clinical |
| --- | --- | --- | --- | --- | --- | --- | --- |
|  |  | Overall | Professional Setting | | | |  |
|  |  |  | Institution Only | Institution/ Private Practice | Private Practice Only | Difference |  |
| Overall |  |  |  |  |  |  |  |
| *Mdn* [*IQR*] | 116 [90-160] | 118 [94-161] | 100  [80-115] | 135  [100-184] | 160  [111-250] |  | 120  [79-150] |
| *M* (*SD*) | 146 (90) | 147 (91) | 100 (28) | 152 (69) | 202 (122) | *χ^2^* (2, *N* = 272) = 40.9***, *η_p_^2^* = **0.23** | 139 (90) |
| Forensic Psychology |  |  |  |  |  |  |  |
| *n* (%)^a^ | 280 (81) | 295 (93) | 107 (95) | 73 (91) | 79 (92) |  | 26 (90) |
| *Mdn* [*IQR*] | 100  [75-140] | 100  [75-140] | 86  [71-106] | 130  [96-165] | 117  [69-200] |  | 87  [37-115] |
| *M* (*SD*) | 119 (89) | 122 (89) | 88 (34) | 139 (70) | 151 (133) | *χ^2^* (2, *N* = 258) = 14.5***, *η_p_^2^* = **0.11** | 88 (74) |
| Other Psychology |  |  |  |  |  |  |  |
| *n* (%^a^) | 113 (33) | 105 (38) | 35 (31) | 24 (30) | 46 (53) |  | 9 (31) |
| *Mdn* [*IQR*] | 41 [16-79] | 41 [15-79] | 25 [10-72] | 35 [12-62] | 62 [25-100] |  | 43 [31-88] |
| *M* (*SD*) | 59 (71) | 59 (73) | 41 (34) | 40 (30) | 82 (92) | *χ^2^* (2, *N* = 104) = 4.5*,  *η_p_^2^* = **0.08** | 64 (50) |
| Other |  |  |  |  |  |  |  |
| *n* (%^a^) | 19 (5) | 18 (6) | 8 (7) | 2 (3)^b^ | 8 (9) |  | 1 (3)^c^ |
| *Mdn* [*IQR*] | 19 [5-90] | 15 [5-68] | 20 [5-84] | † | 7 [5-51] |  | † |
| *M* (*SD*) | 38 (46) | 35 (44) | 39 (51)^d^ | † | 25 (33)^d^ | † | † |

*Note.* Values are in thousands of USD rounded from participants’ pooled responses. Sample sizes include all eligible participants who reported working at least fulltime (i.e., 35 hours per week or more) in the overall (*n* = 346), clinical (n = 280), and non-clinical samples (*n* = 29). Professional Setting further includes clinical participants working in institutional settings only (n = 113), both institutional settings and private practice (n = 80), or private practice only (n = 86). Actual sample sizes reduced due to item non-response. IQR = Interquartile range. **Bold** indicates significant difference based on measure of association (i.e., at least a medium effect per *η_p_^2^* > .06). †Data not provided due to limited sample size.

^a^ Valid percentages based on within-group responses.

^b^ Values include $5,000 and $110,000.

^c^ Value is $100,000.

^d^ Values provided for descriptive purposes only.

**p* < .05. ***p* < .01. ****p* < .001.

**Supplemental Table 4.2**

***Financial Considerations—Overall Income, by Career Stage***

|  | Overall | Clinical | | | | | Non-Clinical |
| --- | --- | --- | --- | --- | --- | --- | --- |
|  |  | Overall | Professional Setting | | | |  |
|  |  |  | Institution Only | Institution/ Private Practice | Private Practice Only | Difference |  |
| Early Career (*n*) | 133 | 123 | 74 | 27 | 22 |  | 11 |
| *Mdn* [*IQR*] | 94 [76-114] | 95 [77-115] | 65 [75-105] | 110 [90-140] | 110 [79-163] |  | 70 [59-95] |
| *M* (*SD*) | 102 (45) | 105 (46) | 90 (25) | 130 (59) | 124 (62) | *F*(2,122) = 11.5***,  *η_p_^2^* = **0.16** | 76 (25) |
| Mid-Career (*n*) | 93 | 85 | 31 | 32 | 22 |  | 9 |
| *Mdn* [*IQR*] | 113 [109-177] | 135 [110-180] | 114 [100-135] | 135 [110-196] | 190 [126-315] |  | 125 [104-150] |
| *M* (*SD*) | 159 (88) | 133 (92) | 119 (26) | 156 (59) | 233 (138) | *F*(2,84) = 13.33***, *η_p_^2^* = **0.25** | 87 (44) |
| Senior Career (*n*) | 37 | 33 | 5 | 7 | 21 |  | 6 |
| *Mdn* [*IQR*] | 160 [128-198] | 155 [118-190] | 107 [96-127] | 160 [140-195] | 160 [128-225] |  | 163 [126-263] |
| *M* (*SD*) | 162 (94) | 113 (93) | 96 (33) | 169 (30) | 196 (105) | *F*(2,32) = 1.9, *η_p_^2^* = **0.12** | 191 (90) |
| Late-Senior Career (*n*) | 31 | 30 | 3 | 9 | 18 |  | 3 |
| *Mdn* [*IQR*] | 190 [130-350] | 185 [130-350] | † | 176 [120-225] | 247 [145-360] |  | † |
| *M* (*SD*) | 238 (131) | 233 (130) | † | 198 (123) | 266 (134) | † | † |

*Note.* Values are in thousands of USD rounded from participants’ pooled responses. Sample sizes include all eligible participants who reported working at least fulltime (i.e., 35 hours per week or more) in the overall (*n* = 346), clinical (n = 280), and non-clinical samples (*n* = 29). Professional Setting further includes clinical participants working in institutional settings only (n = 113), both institutional settings and private practice (n = 80), or private practice only (n = 86). Actual sample sizes reduced due to item non-response. Early Career = first 10 years since highest degree (first such degree if multiple); Mid-Career = 11-20 years; Senior Career = 21-30 years; Late-Senior Career = 31-50 years. IQR = Interquartile range. **Bold** indicates significant difference based on measure of association (i.e., at least a medium effect per *η_p_^2^* > .06). †Data not provided due to limited sample size.

**p* < .05. ***p* < .01. ****p* < .001.

**Supplemental Table 4.3**

***Financial Considerations—Overall Income, by Degree for Clinical Forensic Psychology Participants***

|  | Overall | Professional Setting | | | |
| --- | --- | --- | --- | --- | --- |
|  |  | Institution Only | Institution/ Private Practice | Private Practice Only | Difference |
| PhD (*n*) | 162 | 63 | 40 | 58 |  |
| *Mdn* [*IQR*] | 128 [95-171] | 100 [85-130] | 133 [100-178] | 166 [118-255] |  |
| *M* (*SD*) | 151 (91) | 105 (28) | 145 (74) | 201 (114) | *F*(2,160) = 21.70***, *η_p_^2^* = **0.22** |
| PsyD (*n*) | 96 | 44 | 31 | 21 |  |
| *Mdn* [*IQR*] | 114 [94-150] | 101 [77-112] | 140 [114-202] | 140 [103-238] |  |
| *M* (*SD*) | 137 (77) | 98 (23) | 163 (67) | 179 (118) | *F*(2,95) = 13.25***, *η_p_^2^* = **0.22** |
| Difference | *t*(256) = 1.25,  *d* = 0.16 | *t*(105) = 1.34,  *d* = 0.26 | *t*(69) = 1.05,  *d* = 0.25 | *t*(77) = 0.73,  *d* = 0.19 |  |

*Note.* Values are in thousands of USD rounded from participants’ pooled responses. Sample sizes include all eligible participants who reported working at least fulltime (i.e., 35 hours per week or more) in the clinical (n = 280) sample. Professional Setting further includes clinical participants working in institutional settings only (n = 113), both institutional settings and private practice (n = 80), or private practice only (n = 86). Actual sample sizes reduced due to item non-response. IQR = Interquartile range. **Bold** indicates significant difference based on measure of association (i.e., at least a medium effect per *d* > 0.5 or *η_p_^2^* > .06). †Data not provided due to limited sample size.

**p* < .05. ***p* < .01. ****p* < .001.

**Supplemental Table 4.4**

***Financial Considerations—Overall Income, by Completion of Postdoctoral Fellowship***

|  | Overall | Clinical | | | | | Non-Clinical |
| --- | --- | --- | --- | --- | --- | --- | --- |
|  |  | Overall | Professional Setting | | | |  |
|  |  |  | Institution Only | Institution/ Private Practice | Private Practice Only | Difference |  |
| Yes (*n*) | 147 | 146 | 61 | 35 | 50 |  | 3^a^ |
| *Mdn* [*IQR*] | 111 [90-172] | 111 [90-173] | 95 [79-110] | 150 [130-200] | 156 [107-256] |  | † |
| *M* (*SD*) | 147 (89) | 147 (89) | 97 (22) | 172 (84) | 192 (112) | *F*(2,145) = 22.7***,  *η_p_^2^* = **0.24** | † |
| No (*n*) | 138 | 119 | 52 | 38 | 29 |  | 22 |
| *Mdn* [*IQR*] | 120 [90-150] | 120 [95-150] | 103 [81-130] | 120 [99-164] | 162 [125-250] |  | 104 [71-150] |
| *M* (*SD*) | 140 (91) | 141 (90) | 103 (34) | 135 (50) | 218 (141) | *F*(2,118) = 20.1***,  *η_p_^2^* = **0.26** | 132 (91) |
| Difference | *t*(283) = 0.65, *d* = 0.08 | *t*(263) = 0.54, *d* = 0.07 | *t*(111) = 1.29, *d* = 0.24 | *t*(71) = 2.30*,  *d* = **0.54** | *t*(77) = 0.89,  *d* = 0.21 |  | † |

*Note.* Values are in thousands of USD rounded from participants’ pooled responses. Sample sizes include all eligible participants who reported working at least fulltime (i.e., 35 hours per week or more) in the overall (*n* = 346), clinical (n = 280), and non-clinical samples (*n* = 29). Professional Setting further includes clinical participants working in institutional settings only (n = 113), both institutional settings and private practice (n = 80), or private practice only (n = 86). Actual sample sizes reduced due to item non-response. IQR = Interquartile range. **Bold** indicates significant difference based on measure of association (i.e., at least a medium effect per *d* > 0.5 or *η_p_^2^* > .06). †Data not provided due to limited sample size.

^a^ Values include $100,000, $150,000, and $350,000.

**p* < .05. ***p* < .01. ****p* < .001.

**Supplemental Table 4.5**

***Financial Considerations—Overall Income, by Board Certification Status***

|  | Overall | Professional Setting | | | |
| --- | --- | --- | --- | --- | --- |
|  |  | Institution Only | Institution/ Private Practice | Private Practice Only | Difference |
| Not Boarded (*n*) | 185 | 90 | 47 | 48 |  |
| *Mdn* [*IQR*] | 110 [85-140] | 94 [77-114] | 130 [100-160] | 145 [101-180] |  |
| *M* (*SD*) | 104 (72) | 97 (29) | 144 (63) | 170 (106) | *F*(2,184) = 21.1***, *η_p_^2^* = **0.18** |
| Boarded—Any (*n*) | 80 | 19 | 26 | 35 |  |
| *Mdn* [*IQR*] | 150 [113-220] | 113 [102-130] | 155 [118-196] | 210 [135-340] |  |
| *M* (*SD*) | 189 (112) | 115 (199) | 169 (80) | 245 (132) | *F*(2,79) = 11.4***, *η_p_^2^* = **0.23** |
| Boarded—Any ABPP^a^ (n) | 74 | 19 | 26 | 29 |  |
| Mdn [IQR] | 149 [112-203] | 113 [102-130] | 155 [118-196] | 200 [135-345] |  |
| M (SD) | 185 (112) | 115 (20) | 169 (80) | 246 (140) | F(2,73) = 10.4***, ηp2 = 0.23 |
| Boarded—ABFP (*n*) | 62 | 16 | 23 | 23 |  |
| *Mdn* [*IQR*] | 144 [110-203] | 110 [101-118] | 148 [110-195] | 210 [150-350] |  |
| *M* (*SD*) | 185 (117) | 111 (183) | 166 (84) | 256 (145) | *F*(2,61) = 9.9***, *η_p_^2^* = **0.25** |

*Note.* Values are in thousands of USD rounded from participants’ pooled responses. Sample sizes include all eligible participants who reported working at least fulltime (i.e., 35 hours per week or more) in the clinical group (n = 280), including those reporting no board certification (*n* = 188), any board certification (generally; *n* = 94), any ABPP certification (*n* = 88), and ABFP certification (*n* = 64). Additional participants preferred to not respond regarding board certification status (*n* = 5) or did not provide sufficient information (*n* = 4). Professional Setting further includes clinical participants working at least fulltime in institutional settings only (*n* = 19), both institutional settings and private practice (*n* = 28), or private practice only (*n* = 36). Actual sample sizes reduced due to item non-response. Additional participants reported certification through the American Board of Professional Neuropsychology, American Board of Assessment Psychology, American College of Forensic Psychology, and National Board of Forensic Evaluators (all n = 1); these participants are not included in additional subgroup analyses due to limited sample size. Non-clinical participants not included due to lack of relevance for board certification within the profession. ABFP = American Board of Forensic Psychology. ABPP = American Board of Professional Psychology. IQR = Interquartile range. **Bold** indicates significant difference based on measure of association (i.e., at least a medium effect per *η_p_^2^* > .06; Cohen, 1988, 1992). †Data not provided due to limited sample size.

^a^ Specific ABPP-affiliated boards generally included the American Board of Clinical Psychology, American Board of Clinical Neuropsychology, American Board of Clinical Child & Adolescent Psychology, American Board of Counseling Psychology, American Board of Couple & Family Psychology, American Board of Geropsychology, American Board of School Psychology, and American Board of Professional Psychology (all n = 1).

**p* < .05. ***p* < .01. ****p* < .001.

**Supplemental Table 4.6**

***Financial Considerations—Overall Income, by Current Region and Area of Residence***

|  | Overall | Clinical | | | | Non-Clinical |
| --- | --- | --- | --- | --- | --- | --- |
|  |  | Overall | Professional Setting | | |  |
|  |  |  | Institution Only | Institution/ Private Practice | Private Practice Only |  |
| Current Region^a^ |  |  |  |  |  |  |
| New England (*n*) | 12 | 12 | 2 | 4 | 6 | 3 |
| *Mdn* [*IQR*] | 120 [93-179] | 120 [93-179] | † | 135 [88-183] | 135 [98-226] | † |
| Middle Atlantic (*n*) | 24 | 21 | 8 | 8 | 5 | 4 |
| *Mdn* [*IQR*] | 107 [91-150] | 110 [92-150] | 92 [84-102] | 144 [105-169] | 150 [79-198] | 95 [70-199] |
| South Atlantic (*n*) | 47 | 44 | 17 | 10 | 17 | 3 |
| *Mdn* [*IQR*] | 115 [85-140] | 113 [85-139] | 85 [72-106] | 133 [96-137] | 135 [110-288] | † |
| East North Central (*n*) | 24 | 20 | 10 | 5 | 5 | 4 |
| *Mdn* [*IQR*] | 112 [81-191] | 115 [85-191] | 98 [78-117] | 202 [93-223] | 156 [100-450] | 94 [50-327] |
| East South Central (*n*) | 4 | 4 | 1 | 0 | 3 | 0 |
| *Mdn* [*IQR*] | 128 [78-204] | 128 [78-204] | † | † | † | † |
| West North Central (*n*) | 20 | 20 | 13 | 1 | 6 | 0 |
| *Mdn* [*IQR*] | 108 [80-140] | 107 [80-140] | 96 [72-108] | † | 134 [120-214] | † |
| West South Central (*n*) | 15 | 15 | 5 | 5 | 5 | 1 |
| *Mdn* [*IQR*] | 130 [110-170] | 130 [110-170] | 121 [91-140] | 135 [105-165] | 170 [95-390] | † |
| Mountain (*n*) | 16 | 14 | 3 | 8 | 3 | 2 |
| *Mdn* [*IQR*] | 115 [83-150] | 115 [84-157] | † | 115 [96-162] | † | † |
| Pacific (*n*) | 53 | 53 | 22 | 15 | 16 | 0 |
| *Mdn* [*IQR*] | 130 [100-172] | 130 [100-172] | 108 [89-136] | 130 [101-180] | 166 [130-249] | † |
| Current Area^b^ |  |  |  |  |  |  |
| Large Metropolitan (*n*) | 71 | 65 | 22 | 19 | 24 | 8 |
| *Mdn* [*IQR*] | 135 [105-180] | 135 [103-180] | 100 [79-114] | 140 [114-190] | 180 [136-324] | 183 [139-321] |
| Metropolitan (*n*) | 59 | 56 | 20 | 17 | 19 | 4 |
| *Mdn* [*IQR*] | 130 [90-176] | 133 [91-178] | 102 [73-148] | 135 [98-189] | 160 [115-275] | 125 [83-145] |
| Medium-Sized Urban (*n*) | 48 | 44 | 17 | 15 | 12 | 4 |
| *Mdn* [*IQR*] | 112 [91-155] | 115 [94-159] | 95 [81-112] | 135 [101-180] | 163 [113-323] | 92 [60-136] |
| Small Urban (*n*) | 63 | 57 | 32 | 11 | 14 | 7 |
| *Mdn* [*IQR*] | 110 [85-138] | 110 [88-134] | 100 [82-115] | 130 [110-195] | 135 [108-195] | 100 [59-150] |
| Rural (*n*) | 45 | 40 | 18 | 10 | 12 | 7 |
| *Mdn* [*IQR*] | 108 [85-140] | 114 [90-146] | 104 [85-128] | 130 [91-161] | 144 [93-211] | 83 [62-108] |

*Note.* Values are in thousands of USD rounded from participants’ pooled responses. Sample sizes include all eligible participants who reported working at least fulltime (i.e., 35 hours per week or more) in the overall (*n* = 346), clinical (n = 280), and non-clinical samples (*n* = 29). Professional Setting further includes clinical participants working in institutional settings only (n = 113), both institutional settings and private practice (n = 80), or private practice only (n = 86). Actual sample sizes reduced due to item non-response. IQR = Interquartile range. †Data not provided due to limited sample size.

^a^ New England = Connecticut, Maine, Massachusetts, New Hampshire, Rhode Island and Vermont; Middle Atlantic = New Jersey, New York and Pennsylvania; South Atlantic = Delaware, District of Columbia, Florida, Georgia, Maryland, North Carolina, South Carolina, Virginia and West Virginia; East North Central = Illinois, Indiana, Michigan, Ohio and Wisconsin; East South Central = Alabama, Kentucky, Mississippi and Tennessee; West North Central = Iowa, Kansas, Minnesota, Missouri, Nebraska, North Dakota and South Dakota; West South Central = Arkansas, Louisiana, Oklahoma and Texas; Mountain = Arizona, Colorado, Idaho, Montana, Nevada, New Mexico, Utah and Wyoming; Pacific = Alaska, California, Hawaii, Oregon and Washington. Grouping is based on the primary area of current residence reported by participants (viz. ZIP codes) and appears to capture the experience of most participants given the limited number who lived across multiple geographical divisions. Additional participants did not provide sufficient information (*n* = 84).

^b^ Multiple responses allowed. Large Metropolitan = Population 1.5 million or more; Metropolitan = Population between 500,000 and 1.5 million; Medium-Sized Urban = Population between 200,000 and 500,000; Small Urban = Population between 50,000 and 200,000; Rural = Population 50,000 or less (Organisation for Economic Co-operation and Development, 2012). Additional participants responded Other (*n* = 3; all ‘Suburban’ or similar), preferred to not respond (*n* = 2), or did not provide sufficient information (*n* = 9).

**Supplemental Table 4.7**

***Financial Considerations—Incomes and Related Disparities, by Gender and Professional Setting***

|  | Overall | Clinical | | | | Non-Clinical |
| --- | --- | --- | --- | --- | --- | --- |
|  |  | Overall | Professional Setting | | |  |
|  |  |  | Institution Only | Institution/ Private Practice | Private Practice Only |  |
| Overall |  |  |  |  |  |  |
| Women (*n*) | 187 | 170 | 86 | 45 | 39 | 21 |
| *Mdn* [*IQR*] | 106 [85-135] | 106 [86-135] | 100 [81-114] | 130 [100-149] | 130 [100-172] | 108 [79-143] |
| Men (*n*) | 98 | 92 | 23 | 29 | 40 | 8 |
| *Mdn* [*IQR*] | 160 [115-241] | 160 [116-244] | 101 [84-140] | 175 [125-218] | 223 [150-350] | 163 [69-310] |
| Disparity | 0.66 | 0.66 | 0.99 | 0.74 | 0.58 | 0.66 |
| Forensic Psychology |  |  |  |  |  |  |
| Women (*n*) | 172 | 157 | 81 | 41 | 35 | 19 |
| *Mdn* [*IQR*] | 94 [70-110] | 95 [73-113] | 86 [70-105] | 103 [94-138] | 90 [35-135] | 70 [35-99] |
| Men (*n*) | 96 | 90 | 22 | 29 | 39 | 7 |
| *Mdn* [*IQR*] | 135 [90-200] | 135 [91-201] | 91 [75-136] | 160 [110-211] | 150 [95-280] | 150 [59-190] |
| Disparity | 0.70 | 0.70 | 0.95 | 0.64 | 0.60 | 0.47 |
| Other Psychology |  |  |  |  |  |  |
| Women (*n*) | 67 | 60 | 23 | 15 | 22 | 8 |
| *Mdn* [*IQR*] | 50 [17-82] | 50 [17-82] | 30 [6-78] | 34 [7-70] | 62 [37-90] | 46 [38-96] |
| Men (*n*) | 40 | 39 | 9 | 9 | 21 | 1 |
| *Mdn* [*IQR*] | 41 [13-75] | 41 [13-75] | 15 [10-43] | 41 [16-59] | 63 [22-120] | † |
| Disparity | 1.22 | 1.22 | 2.00 | 0.83 | 0.98 | † |
| Other |  |  |  |  |  |  |
| Women (*n*) | 10 | 9 | 5 | 2 | 2 | 1 |
| *Mdn* [*IQR*] | 55 [9-106] | 20 [8-108] | 20 [7-121] | † | † | † |
| Men (*n*) | 7 | 7 | 2 | 0 | 5 | 0 |
| *Mdn* [*IQR*] | 6 [5-20] | 6 [5-20] | † | - | 6 [6-34] | - |
| Disparity | 9.17 | 3.33 | † | † | † | † |

*Note.* Values are in thousands of USD rounded from participants’ pooled responses. Sample sizes include all eligible participants who reported working at least fulltime (i.e., 35 hours per week or more) in the overall (*n* = 346), clinical (n = 280), and non-clinical samples (*n* = 29). Professional Setting further includes clinical participants working in institutional settings only (n = 113), both institutional settings and private practice (n = 80), or private practice only (n = 86). Actual sample sizes reduced due to item non-response. Gender grouping based on binary gender identity variable (i.e., Female or Male, with additional “I prefer not to respond to this item”); results did not differ when compared to individual response options (i.e., Woman/Female and Man/Male, among other options). Disparity was determined by dividing median values for women by median values for men, and therefore represent relative differences standardized on a 1.00 scale. †Data not provided due to limited sample size.

**Supplemental Table 4.8**

***Financial Considerations—Incomes and Related Disparities, by Gender, Professional Setting, and Career Stage***

|  | Overall | Clinical | | | | Non-Clinical |
| --- | --- | --- | --- | --- | --- | --- |
|  |  | Overall | Professional Setting | | |  |
|  |  |  | Institution Only | Institution/ Private Practice | Private Practice Only |  |
| Early Career |  |  |  |  |  |  |
| Women (*n*) | 105 | 97 | 62 | 20 | 15 | 9 |
| *Mdn* [*IQR*] | 94 [77-111] | 95 [80-113] | 88 [76-106] | 101 [86-130] | 100 [80-160] | 74 [62-95] |
| Men (*n*) | 23 | 21 | 9 | 7 | 5 | 2 |
| *Mdn* [*IQR*] | 101 [72-150] | 110 [79-150] | 84 [71-98] | 140 [110-160] | 150 [87-226] | † |
| Disparity | 0.93 | 0.86 | 1.05 | 0.72 | 0.67 | † |
| Mid-Career |  |  |  |  |  |  |
| Women (*n*) | 59 | 53 | 21 | 20 | 12 | 7 |
| *Mdn* [*IQR*] | 130 [102-150] | 130 [101-150] | 114 [100-130] | 135 [103-169] | 153 [109-219] | 125 [108-150] |
| Men (*n*) | 30 | 28 | 10 | 10 | 8 | 2 |
| *Mdn* [*IQR*] | 155 [115-221] | 164 [116-224] | 129 [96-162] | 191 [131-231] | 290 [158-513] | † |
| Disparity | 0.84 | 0.79 | 0.88 | 0.71 | 0.53 | † |
| Senior Career |  |  |  |  |  |  |
| Women (*n*) | 12 | 10 | 1 | 3 | 6 | 4 |
| *Mdn* [*IQR*] | 145 [108-159] | 138 [105-156] | † | † | 130 [98-160] | † |
| Men (*n*) | 23 | 21 | 3 | 4 | 14 | 2 |
| *Mdn* [*IQR*] | 175 [135-250] | 162 [135-235] | † | † | 171 [135-329] | † |
| Disparity | 0.83 | 0.85 | † | † | 0.76 | † |
| Late Senior Career |  |  |  |  |  |  |
| Women (*n*) | 9 | 8 | 2 | 1 | 5 | 1 |
| *Mdn* [*IQR*] | 130 [128-278] | 130 [126-152] | † | † | 130 [113-378] | † |
| Men (*n*) | 22 | 22 | 1 | 8 | 13 | 2 |
| *Mdn* [*IQR*] | 235 [145-350] | 235 [145-350] | † | 183 [115-238] | 270 [203-370] | † |
| Disparity | 0.55 | 0.55 | † | † | 0.48 | † |

*Note.* Values are in thousands of USD rounded from participants’ pooled responses. Sample sizes include all eligible participants who reported working at least fulltime (i.e., 35 hours per week or more) in the overall (*n* = 346), clinical (n = 280), and non-clinical samples (*n* = 29). Professional Setting further includes clinical participants working in institutional settings only (n = 113), both institutional settings and private practice (n = 80), or private practice only (n = 86). Actual sample sizes reduced due to item non-response. Gender grouping based on binary gender identity variable (i.e., Female or Male, with additional “I prefer not to respond to this item”); results did not differ when compared to individual response options (i.e., Woman/Female and Man/Male, among other options). Early Career = first 10 years since highest degree (first such degree if multiple); Mid-Career = 11-20 years; Senior Career = 21-30 years; Late-Senior Career = 31-50 years. IQR = Interquartile range. Disparity was determined by dividing median values for women by median values for men, and therefore represent relative differences standardized on a 1.00 scale. †Data not provided due to limited sample size.

**Supplemental Table 4.9**

***Financial Consideration—Employment Benefits***

|  | Overall | Clinical | | | | | Non-Clinical |
| --- | --- | --- | --- | --- | --- | --- | --- |
|  |  | Overall | Professional Setting | | | |  |
|  |  |  | Institution Only | Institution/ Private Practice | Private Practice Only | Difference |  |
| Health Insurance | 71.0 | 69.6 | 95.6 | 83.8 | 23.3 | χ^2^ (2, N = 279) = 131.6***, Cramer's V = **0.69** | 86.2 |
| Retirement Plan | 69.0 | 67.9 | 94.7 | 86.3 | 16.3 | χ^2^ (2, N = 279) = 155.2***, Cramer's V = **0.75** | 79.3 |
| Insurance—Dental | 66.3 | 64.6 | 92.9 | 77.5 | 16.3 | χ^2^ (2, N = 279) = 133.7***, Cramer's V = **0.69** | 79.3 |
| Insurance—Vision | 58.4 | 56.8 | 85.0 | 68.8 | 9.3 | χ^2^ (2, N = 279) = 120.4***, Cramer's V = **0.66** | 75.9 |
| Insurance—Life | 56.8 | 55.4 | 85.0 | 60.0 | 12.8 | χ^2^ (2, N = 279) = 103.9***, Cramer's V = **0.61** | 65.5 |
| Insurance—Short-term Disability | 45.9 | 45.0 | 69.0 | 50.0 | 9.3 | χ^2^ (2, N = 279) = 71.4***, Cramer's V = **0.51** | 58.6 |
| Insurance—Long-term Disability | 39.6 | 37.9 | 58.4 | 38.8 | 10.5 | χ^2^ (2, N = 279) = 47.7***, Cramer's V = **0.41** | 62.1 |
| Flexible Spending Accounts (FSAs) | 38.6 | 37.5 | 61.9 | 41.3 | 2.3 | χ^2^ (2, N = 279) = 74.6***, Cramer's V = **0.52** | 48.3 |
| Health Savings Accounts (HSAs) | 37.3 | 36.1 | 54.9 | 41.3 | 7.0 | χ^2^ (2, N = 279) = 49.7***, Cramer's V = **0.42** | 51.7 |
| Paid leave—Other medical (personal) | 32.0 | 31.4 | 44.2 | 43.8 | 3.5 | χ^2^ (2, N = 279) = 45.3***, Cramer's V = **0.40** | 37.9 |
| Paid leave—Other medical (family) | 27.1 | 27.1 | 43.4 | 31.3 | 2.3 | χ^2^ (2, N = 279) = 42.4***, Cramer's V = **0.39** | 20.7 |
| Paid leave—Maternity | 24.1 | 22.9 | 38.9 | 22.5 | 2.3 | χ^2^ (2, N = 279) = 37.0***, Cramer's V = **0.36** | 37.9 |
| Paid leave—Paternity | 8.9 | 8.6 | 11.5 | 13.8 | 0 | χ^2^ (2, N = 279) = 12.0**, Cramer's V = 0.21 | 10.3 |
| Childcare benefits | 8.6 | 7.9 | 12.4 | 8.8 | 1.2 | χ^2^ (2, N = 279) = 8.6*, Cramer's V = 0.18 | 13.8 |
| Other^a^ | 5.9 | 6.1 | 2.7 | 6.3 | 10.5 | χ^2^ (2, N = 279) = 5.2, Cramer's V = 0.14 | 3.4 |
| Flexible schedule | 54.5 | 53.2 | 60.2 | 62.5 | 36.0 | χ^2^ (2, N = 279) = 15.1***, Cramer's V = 0.23 | 69.0 |
| Telecommuting | 25.4 | 24.3 | 25.7 | 36.3 | 11.6 | χ^2^ (2, N = 279) = 13.8**, Cramer's V = 0.22 | 34.5 |

*Note.* Valid percentages based on within-group responses. Sample sizes include all eligible participants who reported working at least fulltime (i.e., 35 hours per week or more) in the overall (*n* = 346), clinical (n = 280), and non-clinical samples (*n* = 29). Professional Setting further includes clinical participants working in institutional settings only (n = 113), both institutional settings and private practice (n = 80), or private practice only (n = 86). Actual sample sizes reduced due to item non-response. **Bold** indicates significant difference based on measure of association (i.e., at least a medium effect per Cramer’s V > .30; Cohen, 1988, 1992).

^a^ Mostly used to indicate “none” or similar due to self-employment in private practice (*n* = 9) and paid time off (*n* = 4), among others.

**p* < .05. ***p* < .01. ****p* < .001.

**Supplemental Table 4.10**

***Financial Considerations—Retirement***

|  | Overall | Clinical | | | | | Non-Clinical |
| --- | --- | --- | --- | --- | --- | --- | --- |
|  |  |  | Professional Setting | | | |  |
|  |  | Overall | Institution Only | Institution/ Private Practice | Private Practice Only | Difference |  |
| Expected Age of Retirement (years) |  |  |  |  |  |  |  |
| *Mdn* [*IQR*] | 70 [65-75] | 70 [65-75] | 65 [62-70] | 70 [65-75] | 70 [66-80] |  | 70 [65-75] |
| *M* (*SD*) | 70.1 (12.8) | 69.8 (12.7) | 66.1 (11.1) | 68.7 (13.1) | 74.5 (12.5) | χ^2^ (2, N = 323) = 27.7***, *η2* = **0.08** | 72.5 (16.2) |
| Factor Affecting Retirement Decision^a^ |  |  |  |  |  |  |  |
| Financial Status |  |  |  |  |  |  |  |
| *Rank 1* | 51.2 | 50.5 | 53.3 | 57.0 | 42.2 | χ^2^ (8, N = 323) = 17.63*, Cramer's V = 0.18 | 55.2 |
| *Rank 2* | 20.5 | 21.4 | 25.2 | 20.3 | 17.8 |  | 10.3 |
| *Rank 3* | 13.2 | 13.9 | 12.1 | 7.6 | 21.1 |  | 13.8 |
| *Rank 4* | 14.5 | 13.5 | 9.3 | 12.7 | 18.9 |  | 20.7 |
| *Rank 5* | 0.7 | 0.7 | 0 | 2.5 | 0 |  | 0 |
| *M* (*SD*)^b^ | 1.9 (1.1) | 1.9 (1.1) | 1.8 (1.0) | 1.8 (1.2) | 2.2 (1.2) |  | 2.0 (1.3) |
| Personal Health |  |  |  |  |  |  |  |
| *Rank 1* | 21.8 | 22.1 | 21.5 | 16.5 | 26.7 | χ^2^ (8, N = 351) = 7.13, Cramer's V = 0.11 | 20.7 |
| *Rank 2* | 37.3 | 37.0 | 35.5 | 38.0 | 38.9 |  | 41.4 |
| *Rank 3* | 23.4 | 22.8 | 24.3 | 21.5 | 22.2 |  | 27.6 |
| *Rank 4* | 14.5 | 14.9 | 16.8 | 19.0 | 8.9 |  | 10.3 |
| *Rank 5* | 3.0 | 3.2 | 1.9 | 5.1 | 3.3 |  | 0 |
| *M* (*SD*)^b^ | 2.4 (1.1) | 2.4 (1.1) | 2.4 (1.1) | 2.6 (1.1) | 2.3 (1.1) |  | 2.3 (0.9) |
| Quality of Work Life |  |  |  |  |  |  |  |
| *Rank 1* | 15.2 | 14.9 | 8.4 | 20.3 | 17.8 | χ^2^ (8, N = 323) = 11.58, Cramer's V = 0.14 | 17.2 |
| *Rank 2* | 17.2 | 16.4 | 14.0 | 20.3 | 15.6 |  | 24.1 |
| *Rank 3* | 31.7 | 31.7 | 32.7 | 29.1 | 33.3 |  | 34.5 |
| *Rank 4* | 25.4 | 26.3 | 29.9 | 25.3 | 23.3 |  | 17.2 |
| *Rank 5* | 10.6 | 10.7 | 15.0 | 5.1 | 10.0 |  | 6.9 |
| *M* (*SD*)^b^ | 2.9 (1.2) | 3.0 (1.2) | 3.3 (1.1) | 2.8 (1.2) | 2.9 (1.2) |  | 2.7 (1.2) |
| Family Considerations |  |  |  |  |  |  |  |
| *Rank 1* | 11.2 | 11.7 | 15.9 | 6.3 | 12.2 | χ^2^ (8, N = 323) = 13.72, Cramer's V = 0.16 | 6.9 |
| *Rank 2* | 18.2 | 18.5 | 15.9 | 16.5 | 23.3 |  | 17.2 |
| *Rank 3* | 25.1 | 24.6 | 25.2 | 35.4 | 14.4 |  | 24.1 |
| *Rank 4* | 35.0 | 34.2 | 31.8 | 31.6 | 38.9 |  | 48.3 |
| *Rank 5* | 10.6 | 11.0 | 11.2 | 10.1 | 11.1 |  | 3.4 |
| *M* (*SD*)^b^ | 3.2 (1.2) | 3.1 (1.2) | 3.1 (1.3) | 3.2 (1.0) | 3.1 (1.2) |  | 3.2 (1.0) |
| Continued Availability of Relevant Government Benefits |  |  |  |  |  |  |  |
| *Rank 1* | 0.7 | 0.7 | 0.9 | 0.0 | 1.1 | χ^2^ (8, N = 323) = 4.19, Cramer's V = 0.09 | 0 |
| *Rank 2* | 6.9 | 6.8 | 9.3 | 5.1 | 4.4 |  | 6.9 |
| *Rank 3* | 6.6 | 7.1 | 5.6 | 6.3 | 8.9 |  | 0.0 |
| *Rank 4* | 10.6 | 11.0 | 12.1 | 11.4 | 10.0 |  | 3.4 |
| *Rank 5* | 75.2 | 74.4 | 72.0 | 77.2 | 75.6 |  | 89.7 |
| *M* (*SD*)^b^ | 4.5 (0.9) | 4.5 (0.9) | 4.5 (1.0) | 4.6 (0.8) | 4.5 (0.9) |  | 4.8 (0.8) |

*Note.* Overall includes all eligible participants (n = 323). Professional Setting includes participants working in institutional settings only (n = 119), both institutional settings and private practice (n = 90), or private practice only (n = 107). Actual sample sizes reduced due to item non-response. IQR = Interquartile range. **Bold** indicates significant difference based on measure of association (i.e., at least a medium effect per *η^2^* > .06 and Cramer’s V > .30; Cohen, 1988, 1992).

^a^ “How would you rank the following factors in terms of their importance to determining the timing of your retirement?”

^b^ Mean and standard deviation of ordinal rankings (1-5) for descriptive purposes only.

**p* < .05. ***p* < .01. ****p* < .001.

**Supplemental Table 5.1**

***Wellbeing--Satisfaction Intercorrelations***

|  | *n* | *M* | *SD* | 1 | 2 | 3 |
| --- | --- | --- | --- | --- | --- | --- |
| Overall |  |  |  |  |  |  |
| 1. Work Activities | 327 | 78.3 | 16.2 | - |  |  |
| 1. Income | 324 | 71.8 | 22.3 | **.34***** | - |  |
| 1. Work-Life | 325 | 64.4 | 23.9 | **.42***** | .27*** | - |
| Clinical |  |  |  |  |  |  |
| 1. Work | 301 | 78.1 | 16.5 | - |  |  |
| 1. Income | 298 | 71.4 | 22.5 | **.34***** | - |  |
| 1. Work-Life | 300 | 63.9 | 23.9 | **.40***** | .27*** | - |
| Non-Clinical |  |  |  |  |  |  |
| 1. Work | 33 | 81.2 | 11.8 | - |  |  |
| 1. Income | 33 | 76.4 | 18.8 | **.37*** | - |  |
| 1. Work-Life | 32 | 70.3 | 24.3 | **.65***** | **.39*** | - |

*Note.* Two-tailed. **Bold** indicates significant difference based on measure of association (i.e., at least a medium effect per *r* > 0.30; Cohen, 1988, 1992).

**p* < .05. ***p* < .01. ****p* < .001.

**Supplemental Table 5.2**

***Wellbeing—Satisfaction***

|  | Overall | Clinical | | | | | Non-Clinical |
| --- | --- | --- | --- | --- | --- | --- | --- |
|  |  |  | Professional Setting | | | |  |
|  |  | Overall | Institution Only | Institution/ Private Practice | Private Practice Only | Differences |  |
| Overall^a^ |  |  |  |  |  |  |  |
| Scale [*M* (*SD*)]^b^ | 71 (16) | 71 (16) | 68 (16) | 70 (14) | 74 (18) | *F*(2,299) = 4.28*,  *η_p_^2^* = 0.03 | 76 (15) |
| Work Activities |  |  |  |  |  |  |  |
| Scale [*M* (*SD*)]^b^ | 78 (16) | 78 (17) | 75 (18) | 77 (14) | 83 (16) | *F*(2,299) = 6.30**,  *η_p_^2^* = 0.04 | 81 (12) |
| Categorical (*%*)^c^ |  |  |  |  |  |  |  |
| *Completely dissatisfied* | 5 (1.5) | 4 (1.3) | 0 (0.0) | 3 (3.4) | 1 (1.0) | χ^2^ (8, N = 323) = 18.11*, Cramer's V = 0.17 | 1 (3.0) |
| *Somewhat dissatisfied* | 20 (6.0) | 19 (6.1) | 10 (8.6) | 4 (4.6) | 5 (4.9) |  | 1 (3.0) |
| *Neither satisfied nor dissatisfied* | 7 (2.1) | 7 (2.3) | 3 (2.6) | 2 (2.3) | 2 (1.9) |  | 0 (0.0) |
| *Somewhat*  *satisfied* | 168 (50.1) | 156 (50.5) | 67 (57.8) | 47 (54.0) | 40 (38.8) |  | 17 (51.5) |
| *Completely satisfied* | 135 (40.3) | 123 (39.8) | 36 (31.0) | 31 (35.6) | 55 (53.4) |  | 14 (42.4) |
| Income |  |  |  |  |  |  |  |
| Scale [*M* (*SD*)]^b^ | 72 (22) | 71 (23) | 66 (24) | 72 (20) | 77 (22) | *F*(2,296) = 6.61**,  *η_p_^2^* = 0.04 | 76 (19) |
| Categorical (*%*)^c^ |  |  |  |  |  |  |  |
| *Completely dissatisfied* | 17 (5.1) | 15 (4.9) | 7 (6.0) | 3 (3.4) | 5 (4.9) | χ^2^ (8, N = 323) = 18.11*, Cramer's V = 0.17 | 2 (6.1) |
| *Somewhat dissatisfied* | 40 (12.0) | 39 (12.7) | 21 (18.1) | 8 (9.2) | 10 (9.8) |  | 1 (3.0) |
| *Neither satisfied nor dissatisfied* | 20 (6.0) | 19 (6.2) | 8 (6.9) | 4 (4.6) | 6 (5.9) |  | 1 (3.0) |
| *Somewhat*  *satisfied* | 149 (44.7) | 135 (44.0) | 52 (44.8) | 48 (55.2) | 35 (34.3) |  | 18 (54.5) |
| *Completely satisfied* | 107 (32.1) | 99 (32.2) | 28 (24.1) | 24 (27.6) | 46 (45.1) |  | 11 (33.3) |
| Work-Life |  |  |  |  |  |  |  |
| Scale [*M* (*SD*)]^b^ | 64 (24) | 64 (24) | 65 (24) | 62 (22) | 65 (25) | *F*(2,298) = 0.55,  *η_p_^2^* = 0.004 | 70 (24) |
| Categorical (*%*)^c^ |  |  |  |  |  |  |  |
| *Completely dissatisfied* | 19 (5.7) | 16 (5.2) | 5 (4.3) | 4 (4.5) | 7 (6.9) | χ^2^ (8, N = 323) = 5.51, Cramer's V = 0.10 | 3 (9.4) |
| *Somewhat dissatisfied* | 73 (22.0) | 68 (22.2) | 27 (23.5) | 22 (25.0) | 19 (18.8) |  | 8 (25.0) |
| *Neither satisfied nor dissatisfied* | 34 (10.2) | 31 (10.1) | 11 (9.6) | 12 (13.6) | 7 (6.9) |  | 3 (9.4) |
| *Somewhat*  *satisfied* | 132 (39.8) | 127 (41.5) | 45 (39.1) | 35 (39.8) | 46 (45.5) |  | 7 (21.9) |
| *Completely satisfied* | 74 (22.3) | 64 (20.9) | 27 (23.5) | 15 (17.0) | 22 (21.8) |  | 11 (34.4) |

*Note.* Sample sizes generally include all eligible participants in the overall (*n* = 351), clinical (n = 323), and non-clinical samples (*n* = 35). Professional Setting further includes clinical participants working in institutional settings only (*n* = 119), both institutional settings and private practice (*n* = 90), or private practice only (*n* = 107). Actual sample sizes reduced due to item non-response. **Bold** indicates significant difference based on measure of association (i.e., at least a medium effect per *η_p_^2^* > .06 and Cramer’s V > .30; Cohen, 1988, 1992).

^a^ Based on combined satisfaction variable (i.e., mean of work activities, income, work-life; see Supplemental Table 5.1).

^b^ “How would you quantify your  level of satisfaction or dissatisfaction in the following areas for 2018?” Based on a sliding scale from 0 “Completely Dissatisfied” to 100 “Completely Satisfied” with additional “No Opinion” option.

^c^ “Which of the following would best describe your level of satisfaction or dissatisfaction in the following areas for 2018?”

**p* < .05. ***p* < .01. ****p* < .001.

**Supplemental Table 5.3**

***Wellbeing—Satisfaction, by Gender, Career Stage, Degree Type, Postdoctoral Fellowship, and Board Certification***

|  | Overall | Clinical | | | | | Non-Clinical |
| --- | --- | --- | --- | --- | --- | --- | --- |
|  |  |  | Professional Setting | | | |  |
|  |  | Overall | Institution Only | Institution/ Private Practice | Private Practice Only | Differences |  |
| Gender |  |  |  |  |  |  |  |
| *Women* | 70 (16) | 69 (16) | 69 (14) | 68 (14) | 70 (19) | *F*(2,187) = 0.30,  *η_p_^2^* = .003 | 76 (15) |
| *Men* | 74 (17) | 74 (17) | 63 (19) | 75 (12) | 79 (16) | *F*(2,107) = 8.02***,  *η_p_^2^* = **.13** | 77 (15) |
| *Difference* | *t*(321) = 2.51**, *d* = 0.29 | *t*(295) = 2.70**, *d* = 0.33 | *t*(111) = 1.63, *d* = 0.38 | *t*(81) = 2.53**, *d* = **0.57** | *t*(98) = 2.68**, *d* = **0.54** |  | *t*(31) = 0.15, *d* = 0.06 |
| Career stage |  |  |  |  |  |  |  |
| *Early Career* | 69 (14) | 69 (14) | 70 (14) | 70 (14) | 68 (16) | *F*(2,124) = 0.11,  *η_p_^2^* = .002 | 70 (18) |
| *Mid-Career* | 71 (13) | 71 (13) | 67 (14) | 72 (12) | 75 (14) | *F*(2,87) = 2.17,  *η_p_^2^* = .05 | 76 (12) |
| *Senior Career* | 70 (23) | 68 (23) | † | 61 (20) | 73 (21) | † | 78 (12) |
| *Late Senior Career* | 77 (18) | 77 (18) | † | 76 (12) | 81 (18) | † | 87 (13) |
| *Difference* | *F*(3,324) = 3.11*,  *η_p_^2^* = .03 | *F*(3,298) = 3.04*,  *η_p_^2^* = .03 | † | *F*(3,83) = 2.68,  *η_p_^2^* = **.09** | *F*(3,99) = 2.21,  *η_p_^2^* = **.07** |  | † |
| Degree Type |  |  |  |  |  |  |  |
| *PhD* | - | 70 (16) | 67 (16) | 70 (15) | 73 (18) | *F*(2,180) = 2.56,  *η_p_^2^* = .03 | - |
| *PsyD* | - | 72 (15) | 72 (15) | 71 (14) | 76 (17) | *F*(2,100) = 0.95,  *η_p_^2^* = .02 | - |
| *Difference* | - | *t*(282) = 1.00, *d* = 0.12 | *t*(106) = 1.50, *d* = 0.29 | *t*(78) = 0.22, *d* = 0.05 | *t*(92) = 0.65, *d* = 0.15 |  | - |
| Postdoctoral Fellowship |  |  |  |  |  |  |  |
| *Yes* | 72 (15) | 71 (15) | 70 (14) | 70 (16) | 74 (16) | *F*(2,156) = 1.50,  *η_p_^2^* = .02 | † |
| *No* | 71 (17) | 70 (17) | 66 (17) | 72 (12) | 75 (20) | *F*(2,134) = 3.60*,  *η_p_^2^* = .05 | 74 (16) |
| *Difference* | *t*(312) = 0.39, *d* = .04 | *t*(291) = 0.55, *d* = 0.06 | *t*(112) = 1.36, *d* = 0.26 | *t*(80) = 0.55, *d* = 0.12 | *t*(94) = 0.29, *d* = 0.06 |  | † |
| Board Certification |  |  |  |  |  |  |  |
| *Any board* |  |  |  |  |  |  |  |
| *No* | - | 70 (16) | 69 (14) | 71 (14) | 71 (20) | *F*(2,201) = 0.23,  *η_p_^2^* = .002 | - |
| *Yes* | - | 73 (17) | 61 (21) | 71 (14) | 80 (12) | *F*(2,88) = 11.44***,  *η_p_^2^* = **.21** | - |
| *Difference* | *-* | *t*(289) = 1.40, *d* = 0.18 | *t*(108) = 2.13*, *d* = **0.53** | *t*(79) = 0.02, *d* = 0.004 | *t*(98) = 2.73**, *d* = **0.56** |  | - |
| *ABPP* |  |  |  |  |  |  |  |
| *No* | - | 70 (16) | 69 (14) | 71 (14) | 71 (20) | *F*(2,205) = 0.39,  *η_p_^2^* = .004 | - |
| *Yes* | - | 73 (17) | 61 (21) | 71 (14) | 81 (12) | *F*(2,83) = 11.41***,  *η_p_^2^* = **.22** | - |
| *Difference* | - | *t*(288) = 1.19, *d* = 0.15 | *t*(108) = 2.12*, *d* = **0.53** | *t*(79) = 0.02, *d* < 0.01 | *t*(97) = 2.65**, *d* = **0.56** |  | - |
| *ABFP* |  |  |  |  |  |  |  |
| *No* |  | 70 (16) | 69 (14) | 70 (15) | 72 (20) | *F*(2,225) = 0.65,  *η_p_^2^* = .006 |  |
| *Yes* | - | 73 (16) | 62 (22) | 71 (12) | 82 (10) | *F*(2,69) = 9.65***,  *η_p_^2^* = **.22** | - |
| *Difference* | - | *t*(296) = 1.33, *d* = 0.18 | *t*(112) = 1.54, *d* = 0.41 | *t*(80) = 0.11, *d* = 0.03 | *t*(98) = 2.50**, *d* = **0.56** |  |  |

*Note.* Based on *M* and *SD* for combined satisfaction variable (i.e., mean of work activities, income, work-life; see Supplemental Table 5.1). Sample sizes generally include all eligible participants in the overall (*n* = 351), clinical (n = 323), and non-clinical samples (*n* = 35). Professional Setting further includes clinical participants working in institutional settings only (*n* = 119), both institutional settings and private practice (*n* = 90), or private practice only (*n* = 107). Actual sample sizes reduced due to item non-response. Gender grouping based on binary gender identity variable (i.e., Female or Male, with additional “I prefer not to respond to this item”); results did not differ when compared to non-binary response options (i.e., individual items for Woman/Female and Man/Male, among other options). Early Career = first 10 years since highest degree (first such degree if multiple); Mid-Career = 11-20 years; Senior Career = 21-30 years; Late-Senior Career = 31-50 years. ABFP = American Board of Forensic Psychology. **Bold** indicates significant difference based on measure of association (i.e., at least a medium effect per *d* > 0.50 or *η_p_^2^* > .06). †Data not provided due to limited sample size.

**p* < .05. ***p* < .01. ****p* < .001.

**Supplemental Table 5.4**

***Wellbeing—******Obstacles to Satisfaction***

|  | Overall | Clinical | | | | | Non-Clinical |
| --- | --- | --- | --- | --- | --- | --- | --- |
|  |  | Overall |  |  |  |  |  |
|  |  |  | Institution Only | Institution/ Private Practice | Private Practice Only | Differences |  |
| Work Activities (%) |  |  |  |  |  |  |  |
| *Work environment—Personnel* | 37.5 | 39.4 | 53.4 | 47.7 | 17.5 | χ^2^ (2, N = 323) = 32.77*, Cramer's V = **0.33** | 18.2 |
| *Work Activities* | 34.5 | 35.5 | 45.7 | 29.5 | 30.1 | χ^2^ (2, N = 323) = 7.89*, Cramer's V = 0.16 | 27.3 |
| *Family Life* | 23.5 | 23.5 | 14.7 | 27.3 | 30.1 | χ^2^ (2, N = 323) = 8.25*, Cramer's V = 0.16 | 24.2 |
| *Work environment—Physical* | 21.1 | 21.6 | 29.3 | 21.6 | 13.6 | χ^2^ (2, N = 323) = 7.9*, Cramer's V = 0.16 | 12.1 |
| *Personal Life* | 17.3 | 17.4 | 10.3 | 14.8 | 26.2 | χ^2^ (2, N = 323) = 10.18*, Cramer's V = 0.18 | 15.2 |
| Income (%) |  |  |  |  |  |  |  |
| *Work Activities* | 22.6 | 22.6 | 15.5 | 25.0 | 28.2 | χ^2^ (2, N = 323) = 5.45, Cramer's V = 0.13 | 24.2 |
| *Work environment—Personnel* | 20.8 | 21.6 | 27.6 | 26.1 | 10.7 | χ^2^ (2, N = 323) = 10.81*, Cramer's V = 0.19 | 9.1 |
| *Family Life* | 17.9 | 19.0 | 12.1 | 22.7 | 23.3 | χ^2^ (2, N = 323) = 5.68, Cramer's V = 0.14 | 12.1 |
| *Personal Life* | 11.6 | 11.9 | 8.6 | 6.8 | 18.4 | χ^2^ (2, N = 323) = 7.78*, Cramer's V = 0.16 | 9.1 |
| *Work environment—Physical* | 6.0 | 6.1 | 10.3 | 5.7 | 1.9 | χ^2^ (2, N = 323) = 6.69*, Cramer's V = 0.15 | 3.0 |
| Work-Life (%) |  |  |  |  |  |  |  |
| *Work Activities* | 43.8 | 43.5 | 44.8 | 44.3 | 41.7 | χ^2^ (2, N = 323) = 0.23, Cramer's V = 0.03 | 42.4 |
| *Work environment—Personnel* | 25.0 | 26.1 | 31.0 | 31.8 | 15.5 | χ^2^ (2, N = 323) = 8.93*, Cramer's V = 0.17 | 9.1 |
| *Personal Life* | 21.1 | 21.3 | 22.4 | 18.2 | 21.4 | χ^2^ (2, N = 323) = 0.57, Cramer's V = 0.04 | 21.2 |
| *Family Life* | 20.2 | 20.6 | 16.4 | 27.3 | 19.4 | χ^2^ (2, N = 323) = 3.76, Cramer's V = 0.11 | 21.2 |
| *Work environment—Physical* | 14.0 | 15.2 | 24.1 | 12.5 | 7.8 | χ^2^ (2, N = 323= 12.03*, Cramer's V = 0.20 | 0 |

*Note.* “Did any of the following create obstacles to attaining greater satisfaction with your [work activities/income/work-life balance] in 2018? (select all that apply)” Sample sizes generally include all eligible participants in the overall (*n* = 351), clinical (n = 323), and non-clinical samples (*n* = 35). Professional Setting further includes clinical participants working in institutional settings only (*n* = 119), both institutional settings and private practice (*n* = 90), or private practice only (*n* = 107). Actual sample sizes reduced due to item non-response. **Bold** indicates significant difference based on measure of association (i.e., at least a medium effect per Cramer’s V > .30; Cohen, 1988, 1992).

**p* < .05. ***p* < .01. ****p* < .001.

**Supplemental Table 5.5**

***Wellbeing—Stress***

|  | Overall | Clinical | | | | | Non-Clinical |
| --- | --- | --- | --- | --- | --- | --- | --- |
|  |  | Overall | Professional Setting | | | |  |
|  |  |  | Institution Only | Institution/ Private Practice | Private Practice Only | Differences |  |
| Student loan debt [*M*(*SD*)] | 37 (36) | 39 (36) | 48 (36) | 38 (35) | 26 (35) | *F*(2,227) = 0.15,  η^2^ = 0.001 | 15 (27) |
| Clinical degree |  |  |  |  |  |  |  |
| *PhD* | - | 32 (35) | 40 (36) | 31 (34) | 24 (35) |  | - |
| *PsyD* | - | 51 (36) | 61 (36) | 45 (33) | 35 (35) |  | - |
| *Differences* |  | *t*(214) = 3.91***,  *d* = **0.55** |  |  |  |  |  |
| Retirement planning [*M*(*SD*)] | 39 (27) | 39 (28) | 40 (26) | 38 (26) | 40 (31) | *F*(2,272) = 0.59,  η^2^ = 0.004 | 35 (26) |

*Note.* “How would you quantify your stress related to the following areas for 2018?” Based on a sliding scale from 0 “Not at all stressed” to 100 “Extremely stressed” with additional “No Opinion” option. Sample sizes generally include all eligible participants in the overall (*n* = 351), clinical (n = 323), and non-clinical samples (*n* = 35). Professional Setting further includes clinical participants working in institutional settings only (n = 119), both institutional settings and private practice (n = 90), or private practice only (n = 107). Actual sample sizes reduced due to item non-response. Comparisons included participants’ years in the field as a covariate, based on the significant differences across professional settings for this variable (Supplemental Table 1.1). **Bold** indicates significant difference based on measure of association (i.e., at least a medium effect *d* > 0.50 or *η_p_^2^* > .06; Cohen, 1988, 1992).

**p* < .05. ***p* < .01. ****p* < .001.

**Supplemental Table 6.1**

***Satisfaction with Survey Instrument***

|  | Overall | Clinical | | | | | Non-Clinical |
| --- | --- | --- | --- | --- | --- | --- | --- |
|  |  | Overall | Professional Setting | | | |  |
|  |  |  | Institution Only | Institution/ Private Practice | Private Practice Only | Differences |  |
| Categorical (*%*)^a^ |  |  |  |  |  |  |  |
| *Completely Satisfied* | 26.6 | 25.4 | 24.3 | 28.4 | 28.7 | *χ*^2^ (10, N = 304) = 4.15, Cramer’s V = 0.08 | 25.0 |
| *Somewhat Satisfied* | 49.7 | 47.1 | 54.8 | 46.6 | 46.5 |  | 59.4 |
| *Neither Satisfied nor Dissatisfied* | 17.7 | 17.3 | 15.7 | 19.3 | 17.8 |  | 9.4 |
| *Somewhat Dissatisfied* | 3.3 | 3.1 | 2.6 | 3.4 | 4.0 |  | 3.1 |
| *Completely Dissatisfied* | 0.3 | 0.3 | 0.9 | 0 | 0 |  | 3.1 |
| *No Opinion* | 2.4 | 2.2 | 1.7 | 2.3 | 3.0 |  | 3.1 |

*Note.* “Constructing a survey for diverse professionals can be challenging. Which of the following best describes your view about the current survey’s ability to capture important information related to your training, practice, and income?” Sample sizes generally include all eligible participants in the overall (*n* = 351), clinical (n = 323), and non-clinical samples (*n* = 35). Professional Setting further includes clinical participants working in institutional settings only (n = 119), both institutional settings and private practice (n = 90), or private practice only (n = 107). Actual sample sizes reduced due to item non-response. **Bold** indicates significant difference based on measure of association (i.e., at least a medium effect per Cramer’s V > .30; Cohen, 1988, 1992).

**p* < .05. ***p* < .01. ****p* < .001.

**Supplemental Table 6.2**

***Feedback about Changes to Future Surveys***

| **Common Theme** | **Key Points** |
| --- | --- |
| Identification and Privacy Concerns | - Several respondents expressed concerns about potentially identifiable information, especially regarding birth month/year, gender, and zip code. - Suggestions were made to consider the potential risk of data breaches, especially with detailed demographic information. |
| Relevance to Diverse Practices | - Many respondents noted that the survey did not fully capture the diversity of forensic practices, including variations in employment settings, responsibilities, and income structures. - Some felt that certain questions and response options were more tailored to specific types of forensic work and may not be applicable to all practitioners. |
| Income and Financial Considerations | - Requests were made to include additional details on income sources, such as retirement income and specific financial stressors. - Some respondents suggested capturing the impact of student loans on various life milestones like marriage, children, and home purchases. |
| Work Satisfaction and Barriers | - Feedback was provided on the survey's assessment of work satisfaction, with suggestions to include more nuanced questions about barriers, challenges, and satisfaction factors. - Some respondents suggested exploring the impact of work setting changes on satisfaction and including qualitative feedback. |
| Specific Work Tasks and Responsibilities | - Requests were made for more detailed questions about specific work tasks, responsibilities, and the challenges faced in different forensic roles |
| Flexibility in Response Options | - Respondents suggested including more open-ended boxes, allowing for detailed responses and explanations, especially when certain questions did not fully capture their experiences |
| Time Frame and Retired Practitioners | - Some respondents found it challenging to answer questions based on a specific year, especially if their work situation changed during that period. - Suggestions were made to include options or qualifiers for retired practitioners and those working part-time or in unconventional roles. |
| Suggestions for Additional Variables | - Recommendations were made to include additional variables such as plans for public service loan forgiveness, income-driven payment plans, and the ability to alter income based on individual needs. - These themes provide insights into the perspectives of respondents and offer valuable input for refining the survey instrument to better capture the diversity of forensic practices and practitioners. |

*Note.* ﻿“What changes would you make to future income surveys to improve its ability to capture important information related to your training, practice, and income?” Sample size generally includes all eligible participants (*n* = 351). All language included in this table was generated by ChatGPT 3.5 (chat.openai.com) using the prompt, “I have a number of reviews of a survey instrument. I would like you to provide a summary of common themes and key points.” The initial output was reviewed by the first author (CL) for inclusion based on relevance to the current project; otherwise, no significant modifications were made to preserve the integrity of the analysis.

# Survey Instrument

Start of Block: Informed Consent

Q1.1 **CONSENT TO PARTICIPATE IN A RESEARCH STUDY**

Title of Research Study: Forensic Psychology Income Survey

Principal Investigator:    Casey LaDuke, PhD
The City University of New York
John Jay College of Criminal Justice
Department of Psychology

Co-Investigators:   Eve Brank, JD, PhD (University of Nebraska-Lincoln)
David DeMatteo, JD, PhD, ABPP (Forensic) (Drexel University)
Antoinette Kavanaugh, PhD, ABPP (Forensic) (private practice)

Eligibility: Please complete this research study only if you are 18 years of age or older, and were involved in forensic psychology during the 2018 calendar year (i.e., 1/1/18-12/31/18). Based on the *Specialty Guidelines for Forensic Psychology* (2013), you are included in this field if you: (a) study, research, teach, and/or practice in any sub-discipline of psychology (e.g., clinical, social, developmental, cognitive); and (b) apply your scientific, technical, or specialized knowledge to any aspect of the law (e.g., legal, contractual, and/or administrative matters). You are being asked to participate in this research study because you are a member of a professional organization popular among students and/or professionals in forensic psychology.

Purpose: The purpose of this research study is to strengthen the field of forensic psychology. Forensic psychology is an established and rapidly expanding area of psychology, the development of which has been partly aided by surveys about professional practice and training in the field. One significant gap in our current knowledge of the field concerns the financial aspects of forensic psychology, such as student loan debt accrued during professional training, income resulting from professional practice, and how these financial factors influence job satisfaction, life satisfaction, and financial planning. The current research seeks to replicate the success of so-called “salary surveys” in allied fields by gathering information about the past training, current practice, and financial considerations of students and professionals in forensic psychology. Ultimately, the goal of this research is to provide valuable information for prospective trainees looking to enter the field, current students and professionals looking to develop and maintain successful careers in the field, and field leaders looking to further develop forensic psychology as a whole.

Procedures:  If you choose to participate in this research study, you will complete a brief and anonymous online survey that asks basic questions about your training, professional practice, financial considerations, subjective well-being, and demographics.

Time Commitment: Participants can only complete the online survey once. Participation in this research study is expected to last around 10 minutes.

Potential Risks or Discomforts: Your participation in this online survey involves risks similar to a person’s everyday use of the Internet. Additionally, as with any survey research, it is possible that you may experience emotional discomfort associated with the content questions of the online survey.

Potential Benefits: You will not directly benefit from your participation in this research study. The benefit of the research study is an increased understanding of forensic psychology, which will be available to all participants and non-participants following data collection.

Payment for Participation: You will not receive any payment for participating in this research study. Rather, you will be provided the opportunity to help decide how to donate a total of $1000 among several worthy causes in forensic psychology. Specifically, each participant will be asked to indicate how much they would donate to several pre-selected organizations in forensic psychology if they had $1000 to do so. At the end of data collection, a total of $1000 (funded by the Principal Investigator) will be donated to those organizations based directly on the averaged responses of all participants. The final amounts donated will be reported in all publications and presentations related to this research.

Q1.2

New Information: You will be notified about any new information regarding this study that may affect your willingness to participate in a timely manner.

Confidentiality: We will make our best efforts to maintain the confidentiality of any information that is collected during this research study. We will disclose this information only with your permission or as required by law. Otherwise, we will protect your confidentiality in a number of ways. We have purposefully avoided collecting identifying data about you during the recruitment phase by using electronic mailing lists and listservs managed solely by third-party entities. No identifying information will be requested about you at any time, and your responses to all survey items will be anonymous. You will submit your survey responses electronically using a reputable and secure online survey tool (i.e., Qualtrics). Qualtrics will collect your IP address, which will be accessible only to the PI and only to screen for multiple submissions; once the initial data screening is complete, the PI will delete all IP address information from the dataset before making it available to the co-investigators and open-access (see below). All data will be stored without identifiers or codes in secure electronic files that are initially only accessible by the PI and then the co-investigators. Additionally, data will be stored for future use, including planned follow-up studies by the investigators and open-access for other researchers in strict adherence to the relevant policies and procedures of the selected open-access resource (e.g., data repositories, publishing journal, or independent webpage). All results will be reported in aggregate; no individual responses will be reported in any publication or report that emerges from this study.

The research team, authorized CUNY staff, and government agencies that oversee this type of research may have access to research data and records to monitor the research. Research records provided to authorized, non-CUNY individuals will not contain identifiable information about you. Publications or presentations that result from this study will not identify you by name.

Participants’ Rights: Your participation in this research study is entirely voluntary. If you decide not to participate, there will be no penalty to you, and you will not lose any benefits to which you are otherwise entitled. You can decide to withdraw your consent and stop participating in the research at any time, without any penalty.

Questions, Comments or Concerns: If you have questions, comments, or concerns about the research, please contact to the Principal Investigator by email (claduke@jjay.cuny.edu) or mail (John Jay College of Criminal Justice Department of Psychology, 524 W. 59th Street, New York, NY 10019). If you have questions about your rights as a research participant, or you have comments or concerns that you would like to discuss with someone other than the researchers, call the CUNY Research Compliance Administrator at 646-664-8918 or email HRPP@cuny.edu.  Alternatively, you may write to: CUNY Office of the Vice Chancellor for Research, Attn: Research Compliance Administrator, 205 East 42nd Street, New York, NY 10017.

Endorsements: This research is endorsed by the American Academy of Forensic Psychology (AAFP).

Q1.3 Consent: If you agree to participate in this research study, please click “I agree” below to indicate your consent and be taken to the online survey. You are encouraged to save a copy of this consent form for your records.

- I agree (1)
- I do not agree (2)

End of Block: Informed Consent

Start of Block: Inclusion/Exclusion

Q2.1 Are you currently 18 years of age or older?

- Yes (1)
- No (2)

Skip To: End of Block If Are you currently 18 years of age or older? = No

Display This Question:

If Are you currently 18 years of age or older? = Yes

Q2.2 For the following questions, forensic psychology refers to “professional practice by any psychologist working within any subdiscipline of psychology (e.g., clinical, developmental, social, cognitive) when applying the scientific, technical, or specialized knowledge of psychology to the law to assist in addressing legal, contractual, and administrative matters” (APA, 2013).   Have you ever completed training, research, teaching, and/or practice in forensic psychology?

- Yes (1)
- No (2)

Skip To: End of Block If For the following questions, forensic psychology refers to “professional practice by any psycholo... = No

Display This Question:

If For the following questions, forensic psychology refers to “professional practice by any psycholo... = Yes

Q2.3 Were you involved in any training, research, teaching, and/or practice in forensic psychology during the 2018 calendar year (i.e., between 1/1/18 and 12/31/18)?

- Yes (1)
- No (2)

End of Block: Inclusion/Exclusion

Start of Block: Donation (initial)

Q3.1 If you had $1000 USD to donate, how much would you donate to the following causes?   *Your responses must equal $1000 USD and will be confirmed at the end of the survey.*

- American Psychology-Law Society (AP-LS) Student Travel Awards : _______ (1)
- American Society of Trial Consultants (ASTC) : _______ (2)
- Community for Psychologists in Independent Practice (Div42) Fund : _______ (3)
- National Register of Health Service Psychologists (National Register) Awards : _______ (4)

Total : ________

End of Block: Donation (initial)

Start of Block: Piping

Q4.1 Which of the following best describes your primary area(s) of professional training, research, teaching, and/or practice in forensic psychology? (select all that apply)

- Clinical (e.g., assessment, intervention, clinical research) (1)
- Non-clinical (e.g., experimental, social, legal-psychology, program evaluation) (2)
- Legal (e.g., legal practice or scholarship) (3)
- I am currently completing graduate/professional training (clinical, non-clinical, or legal) (4)

End of Block: Piping

Start of Block: Trainee items

Q5.1
Some of the following questions are about finances. What currency will you be using in your responses?
*Please respond to all relevant survey items based on your selected currency.*
United States Dollar (USD) (1)

- Canadian Dollar (CAD) (2)
- Euro (EUR) (3)
- Pound sterling (GBR) (4)
- Mexican Peso (MXN) (5)
- Other (6) __________________________________________________

Q5.2 Which of the following graduate/professional degrees are you currently completing? (select all that apply)

- Clinical doctoral (e.g., clinical psychology, forensic psychology) (1)
- Non-clinical doctoral (e.g., psychology & law, social psychology, developmental psychology) (2)
- Terminal Master’s (e.g., MS or MA) (3)
- Legal (e.g., JD, MLS, LLB) (4)
- Other (5) __________________________________________________

Display This Question:

If Which of the following graduate/professional degrees are you currently completing? (select all th... = Clinical doctoral (e.g., clinical psychology, forensic psychology)

Q5.3 In which type of clinical doctoral degree are you currently enrolled?

- PhD (1)
- PsyD (2)
- EdD (3)
- Other (4) __________________________________________________

Display This Question:

If Which of the following graduate/professional degrees are you currently completing? (select all th... = Clinical doctoral (e.g., clinical psychology, forensic psychology)

Q5.4 In which type of clinical doctoral program are you currently enrolled?

- Clinical Psychology (1)
- Forensic Psychology (2)
- Clinical Forensic Psychology (3)
- Counseling Psychology (4)
- Other (5) __________________________________________________

Display This Question:

If Which of the following graduate/professional degrees are you currently completing? (select all th... = Clinical doctoral (e.g., clinical psychology, forensic psychology)

Q5.5 To the best of your knowledge, is your program APA/CPA accredited?

- Yes (1)
- No (2)
- I don’t know (3)
- Not applicable (4)

Display This Question:

If Which of the following graduate/professional degrees are you currently completing? (select all th... = Non-clinical doctoral (e.g., psychology & law, social psychology, developmental psychology)

Q5.6 In which type of non-clinical doctoral degree are you currently enrolled?

- PhD (1)
- EdD (2)
- Other (3) __________________________________________________

Display This Question:

If Which of the following graduate/professional degrees are you currently completing? (select all th... = Non-clinical doctoral (e.g., psychology & law, social psychology, developmental psychology)

Q5.7 In which type of non-clinical doctoral program are you currently enrolled?

- Psychology & Law (1)
- Social Psychology (2)
- Developmental Psychology (3)
- Psychology & Social Behavior (4)
- Legal Psychology (5)
- Experimental Psychology (6)
- Cognitive Psychology (7)
- Neuroscience (8)
- Criminal Justice (9)
- Other (10) __________________________________________________

Display This Question:

If Which of the following graduate/professional degrees are you currently completing? (select all th... = Terminal Master’s (e.g., MS or MA)

Q5.8 In which type of terminal Master’s degree are you currently enrolled?

- MA (1)
- MS (2)
- Other (3) __________________________________________________

Display This Question:

If Which of the following graduate/professional degrees are you currently completing? (select all th... = Terminal Master’s (e.g., MS or MA)

Q5.9 In which type of terminal Master’s program are you currently enrolled?

- Psychology (1)
- Forensic psychology (2)
- Legal psychology (3)
- Forensic & Legal Psychology (4)
- Forensic Mental Health (5)
- Forensic Mental Health Counseling (6)
- Criminal justice (7)
- Other (8) __________________________________________________

Display This Question:

If Which of the following graduate/professional degrees are you currently completing? (select all th... = Legal (e.g., JD, MLS, LLB)

Q5.10 In which type of legal degree program are you currently enrolled?

- JD (1)
- MLS (2)
- LLB (3)
- Other (4) __________________________________________________

Display This Question:

If Which of the following graduate/professional degrees are you currently completing? (select all th... = Legal (e.g., JD, MLS, LLB)

Q5.11 To the best of your knowledge, is your program ABA accredited?

- Yes (1)
- No (2)
- I don’t know (3)
- Not applicable (4)

Q5.12 How are you funding your graduate/professional degree training? (select all that apply)

*If "stipend" is selected, please enter the approximate amount.*

- Stipend from graduate program (i.e., not internship or postdoc) (1) _____________________________________________
- Tuition remission (full) (2)
- Tuition remission (partial) (3)
- Teaching Assistantship (4)
- Research Assistantship (5)
- Administrative position (6)
- Student loan (7)
- Personal loan (8)
- External employment (9)
- Other (10) __________________________________________________

Display This Question:

If Which of the following graduate/professional degrees are you currently completing? (select all th... = Clinical doctoral (e.g., clinical psychology, forensic psychology)

Q5.13 Are you currently completing a predoctoral psychology internship?

- Yes (1)
- No (2)

Display This Question:

If Are you currently completing a predoctoral psychology internship? = Yes

Q5.14 What is the current annual stipend for your internship? (please enter in whole dollar amounts)

________________________________________________________________

Display This Question:

If Which of the following graduate/professional degrees are you currently completing? (select all th... = Clinical doctoral (e.g., clinical psychology, forensic psychology)

And Are you currently completing a predoctoral psychology internship? = No

Or Which of the following graduate/professional degrees are you currently completing? (select all th... = Non-clinical doctoral (e.g., psychology & law, social psychology, developmental psychology)

Q5.15 Are you currently completing a postdoctoral fellowship?

- Yes (1)
- No (2)

Display This Question:

If Are you currently completing a postdoctoral fellowship? = Yes

Q5.16 What is your current annual stipend for your fellowship? (please enter in whole dollar amounts)

________________________________________________________________

Q5.17 Approximately how much student loan debt do you believe you will owe at the end of your graduate/professional training? (please enter in whole dollar amounts)

________________________________________________________________

Q5.18 At what age do you expect to retire?

Age (1)

▼ 18 (1) ... 135 (118)

Q5.19 How would you rank the following factors in terms of their importance to determining the timing of your retirement?

- ______ Financial status (1)
- ______ Personal health (2)
- ______ Quality of work life (3)
- ______ Family considerations (4)
- ______ Continued availability of Social Security or some other governmental benefit (5)

End of Block: Trainee items

Start of Block: Professional Finances

Q6.1 The first set of questions are about finances, including income and benefits.

Q6.2
What currency will you be using in your responses?
 
*Please respond to all relevant survey items based on your selected currency.*

- United States Dollar (USD) (1)
- Canadian Dollar (CAD) (2)
- Euro (EUR) (3)
- Pound sterling (GBR) (4)
- Mexican Peso (MXN) (5)
- Other (6) __________________________________________________

Q6.3 In completing the following items please keep in mind:

- Income includes total pre-tax income plus deferred money set aside in the same year
- Only include income from 2018 (i.e., 01/01/2018-12/31/2018)
- Only include your income (i.e., not that of a spouse or any other joint income)
- If you work part-time, do not extrapolate to a full-time equivalent
- Enter only whole amounts with no commas or decimals (e.g., 50, 500, 5000, 50000)
- If you do not know the specific amounts offhand, please provide your best estimation

Q6.4 Approximately how much was your overall income in 2018?

________________________________________________________________

Q6.5 Approximately how much of your 2018 income was related to forensic psychology?

 *Defined as any income related to studying, researching, teaching, and/or practicing in any sub-discipline of psychology (e.g., clinical, social, developmental, cognitive) in which you apply your scientific, technical, or specialized knowledge to any aspect of the law (e.g., legal, contractual, and/or administrative matters).*

 Please respond in "whole dollar amount" or "percent of total income" but not both.

- Whole dollar amount (1) __________________________________________________
- Percent of total income (2) __________________________________________________

Q6.6 Approximately how much of your 2018 income was related to other psychology (not forensic)?   *Defined as any income related to studying, researching, teaching, and/or practicing in any sub-discipline of psychology (e.g., clinical, social, developmental, cognitive) that does NOT include applying your scientific, technical, or specialized knowledge to any aspect of the law.*   Please respond in "whole dollar amount" or "percent of total income" but not both.

- Whole dollar amount (1) __________________________________________________
- Percent of total income (2) __________________________________________________

Q6.7 Approximately how much of your 2018 income was related to other professional activities (not psychology)?

*Defined as any income related to professional activities that does NOT include studying, researching, teaching, and/or practicing in any sub-discipline of psychology.*

Please respond in "whole dollar amount" or "percent of total income" but not both.

- Whole dollar amount (1) __________________________________________________
- Percent of total income (2) __________________________________________________

Q6.8 Which of the following benefits were available to you as part of your forensic psychology work activities in 2018? (select all that apply)

- Health insurance (1)
- Life insurance (2)
- Dental insurance (3)
- Vision insurance (4)
- Retirement plan (5)
- Short-term disability insurance (6)
- Long-term disability insurance (7)
- Flexible Spending Accounts (FSAs) (8)
- Health Savings Accounts (HSAs) (9)
- Paid maternity leave (10)
- Paid paternity leave (11)
- Other paid personal medical leave (e.g., to recover from a procedure) (12)
- Other paid family medical leave (e.g., to care for an ailing family member) (13)
- Childcare benefits (14)
- Flexible schedule (15)
- Telecommuting (16)
- Other (17) __________________________________________________

Q6.9 Approximately how many days per week (on average) did you complete forensic psychology work activities in the following locations in 2018? (should not exceed 7 days total)

- In the office : _______ (1)
- Remotely (i.e., telecommuting) : _______ (2)
- Other : _______ (3)

Total : ________

Q6.10 At what age do you expect to retire?

Age (1)

▼ 18 (1) ... 135 (118)

Q6.11 How would you rank the following factors in terms of their importance to determining the timing of your retirement?

- ______ Financial status (1)
- ______ Personal health (2)
- ______ Quality of work life (3)
- ______ Family considerations (4)
- ______ Continued availability of Social Security or some other governmental benefit (5)

End of Block: Professional Finances

Start of Block: Professional Training

Q7.1 The following sets of questions are about factors that may relate to finances, including professional training and practice.

Display This Question:

If Which of the following best describes your primary area(s) of professional training, research, te... = Clinical (e.g., assessment, intervention, clinical research)

Q7.2 Which type of clinical degree did you complete? (please select your highest degree)

- PhD (1)
- PsyD (2)
- EdD (3)
- MA (terminal) (4)
- MS (terminal) (5)
- Other (6) __________________________________________________

Display This Question:

If Which of the following best describes your primary area(s) of professional training, research, te... = Clinical (e.g., assessment, intervention, clinical research)

Q7.3 Which type of clinical program did you complete? (please select that of your highest degree)

- Clinical Psychology (1)
- Forensic Psychology (2)
- Clinical Forensic Psychology (3)
- Counseling Psychology (4)
- Forensic Mental Health Counseling (5)
- Other (6) __________________________________________________

Display This Question:

If Which of the following best describes your primary area(s) of professional training, research, te... = Clinical (e.g., assessment, intervention, clinical research)

Q7.4 In what year did you complete your highest clinical degree?

Year (1)

▼ 2018 (1) ... 1901 (118)

Display This Question:

If Which of the following best describes your primary area(s) of professional training, research, te... = Clinical (e.g., assessment, intervention, clinical research)

Q7.5 To the best of your knowledge, was your clinical program APA/CPA accredited at your time of completion?

- Yes (1)
- No (2)
- I don’t know (3)
- Not applicable (4)

Display This Question:

If Which of the following best describes your primary area(s) of professional training, research, te... = Clinical (e.g., assessment, intervention, clinical research)

Q7.6 Did you complete a predoctoral psychology internship?

- Yes (1)
- No (2)

Display This Question:

If Did you complete a predoctoral psychology internship? = Yes

Q7.7 To the best of your knowledge, was your internship APA/CPA accredited?

- Yes (1)
- No (2)
- I don't know (3)
- Not applicable (4)

Display This Question:

If Which of the following best describes your primary area(s) of professional training, research, te... = Non-clinical (e.g., experimental, social, legal-psychology, program evaluation)

Q7.8 Which type of non-clinical degree did you complete? (please select your highest degree)

- PhD (1)
- EdD (2)
- MA (terminal) (3)
- MS (terminal) (4)
- Other (5) __________________________________________________

Display This Question:

If Which of the following best describes your primary area(s) of professional training, research, te... = Non-clinical (e.g., experimental, social, legal-psychology, program evaluation)

Q7.9 Which type of non-clinical program did you complete? (please select that of your highest degree)

- Psychology & Law (1)
- Social Psychology (2)
- Developmental Psychology (3)
- Psychology & Social Behavior (4)
- Legal Psychology (5)
- Experimental Psychology (6)
- Criminal justice (7)
- Other (8) __________________________________________________

Display This Question:

If Which of the following best describes your primary area(s) of professional training, research, te... = Non-clinical (e.g., experimental, social, legal-psychology, program evaluation)

Q7.10 In what year did you complete your highest non-clinical degree?

Year (1)

▼ 2018 (1) ... 1901 (118)

Display This Question:

If Which of the following best describes your primary area(s) of professional training, research, te... = Clinical (e.g., assessment, intervention, clinical research)

Or Which of the following best describes your primary area(s) of professional training, research, te... = Non-clinical (e.g., experimental, social, legal-psychology, program evaluation)

Q7.11 Did you complete a postdoctoral fellowship?

- Yes (1)
- No (2)

Display This Question:

If Which of the following best describes your primary area(s) of professional training, research, te... = Legal (e.g., legal practice or scholarship)

Q7.12 Which type of legal degree did you complete? (please select your highest degree)

- JD (1)
- MLS (2)
- LLB (3)
- Other (4) __________________________________________________

Display This Question:

If Which of the following best describes your primary area(s) of professional training, research, te... = Legal (e.g., legal practice or scholarship)

Q7.13 In what year did you complete your highest legal degree?

Year (1)

▼ 2018 (1) ... 1901 (118)

Display This Question:

If Which of the following best describes your primary area(s) of professional training, research, te... = Legal (e.g., legal practice or scholarship)

Q7.14 To the best of your knowledge, was your legal degree program ABA accredited at your time of completion?

- Yes (1)
- No (2)
- I don’t know (3)
- Not applicable (4)

Q7.15 How did you fund your graduate/professional degree training? (select all that apply)   *If any "stipend" option is selected, please enter the approximate amount in whole dollar amounts.*

- Graduate program stipend (1) __________________________________________________

Display This Choice:

If Which of the following graduate/professional degrees are you currently completing? (select all th... = Clinical doctoral (e.g., clinical psychology, forensic psychology)

- Psychology internship stipend (2) __________________________________________________

Display This Choice:

If Which of the following best describes your primary area(s) of professional training, research, te... = Clinical (e.g., assessment, intervention, clinical research)

Or Which of the following best describes your primary area(s) of professional training, research, te... = Non-clinical (e.g., experimental, social, legal-psychology, program evaluation)

- Psychology postdoctoral fellowship stipend (3) __________________________________________________
- Tuition remission (full) (4)
- Tuition remission (partial) (5)
- Teaching Assistantship (6)
- Research Assistantship (7)
- Administrative position (8)
- Student loan (9)
- Personal loan (10)
- External employment (11)
- Other (12) __________________________________________________

Q7.16 Approximately how much student loan debt did you owe at the end of your graduate/professional training?

________________________________________________________________

Q7.17 Approximately how much student loan debt do you owe now?

________________________________________________________________

End of Block: Professional Training

Start of Block: Professional Practice

Display This Question:

If Which of the following best describes your primary area(s) of professional training, research, te... = Clinical (e.g., assessment, intervention, clinical research)

Q8.1 Were you licensed to practice psychology in 2018?

- Yes (1)
- No (2)
- I prefer not to respond to this item (3)

Display This Question:

If Were you licensed to practice psychology in 2018? = Yes

Q8.2 In what year did you first obtain licensure?
 
*Leave blank if you would prefer not to respond to this item*

Year (1)

▼ 2018 (1) ... 1901 (118)

Display This Question:

If Which of the following best describes your primary area(s) of professional training, research, te... = Clinical (e.g., assessment, intervention, clinical research)

Q8.3 Were you board certified in 2018?

- Yes (1)
- No (2)
- I prefer not to respond to this item (3)

Display This Question:

If Were you board certified in 2018? = Yes

Q8.4
Please indicate through which board(s) you held certification in 2018? (select all that apply)

*Please also enter the year you first obtained each selected board certification (YYYY)*

*Leave blank if you would prefer not to respond to this item*

- American Board of Forensic Psychology (1) __________________________________________________
- American Board of Clinical Psychology (2) __________________________________________________
- American Board of Clinical Child & Adolescent Psychology (3) ________________________________________________
- American Board of Clinical Health Psychology (4) __________________________________________________
- American Board of Clinical Neuropsychology (5) __________________________________________________
- American Board of Cognitive & Behavioral Psychology (6) __________________________________________________
- American Board of Counseling Psychology (7) __________________________________________________
- American Board of Couple & Family Psychology (8) __________________________________________________
- American Board of Geropsychology (9) __________________________________________________
- American Board of Group Psychology (10) __________________________________________________
- American Board of Organizational & Business Consulting Psychology (11) ______________________________________
- American Board of Police & Public Safety Psychology (12) __________________________________________________
- American Board of Psychoanalysis in Psychology (13) __________________________________________________
- American Board of Rehabilitation Psychology (14) __________________________________________________
- American Board of School Psychology (15) __________________________________________________
- American College of Forensic Psychology (16) __________________________________________________
- American College of Forensic Examiners (17) __________________________________________________
- Other (18) __________________________________________________

Q8.5 How would you describe your overall psychology-related work status in 2018?    *Please select the one that describes your work status for the majority of 2018.*

- Full time (35 hours per week or more) (1)
- Part time (less than 35 hours per week) (2)
- Full time (primary) plus part time (secondary) (3)
- Retired (4)
- Disabled, not working (5)
- Unemployed, seeking employment (6)

Q8.6 How would you characterize your overall psychology-related work status in 2018?

- About the same as 2017 (1)
- Significantly less than 2017 (2)
- Significant more than 2017 (3)

Q8.7 In which setting did you receive income for forensic psychology work activities in 2018?

- Institution only (1)
- Private practice only (2)
- Both institution and private practice (3)
- I prefer not to respond to this item (4)

Display This Question:

If In which setting did you receive income for forensic psychology work activities in 2018? = Both institution and private practice

Q8.8 Approximately what percent of your forensic psychology professional time was spent in institution(s) and private practice in 2018?

 *Responses should total 100%*

- Institution(s) : _______ (1)
- Private practice : _______ (2)

Total : ________

Display This Question:

If In which setting did you receive income for forensic psychology work activities in 2018? = Institution only

Or In which setting did you receive income for forensic psychology work activities in 2018? = Both institution and private practice

Q8.9 What time period related to your forensic psychology institutional income in 2018?

- 9-10 months (1)
- 11-12 months (2)
- Other (3) __________________________________________________

Display This Question:

If In which setting did you receive income for forensic psychology work activities in 2018? = Institution only

Or In which setting did you receive income for forensic psychology work activities in 2018? = Both institution and private practice

Q8.10 Professionals in forensic psychology work in a variety of institutions.    Which of the following best describes the primary institution(s) from which you received income for forensic psychology work activities in 2018? (select all that apply)   *Leave blank if you would prefer not to respond to this item*

- Federal prison (1)
- State prison (2)
- Local jail (3)
- Forensic juvenile facility (4)
- Probation/parole department (5)
- Other correctional facility (6)
- Public Defender’s office (7)
- District Attorney’s office (8)
- Court clinic (9)
- Worker’s compensation clinic (10)
- Mediation (11)
- Primary University Hospital or Academic Medical Center (12)
- Academic Affiliated Hospital or Medical Center (non-VA) (13)
- General Hospital (Public) (14)
- General Hospital (Private) (15)
- Psychiatric Hospital (Public) (16)
- Psychiatric Hospital (Private) (17)
- Other Specialty Hospital (Public) (18)
- Other Specialty Hospital (Private) (19)
- VA Hospital or VA Medical Center (with academic affiliation) (20)
- VA Hospital or VA Medical Center (no academic affiliation) (21)
- Military hospital (22)
- General Clinic (community) (23)
- Psychiatric Clinic (community) (24)
- Specialty Clinic (community) (25)
- General Clinic (department/school affiliated) (26)
- Psychiatric Clinic (department/school affiliated) (27)
- Other Specialty Clinic (department/school affiliated) (28)
- Four-year university/college (non-medical, with doctoral psychology program) (30)
- Four-year university/college (non-medical, without doctoral psychology program) (29)
- Doctoral psychology program in medical/educational setting (no undergraduate program) (31)
- Professional School of Psychology (32)
- Community College (33)
- Schools (i.e., any institution of primary or secondary education) (34)
- Partial hospitalization/intensive outpatient program (35)
- Residential/group home (36)
- Research foundation (non-hospital) (37)
- Military service (outside of military hospital) (38)
- Other (39) __________________________________________________

Display This Question:

If In which setting did you receive income for forensic psychology work activities in 2018? = Institution only

Or In which setting did you receive income for forensic psychology work activities in 2018? = Both institution and private practice

Carry Forward Selected Choices from "Professionals in forensic psychology work in a variety of institutions.    Which of the following best describes the primary institution(s) from which you received income for forensic psychology work activities in 2018? (select all that apply)   Leave blank if you would prefer not to respond to this item"

Q8.11 Approximately how many hours per week (on average) did you spend in the following institutions completing forensic psychology work activities in 2018?    *Should include all work time, including weekends* *Responses should be considered mutually exclusive* *Leave blank if you would prefer not to respond to this item*

- Federal prison (1) __________________________________________________
- State prison (2) __________________________________________________
- Local jail (3) __________________________________________________
- Forensic juvenile facility (4) __________________________________________________
- Probation/parole department (5) __________________________________________________
- Other correctional facility (6) __________________________________________________
- Public Defender’s office (7) __________________________________________________
- District Attorney’s office (8) __________________________________________________
- Court clinic (9) __________________________________________________
- Worker’s compensation clinic (10) __________________________________________________
- Mediation (11) __________________________________________________
- Primary University Hospital or Academic Medical Center (12) __________________________________________________
- Academic Affiliated Hospital or Medical Center (non-VA) (13) __________________________________________________
- General Hospital (Public) (14) __________________________________________________
- General Hospital (Private) (15) __________________________________________________
- Psychiatric Hospital (Public) (16) __________________________________________________
- Psychiatric Hospital (Private) (17) __________________________________________________
- Other Specialty Hospital (Public) (18) __________________________________________________
- Other Specialty Hospital (Private) (19) __________________________________________________
- VA Hospital or VA Medical Center (with academic affiliation) (20) __________________________________________________
- VA Hospital or VA Medical Center (no academic affiliation) (21) __________________________________________________
- Military hospital (22) __________________________________________________
- General Clinic (community) (23) __________________________________________________
- Psychiatric Clinic (community) (24) __________________________________________________
- Specialty Clinic (community) (25) __________________________________________________
- General Clinic (department/school affiliated) (26) __________________________________________________
- Psychiatric Clinic (department/school affiliated) (27) __________________________________________________
- Other Specialty Clinic (department/school affiliated) (28) __________________________________________________
- Four-year university/college (non-medical, with doctoral psychology program) (29) _______________________________________
- Four-year university/college (non-medical, without doctoral psychology program) (30) ____________________________________
- Doctoral psychology program in medical/educational setting (no undergraduate program) (31) ______________________________
- Professional School of Psychology (32) __________________________________________________
- Community College (33) __________________________________________________
- Schools (i.e., any institution of primary or secondary education) (34) __________________________________________________
- Partial hospitalization/intensive outpatient program (35) __________________________________________________
- Residential/group home (36) __________________________________________________
- Research foundation (non-hospital) (37) __________________________________________________
- Military service (outside of military hospital) (38) __________________________________________________
- Other (39) __________________________________________________

Display This Question:

If In which setting did you receive income for forensic psychology work activities in 2018? = Institution only

Or In which setting did you receive income for forensic psychology work activities in 2018? = Both institution and private practice

Q8.12 Which of the following best describes the primary institutional department(s) from which you received income for forensic psychology work activities in 2018? (select all that apply)

- Psychology (1)
- Psychiatry (2)
- Social Science (3)
- Public Health (4)
- Criminal Justice (5)
- Criminology (6)
- Other (7) __________________________________________________
- I prefer not to respond to this item (8)

Display This Question:

If In which setting did you receive income for forensic psychology work activities in 2018? = Institution only

Or In which setting did you receive income for forensic psychology work activities in 2018? = Both institution and private practice

Q8.13 Which of the following best describes the primary institutional title(s) from which you received income for forensic psychology work activities in 2018? (select all that apply)

- Staff Psychologist (1)
- Lecturer/Instructor (2)
- Assistant Professor (3)
- Associate Professor (4)
- Professor (5)
- Distinguished Professor (6)
- Emeritus (7)
- Clinical Program Director (8)
- Research Program Director (9)
- Director of Clinical Training (10)
- Division Head (11)
- Vice or Associate of Assistant Chair (12)
- Department Chair (13)
- Provost (14)
- Dean (15)
- President (16)
- Chancellor (17)
- Other (18) __________________________________________________
- I prefer not to respond to this item (19)

Display This Question:

If In which setting did you receive income for forensic psychology work activities in 2018? = Private practice only

Or In which setting did you receive income for forensic psychology work activities in 2018? = Both institution and private practice

Q8.14 What was your role in private practice in 2018? (select all that apply)

- Partner (1)
- Employee (i.e., received a W-2 IRS tax form at year end) (2)
- Outside contractor (i.e., received a 1099 IRS tax form at year end) (3)
- Other (4) __________________________________________________
- I prefer not to respond to this item (5)

Q8.15 For what type of forensic psychology work activities did you receive income in 2018? (select all that apply)   *Leave blank if you would prefer not to respond to this item*

- Assessment of court functioning and administrative processes (1)
- Case consultation (2)
- Clinical practice–General assessment/evaluation (non-forensic) (3)
- Clinical practice–General intervention (non-forensic) (4)
- Clinical practice–Forensic assessment/evaluation (5)
- Clinical practice–Forensic intervention (6)
- Expert witness (7)
- Fact witness (8)
- Government (9)
- Health and mental health policy (10)
- Industry (11)
- Institutional service (e.g., committees and administrative duties) (12)
- Judiciary (13)
- Jury consultation (14)
- Legal practice (15)
- Mediation and dispute resolution (16)
- Research (clinical) (17)
- Research (non-clinical) (18)
- Supervision (clinical trainees) (19)
- Supervision (clinical and support personnel) (20)
- Teaching (21)
- Training of law enforcement personnel, lawyers, judges (22)
- Working within legislative committee staffs (23)
- Other (24) __________________________________________________

Carry Forward Selected Choices from "For what type of forensic psychology work activities did you receive income in 2018? (select all that apply)   Leave blank if you would prefer not to respond to this item"

Q8.16 Approximately how many hours per week (on average) did you spend on each of the following forensic psychology work activities in 2018?   *Should include all work time, including weekends* *Responses should be considered mutually exclusive* *Leave blank if you would prefer not to respond to this item*

- Assessment of court functioning and administrative processes (1) __________________________________________________
- Case consultation (2) __________________________________________________
- Clinical practice–General assessment/evaluation (non-forensic) (3) __________________________________________________
- Clinical practice–General intervention (non-forensic) (4) __________________________________________________
- Clinical practice–Forensic assessment/evaluation (5) __________________________________________________
- Clinical practice–Forensic intervention (6) __________________________________________________
- Expert witness (7) __________________________________________________
- Fact witness (8) __________________________________________________
- Government (9) __________________________________________________
- Health and mental health policy (10) __________________________________________________
- Industry (11) __________________________________________________
- Institutional service (e.g., committees and administrative duties) (12) __________________________________________________
- Judiciary (13) __________________________________________________
- Jury consultation (14) __________________________________________________
- Legal practice (15) __________________________________________________
- Mediation and dispute resolution (16) __________________________________________________
- Research (clinical) (17) __________________________________________________
- Research (non-clinical) (18) __________________________________________________
- Supervision (clinical trainees) (19) __________________________________________________
- Supervision (clinical and support personnel) (20) __________________________________________________
- Teaching (21) __________________________________________________
- Training of law enforcement personnel, lawyers, judges (22) __________________________________________________
- Working within legislative committee staffs (23) __________________________________________________
- Other (24) __________________________________________________

Q8.17 Additionally, approximately how many hours per week (on average) did you spend on professional volunteering (e.g., non-service related committees, elected positions, peer review) related to forensic psychology in 2018?

________________________________________________________________

Display This Question:

If For what type of forensic psychology work activities did you receive income in 2018? (select all... = Clinical practice–General assessment/evaluation (non-forensic)

Q8.18 With what frequency did you use the following types of tests in your general assessments/evaluations (non-forensic) in 2018?

|  | Always (1) | Most of the time (2) | About half the time (3) | Sometimes (4) | Never (5) |
| --- | --- | --- | --- | --- | --- |
| Intelligence tests (1) |  |  |  |  |  |
| Neuropsychological/cognitive tests (2) |  |  |  |  |  |
| Objective personality tests (3) |  |  |  |  |  |
| Projective personality tests (4) |  |  |  |  |  |

Display This Question:

If For what type of forensic psychology work activities did you receive income in 2018? (select all... = Clinical practice–Forensic assessment/evaluation

Q8.19 With what frequency did you use the following types of tests in your forensic assessments/evaluations in 2018?
 
*Forensically relevant instruments (FRIs) are those that measure clinical constructs related to legal issues (e.g., response style and psychopathy).*
 
*Forensic assessment instruments (FAIs) are those that directly measure legal constructs (e.g., functional abilities related to adjudicative competence)*

|  | Always (1) | Most of the time (2) | About half the time (3) | Sometimes (4) | Never (5) |
| --- | --- | --- | --- | --- | --- |
| Intelligence tests (1) |  |  |  |  |  |
| Neuropsychological/cognitive tests (2) |  |  |  |  |  |
| Objective personality tests (3) |  |  |  |  |  |
| Projective personality tests (4) |  |  |  |  |  |
| Forensically relevant instruments (FRIs) (5) |  |  |  |  |  |
| Forensic assessment instruments (FAIs) (6) |  |  |  |  |  |

Q8.20 Do you speak any language(s) other than English as part of your forensic psychology work activities?

- Yes (1)
- No (2)

Display This Question:

If Do you speak any language(s) other than English as part of your forensic psychology work activities? = Yes

Q8.21 What language(s) other than English do you speak as part of your forensic psychology work activities? (select all that apply)

- Spanish (1)
- Mandarin (2)
- Cantonese (3)
- Tagalog (4)
- Vietnamese (5)
- Arabic (6)
- French (7)
- Korean (8)
- Other (9) __________________________________________________

End of Block: Professional Practice

Start of Block: Professional Subjective

Q9.1 Which of the following would best describe your level of satisfaction or dissatisfaction in the following areas for 2018?

|  | Completely dissatisfied (1) | Somewhat dissatisfied (2) | Neither satisfied nor dissatisfied (3) | Somewhat satisfied (4) | Completely satisfied (5) | No opinion (6) |
| --- | --- | --- | --- | --- | --- | --- |
| Work activities (1) |  |  |  |  |  |  |
| Income (2) |  |  |  |  |  |  |
| Work-life balance (3) |  |  |  |  |  |  |

Q9.2 How would you quantify your level of satisfaction or dissatisfaction in the following areas for 2018?

|  | Completely dissatisfied | Completely satisfied | No opinion |
| --- | --- | --- | --- |

|  | 0 | 10 | 20 | 30 | 40 | 50 | 60 | 70 | 80 | 90 | 100 |
| --- | --- | --- | --- | --- | --- | --- | --- | --- | --- | --- | --- |

| Work activities () | 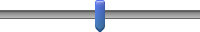 |
| --- | --- |
| Income () | 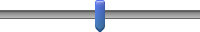 |
| Work-life balance () | 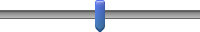 |

Q9.3 Did any of the following create obstacles to attaining greater satisfaction with your work activities in 2018? (select all that apply)

- Personal life (1)
- Family life (2)
- Work activities (3)
- Work environment (personnel) (4)
- Work environment (physical) (5)

Q9.4 Did any of the following create obstacles to attaining greater satisfaction with your income in 2018? (select all that apply)

- Personal life (1)
- Family life (2)
- Work activities (3)
- Work environment (personnel) (4)
- Work environment (physical) (5)

Q9.5 Did any of the following create obstacles to attaining greater work-life satisfaction in 2018? (select all that apply)

- Personal life (1)
- Family life (2)
- Work activities (3)
- Work environment (personnel) (4)
- Work environment (physical) (5)

Q9.6 How would you quantify your stress related to the following areas for 2018?

|  | Not at all stressed | Extremely stressed | No opinion |
| --- | --- | --- | --- |

|  | 0 | 10 | 20 | 30 | 40 | 50 | 60 | 70 | 80 | 90 | 100 |
| --- | --- | --- | --- | --- | --- | --- | --- | --- | --- | --- | --- |

| Your student loan debt () | 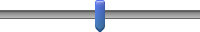 |
| --- | --- |
| Your retirement planning () | 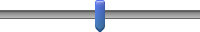 |

End of Block: Professional Subjective

Start of Block: Demographics

Q10.1 The following questions are about demographic factors. 
 
*Please leave blank or choose the relevant response if you would prefer not to respond to these items*

Q10.2 When were you born?

Year (1)

Month (2)

▼ 2001 (1) ... 1929 ~ December (949)

Q10.3 How would you describe your current gender identity? (select all that apply)

- Woman/Female (1)
- Man/Male (2)
- Trans woman/MTF (3)
- Trans man/FTM (4)
- Genderqueer/Gender Non-conforming/Non-binary (5)
- If you prefer a different term, please specify: (6) __________________________________________________
- I prefer not to respond to this item (7)

Q10.4 For research purposes, we often need to group people in a mostly male versus mostly female category.

Which of the following groups would best fit your current gender identity?

- Female (1)
- Male (2)
- I prefer not to respond to this item (3)

Q10.5 How would you describe your race/ethnicity? (select all that apply)

- Latina/o/x or Hispanic or heritage from a Latin American country (1)
- African American/Black (2)
- Native American/American Indian/Indigenous (3)
- Middle Eastern/Arab/Turkish/Iranian (4)
- Asian/Asian American/Pacific Islander (5)
- White/European American (6)
- Biracial/Multiracial (7)
- Other/Please specify, if not captured above: (8) __________________________________________________
- I prefer not to respond to this item (9)

Q10.6 How would you describe your sexual orientation? (select all that apply)

- Heterosexual/Straight (1)
- Lesbian (2)
- Gay (3)
- Bisexual (4)
- Pansexual (5)
- Queer (6)
- Asexual (7)
- If you prefer a different term, please specify: (8) __________________________________________________
- I prefer not to respond to this item (9)

Q10.7 Which of the following physical, mental, or emotional conditions do you experience? (select all that apply)

- Deaf or have serious difficulty hearing (1)
- Blind or have serious difficulty seeing even when wearing glasses (2)
- Serious difficulty concentrating, remembering, or making decisions (3)
- Serious difficulty walking or climbing stairs (4)
- Serious difficulty dressing or bathing (5)
- None of the above (6)
- I prefer not to respond to this item (7)

Q10.8 Have you ever served on active duty in the US Armed Forces, Reserves, or National Guard?

- Never served in the military (1)
- Only on active duty for training in the Reserves or National Guard (2)
- Now on active duty (3)
- On active duty in the past, but not now (4)
- I prefer not to respond to this item (5)

Q10.9 What is your marital status? (select all that apply)

- Now married (1)
- Widowed (2)
- Divorced (3)
- Separated (4)
- Never married (5)
- I prefer not to respond to this item (6)

Display This Question:

If What is your marital status? (select all that apply) = Now married

Or What is your marital status? (select all that apply) = Widowed

Or What is your marital status? (select all that apply) = Divorced

Or What is your marital status? (select all that apply) = Separated

Q10.10 How many times have you been married?

- Once (1)
- Two times (2)
- Three or more times (3)
- I prefer not to respond to this item (4)

Q10.11 In what country did you predominantly grow up?

▼ United States of America (0) ... Zimbabwe (1357)

Q10.12 If applicable, please indicate the zip/postal code(s) where you predominantly grew up.

- (1) __________________________________________________
- (2) __________________________________________________
- (3) __________________________________________________

Q10.13 How would you describe where you predominantly grew up? (select all that apply)

- Large metropolitan area (i.e., population 1.5 million or more) (1)
- Metropolitan area (i.e., population between 500,000 and 1.5 million) (2)
- Medium-size urban area (i.e., population between 200,000 and 500,000) (3)
- Small urban area (i.e., population between 50,000 and 200,000) (4)
- Rural area (i.e., population 50,000 or less) (5)
- If you prefer a different term, please specify: (6) __________________________________________________
- I prefer not to respond to this item (7)

Q10.14 In what country do you currently reside?

▼ United States of America (0) ... Zimbabwe (1357)

Q10.15 If different from where you grew up, in what year did you come to live in the country where you currently reside?

Year (1)

▼ 2018 (1) ... 1901 (118)

Q10.16   If applicable, please indicate the zip/postal code(s) where you currently reside.

- (1) __________________________________________________
- (2) __________________________________________________
- (3) __________________________________________________

Q10.17 How would you describe where you currently reside? (select all that apply)

- Large metropolitan area (i.e., population 1.5 million or more) (1)
- Metropolitan area (i.e., population between 500,000 and 1.5 million) (2)
- Medium-size urban area (i.e., population between 200,000 and 500,000) (3)
- Small urban area (i.e., population between 50,000 and 200,000) (4)
- Rural area (i.e., population 50,000 or less) (5)
- If you prefer a different term, please specify: (6) __________________________________________________
- I prefer not to respond to this item (7)

Q10.18 With whom do you currently reside? (select all that apply and enter the number of individuals)

- Spouse/partner(s) (1) __________________________________________________
- Dependent children (e.g., under 18 years old) (2) __________________________________________________
- Adult children (e.g., over 18 years old) (3) __________________________________________________
- Parent(s) (4) __________________________________________________
- Grandparent(s) (5) __________________________________________________
- Other extended family member(s) (6) __________________________________________________
- Other individuals (non-family) (7) __________________________________________________
- I prefer not to respond to this item (8)

Q10.19 Do you speak any language(s) other than English at home?

- Yes (1)
- No (2)
- I prefer not to respond to this item (3)

Display This Question:

If Do you speak any language(s) other than English at home? = Yes

Q10.20 What language(s) other than English do you speak at home? (select all that apply)

- Spanish (1)
- Mandarin (2)
- Cantonese (3)
- Tagalog (4)
- Vietnamese (5)
- Arabic (6)
- French (7)
- Korean (8)
- Other (9) __________________________________________________
- I prefer not to respond to this item (10)

Q10.21 When you were growing up, did your family ever receive or qualify for public assistance, such as Medicaid, Free or Reduced Lunch, Food Stamps (SNAP), or housing assistance?

- Yes (1)
- No (2)
- I don’t know (3)
- I prefer not to respond to this item (4)

Q10.22
What was the highest level of education of your primary parent(s)/caregiver(s) as a child? 
 
*Enter as many as apply to your childhood.*

Parent/caregiver 1 (1)

Education level (2)

▼ Mother (1) ... Other ~ I do not know (60)

Q10.23

Parent/caregiver 2 (1)

Education level (2)

▼ Mother (1) ... Other ~ I do not know (60)

Q10.24

Parent/caregiver 3 (1)

Education level (2)

▼ Mother (1) ... Other ~ I do not know (60)

Q10.25

Parent/caregiver 4 (1)

Education level (2)

▼ Mother (1) ... Other ~ I do not know (60)

Q10.26

Parent/caregiver 5 (1)

Education level (2)

▼ Mother (1) ... Other ~ I do not know (60)

End of Block: Demographics

Start of Block: Donation (follow up)

Q11.1 Earlier you responded that, if given $1000 to donate to several causes in forensic psychology, you would donate the money in the following manner. Please review your responses and make any changes that you would like before continuing.    *Any updated responses must also equal $1000*

- American Psychology-Law Society (AP-LS) Student Travel Awards: ${Q3.1/ChoiceNumericEntryValue/1} : _______ (1)
- American Society of Trial Consultants (ASTC): ${Q3.1/ChoiceNumericEntryValue/2} : _______ (2)
- Community for Psychologists in Independent Practice (Div42) Fund: ${Q3.1/ChoiceNumericEntryValue/3} : _______ (3)
- National Register of Health Service Psychologists (National Register) Awards: ${Q3.1/ChoiceNumericEntryValue/4} : _______ (4)

Total : ________

Q11.2 At the end of data collection, a total of $1000 (funded by the Principal Investigator) will be donated to these organizations based directly on the averaged responses of all participants (e.g., $500 for 50%, $250 for 25%). The final amounts donated will be reported in all publications and presentations related to this research.

End of Block: Donation (follow up)

Start of Block: Exit

Q12.1 How did you learn about this survey? (select all that apply)

- American Psychology-Law Society email list (1)
- American Academy of Forensic Psychology listserv (2)
- American Society of Trial Consulting listserv (3)
- CONCEPT Professional Training email list (4)
- Div42 listserv (5)
- HSP-Community listserv (6)
- PsyLawList listserv (7)
- Word of mouth (8)
- Other (9) __________________________________________________
- I prefer not to respond to this item (10)

Q12.2 Constructing a survey for diverse professionals can be challenging. Which of the following best describes your view about about the current survey’s ability to capture important information related to your training, practice, and income?

- Completely satisfied (1)
- Somewhat satisfied (2)
- Neither satisfied nor dissatisfied (3)
- Somewhat dissatisfied (4)
- Completely dissatisfied (5)
- No opinion (6)

Q12.3 What changes would you make to future income surveys to improve its ability to capture important information related to your training, practice, and income?

________________________________________________________________

________________________________________________________________

________________________________________________________________

________________________________________________________________

________________________________________________________________

End of Block: Exit
